# Gram-Scale Total Synthesis of TAB with Cardioprotective Activity and the Structure-Activity Relationship of Its Analogs

**DOI:** 10.3390/molecules28135197

**Published:** 2023-07-04

**Authors:** Zhonghao Sun, Zhaocui Sun, Daoshun Wu, Fan Yi, Haifeng Wu, Guoxu Ma, Xudong Xu

**Affiliations:** 1 Institute of Medicinal Plant Development, Chinese Academy of Medical Sciences & Peking Union Medical College, Beijing 100193, China; sun_zhonghao@126.com (Z.S.); flydancingsun@163.com (Z.S.); wudaoshun17@163.com (D.W.); hfwu@implad.ac.cn (H.W.); 2 Beijing Key Laboratory of Plant Resources Research and Development, Beijing Technology and Business University, No. 11/33, Fucheng Road, Beijing 100048, China; fantasyee@btbu.edu.cn

**Keywords:** cardioprotective effect, MIRI, structure-activity relationship, traditional Chinese medicine, total synthesis

## Abstract

Traditional Chinese medicine has been proven to be of great significance in cardioprotective effects. *Clinopodium chinense* (*Lamiaceae*) has unique advantages in the treatment and prevention of cardiovascular diseases. Tournefolic acid B (TAB) was proven to be a potent component against myocardial ischemia reperfusion injury (MIRI) from *Clinopodium chinense* (*Lamiaceae*). This article will attempt to establish a gram-scale synthesis method of TAB and discuss the structure-activity relationship of its analogs. The total synthesis of TAB was completed in 10 steps with an overall yield of 13%. In addition, analogs were synthesized, and their cardioprotective activity was evaluated on the hypoxia/reoxygenation of H9c2 cells. Amidation of the acid position is helpful to the activity, while methylation of phenolic hydroxyl groups greatly decreased the cardioprotective activity. The easily prepared azxepin analogs also showed cardioprotective activity. Most of the *c*logP values calculated by Molinspiration ranged from 2.5 to 5, which is in accordance with Lipinski’s rule of 5. These findings represent a novel kind of cardioprotective agent that is worthy of further study.

## 1. Introduction

Cardiovascular diseases (CVDs) are the leading cause of death globally, which causes one-third of all deaths globally [1]. Ischemic cardiovascular diseases remain the leading cause of death for their high morbidity and mortality, which are also called coronary artery diseases (ACD) [2]. Restoration of blood flow by clinical procedures such as thrombolysis is the standard treatment for attenuating myocardial damage. However, the myocardial reperfusion of ischemia may cause further cardiomyocyte damage, which is known as myocardial ischemia reperfusion injury (MIRI) [3]. Multiple factors are considered to be related to MIRI, including oxidative stress [4], intracellular calcium overload [5], inflammation [6], and apoptosis [7]. The complicated mechanism of MIRI makes it difficult to discover clinically effective treatments and drugs.

Traditional Chinese medicine and phenolic acid components have been proven to be of great significance in cardioprotective effect, such as salvianolic acid B [8], ursolic acid [9], asiatic acid [10], eugenol [11], geniposide [12], quercetin [13], and gastrodin [14]. These active ingredients can exert myocardial protective effects by inhibiting myocardial cell apoptosis, inhibiting inflammatory factor secretion, and regulating the oxidative stress response, which makes them important resources in new drug development.

**Figure 1:**
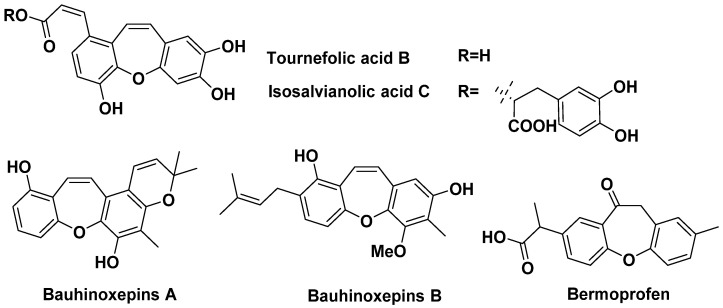
Bioactivity dibenzo[*b,f*]oxepin structures.

*Clinopodium chinense* (*Lamiaceae*) has unique advantages in the treatment and prevention of cardiovascular diseases. We have found that the active ingredient tournefolic acid B (TAB) showed significant protective effects against I/R injuries, which could reduce cell damage induced by hypoxia and reoxygenation at 0.5 ug/mL in rats [15]. The mechanism study illustrated the suppression of TAB on ER stress and oxidative stress via PI3K/AKT pathways [16]. TAB possessed a tricyclic dibenzo[*b,f*]oxepin skeleton with phenols and unsaturated acid (Figure 1), which was first isolated from the aqueous ethanolic extract of *Tournefortia sarmentosa* from Taiwan [17]. In a previous study, Shiao’s group found that TAB showed pharmacological effects on the central nervous system. Specifically, TAB could attenuate amyloid *β* protein-mediated toxicity by abrogating calcium overload in mitochondria and retarding the caspase 8-truncated Bid-cytochrome c pathway in rat cortical neurons [18]. Furthermore, they researched the effects of the ester derivatives of TAB and found that the derivatives could attenuate *N-methyl*-D-aspartate-mediated excitotoxicity [19]. These findings have driven us to explore the special dibenzo[*b,f*]oxepin scaffold and its various pharmacological activities and clinical use.

Bauhinoxepins A and B (Figure 1) were found in the root extract of *Bauhinia saccocalyx*., which exhibited antimycobacterial activities with respective minimum inhibitory concentrations (MIC) of 6.25 and 12.5 mg/mL and showed no cytotoxicity [20]. Isosalvianolic acid C (Figure 1) was discovered in the aqueous extract of *Salvia chinensis* [21]. The following studies showed that isosalvianolic acid C could induce pseudo-allergic reactions mediated by the activation of PLC-γ, IP3R, PKC, and P38 [22]. Bermoprofen (Figure 1) is a nonsteroidal anti-inflammatory drug developed by Dainippon Company that has outstanding effects in the treatment of surgical pain [23]. However, the skeleton of dibenzo[*b,f*]oxepin has rarely been reported to represent cardioprotective effects up to now. The newly found cardioprotective agent TAB was low-content in nature, which makes it urgent to research the total synthesis. In addition, this article will conduct the structural modification at the acid group and oxepin skeleton and briefly discuss the structure-activity relationship.

## 2. Results

### 2.1. Total Synthesis of TAB

#### 2.1.1. Retrosynthesis of TAB

**Figure 2:**
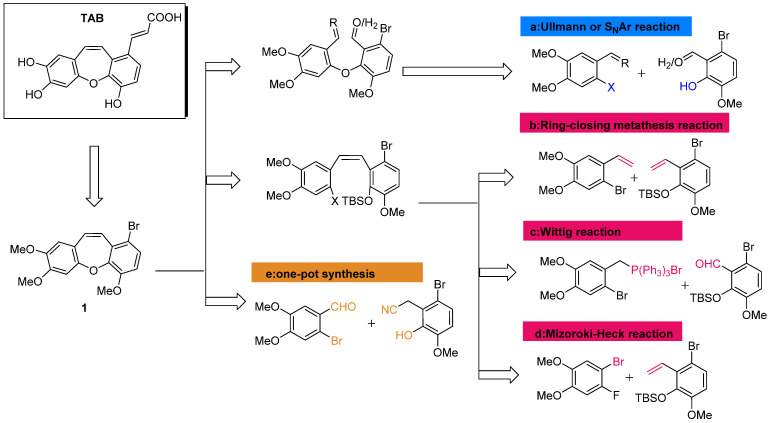
Retrosynthetic analysis TAB and trails of dibenzo[*b,f*]oxepin skeleton construction.

The key structure is the dibenzo[*b,f*]oxepin skeleton, which was usually built by the formation of the diaryl ether and stilbene groups. As shown in Figure 2, we initially tried Ullmann coupling [24] and SNAr reaction of aryl fluoride to form the diaryl ether [25,26] and used the Mizoroki–Heck reaction [27,28], Wittig reaction [29], and ring-closing metathesis [30] to form the stilbene structure. The one-pot synthesis method was also applied according to the literature [31,32]. Due to the multiple oxygenated and halogenated substitutions, route a/b/d reacted poorly, with massive by-products and low yield. Route c fully reacted but yielded up to 90% trans-stilbene product. 

**Scheme 1:**
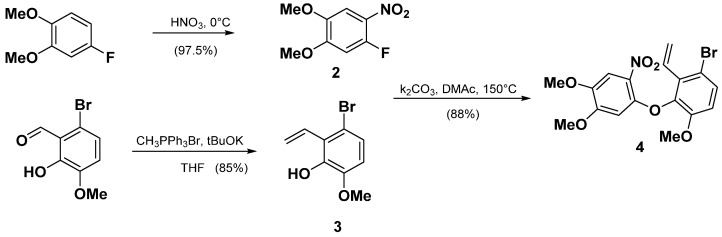
Synthesis of **4** by SNAr reaction.

**Scheme 2:**
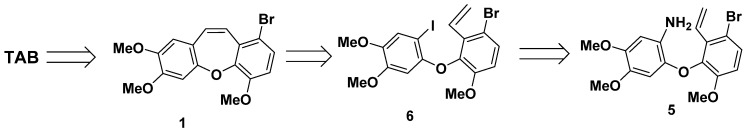
Retrosynthetic route of TAB synthesis of TAB.

Occasionally, we found that the electron-withdrawing nitro material **2** was easily prepared and could efficiently couple with different kinds of phenols by SNAr reactions; for instance, diaryl ether 4 could efficiently be synthesized from **2** and allylphenol 3 (Scheme 1). Based on these findings, we envisioned a new retrosynthetic route shown in Scheme 2. TAB was expected to be obtained from bromide dibenzo[b,f]oxepin 1 by the Heck reaction. Dibenzo[b,f]oxepin 1 will be prepared by an intramolecular Heck reaction from 6. Compound 6 can be prepared by the classical Sandmeyer reaction from arylamine 5, which was reduced from 4.

#### 2.1.2. Synthesis of TAB

4-fluoroveratrole was treated with nitric acid to obtain **2** with an almost quantitative yield. Compound **3** was prepared by 6-bromo-2-hydroxy-3-methoxybenzaldehyde and methyltriphenylphosphonium bromide via the Wittig reaction. Compounds **2** and **3** were reacted efficiently to form the diaryl ether **4** in 88% yield. The nitro reduction in 4 was conducted by either Fe/HCl or SnCl_2_. The post-processing of reagent SnCl_2_ was easier by comparison. Compound **5** was prepared in several grams with a yield of 80%.

Next, the synthesis of stilbene encountered some unexpected situations. When treated with sulfonic acid, sodium nitrite, and potassium iodide, **5** converted not only into iodide **6** (40% yield) but also into an annulated product **7** (45% yield). In the following coupling, **6** failed to form the oxepin by Heck reaction, unfortunately preferring to afford the exo-product **8**. With the two high-yield xanthene products **7** and **8** in hand, we adjusted the route based on a ring expansion method.

**Scheme 3:**
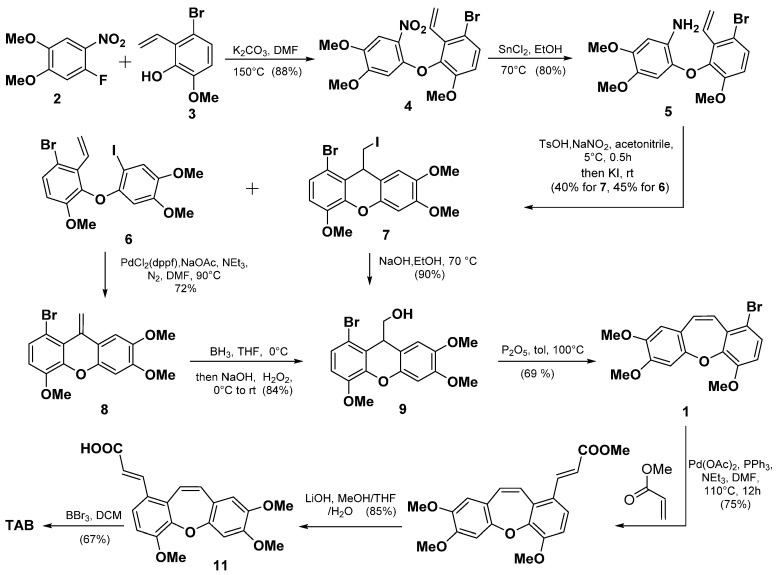
Total synthesis of TAB.

As is shown in Scheme 3, **7** and **8** turned into **9** via hydrolysis and hydroboration-oxidation, respectively. It has been reported oxyanthracyl-9 methanol could rearrange into oxepins in lewis acid [33,34]. As a consequence, we treated **9** with phosphorusoxide and fortunately obtained the desired intermediate **1** in a 69% yield.

Finally, we conducted the attachment of the unsaturated acid chain, as shown in Scheme 3. The Heck reaction conditions were optimized, and the best result was achieved when 1 (1 equiv., methyl acrylate (3 equiv.), PPh_3_ (5 mol%), Et_3_N (5 equiv.), and Pd (OAc)_2_ (5 mol%) were refluxed at 110 °C for 12 h in the solvent DMF under an argon atmosphere to yield 10 in 75% overall yield. After hydrolysis by LiOH and demethylation with BBr_3_, 10 was successfully converted into TAB. All spectroscopic data of TAB were in accordance with the reported literature [17], the NMR and MS spectrums were listed as Figures S34–S36 in the Supplementary Materials.

In all, we have developed a 10-step synthesis of TAB with an overall yield of 13%. Though the formation of the core oxepin structure underwent a ring expansion detour, the whole scheme was efficient, and gram-scale preparation of TAB was achieved.

### 2.2. Synthesis of TAB Derivatives

In order to investigate the influence of stilbene, diaryl ether, and acid functional groups on the cardioprotective effects, we carried out preliminary structural modification at the acid and azxepin groups of TAB.

**Scheme 4:**
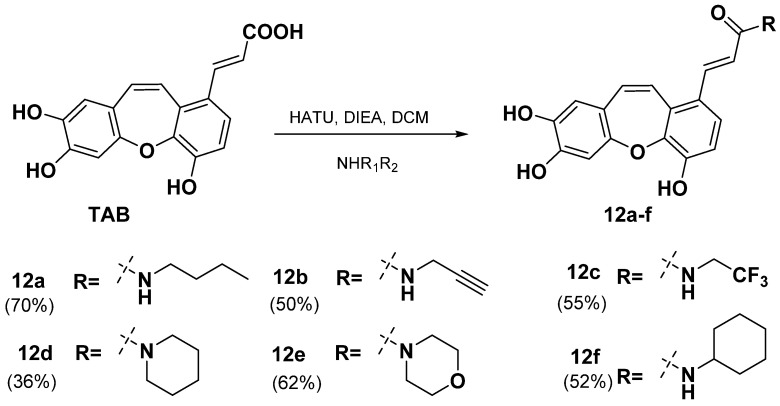
Synthesis of the derivatives **12a**–**f**.

**Scheme 5:**
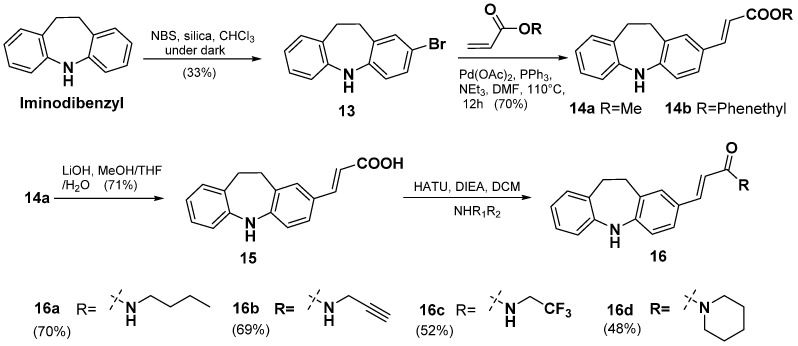
Synthesis of the dihydro-5H-dibenzo[*b,f*]azepin and derivatives.

Two series of analogs were synthesized, including the amide derivatives of TAB and azepine iminodibenzyl. The synthesis and structure of these derivatives are outlined in Scheme 4 and Scheme 5.

### 2.3. Cardioprotective Activity

**Figure 3:**
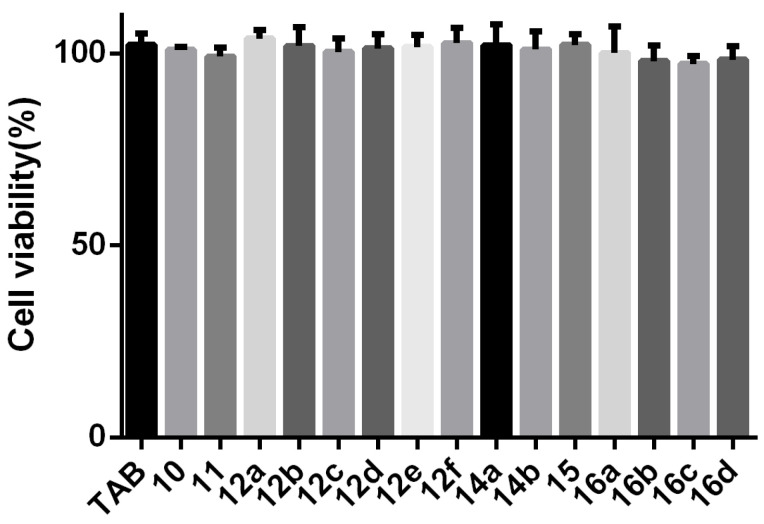
Effect of derivatives on cardiomyocyte cell viability. All values are expressed as the mean ± SD, n = 5.

First of all, we tested the effects of TAB derivatives on cardiomyocytes. Cell viability in H9c2 cardiomyocytes was assessed with the treatment of 10 μM derivative using CCK8 assay. As shown in Figure 3, all the samples did not inhibit the cell growth, which suggested that concentrations within 10 μM are safe for further studies.

Then, in vitro screening of MI/R injury was conducted to evaluate the cardioprotective activities against MI/R injury. Hypoxia/reoxygenation in cardiomyocytes was used as the screening model for MI/R injury. TAB derivatives at five concentrations (0.5, 1, 2, 4, 8 μM) were incubated for 24 h, and cell viability is shown in Table 1.

### 2.4. Structure-Activity Relationship

All the amide derivatives showed relatively potent activity compared to **12a**–**f** with TAB Compound **12c** showed the highest cardioprotective activity at the concentration of 2 μM. In addition, the amide derivatives could protect the cardiomyocyte at a relatively low concentration, which suggested that the amidation of acid position is helpful to the activity. To our surprise, the azxepin derivatives **15** and **16a**–**d** also showed cardioprotective activity but were weaker than TAB and **12a**–**f**. We could find the same increased trend with the amidation of the azxepin; that is, amide derivatives showed a more potent protective effect at a lower concentration. In addition, the esterification of unsaturated acid compounds (**10**, **14a**, **14b**) almost showed no cardioprotective efficacy.

Evaluation of TAB and **11** indicated that methylation of the three phenolic hydroxyl groups greatly decreased the cardioprotective activity. All these results suggested that hydroxyl groups were important for the activity. As is known to all, oxidative stress and high levels of ROS is the main cause of myocardial injury. The phenolic hydroxyl group probably protects myocardial cells by reducing oxygen free radicals.

The *c*logP values of all the compounds were calculated by Molinspiration. Most of the *c*logP values ranged from 2.5 to 5, which is in accordance with Lipinski’s rule of 5. The compound **14b,** with the highest *c*logP value, showed no cardioprotective activity. The phenomenon remaindered us to find azxepin **15** and **16a**–**d** with relatively higher *c*logP values exhibited weaker cardioprotective efficacy compared with the ozxepin series. These findings are helpful in directing further research.

## 3. Discussion

A 10-step total synthesis of TAB was completed with an overall yield of 13%, and gram-scale preparation can be achieved. The application of the route will solve the source issue of TAB and promote further research.

The structure-activity relationship suggested that the amidation of acid position is helpful to the activity, and **12c** showed the most protective effect. The phenolic hydroxyls were important to the activity, and methylation of phenolic hydroxyl groups greatly decreased the cardioprotective activity. The easily prepared azxepin analogs also showed cardioprotective activity, which is worthy of further study. The calculated *c*logP values of most compounds ranged from 2.5 to 5, which is in accordance with Lipinski’s rule of 5. This article primarily discussed the structure-activity relationship. Further intensive structural modification study was in progress, which will help to find more potent cardioprotective effect agents.

## 4. Materials and Methods

### 4.1. General

The products were purified by column chromatography on silica gel (200~300 mesh, Qingdao Marine Chemical plant, Qingdao, China). Precoated silica gel GF254 plates (Zhi Fu Huang Wu Pilot Plant of Silica Gel Development, Yantai, China) were needed for TLC. High-resolution mass spectrometry (HR-ESI-MS) was conducted with a ThermoFisher Scientific LTQ-Orbitrap XL spectrometer. ^1^HNMR and ^13^CNMR spectra were acquired with a Bruker AV III 600 NMR spectrometer (chemical shift values are shown as δ values with TMS as the internal standard). Abbreviations are as follows: s (singlet), d (doublet), dd (doublet of doublet), t (triplet), q (quartet), m (multiplet), and bs (broad singlet). Chemical shifts (δ) are given in ppm relative to the solvent residual peak (CDCl_3_, δ = 7.26 ppm, CD_3_OD, δ = 3.3 ppm, DMSO-*d*, δ = 2.5 ppm) as an external standard. The purity of all biologically tested compounds was determined by HPLC to be >95%. HPLC analysis was performed on Waters 2960 equipped with a Kromasil C18 column (4.6 mm × 250 mm, 5 μm), eluting at 1.0 mL/min over 30 min using a water/MeOH from 50% to 100%. All the in vitro tests were carried out in accordance with guidelines evaluated and approved by the Institute of Medicinal Plant Development (IMPLAD).

### 4.2. Synthesis and Characterization Data

1-fluoro-4,5-dimethoxy-2-nitrobenzene (**2**)

To ice-cold nitric acid (15 mL) was added dropwise with stirring 4-fluoro-1,2-dimethoxybenzene (4.66 g, 30 mmol). The mixture was stirred at 0 °C for 30 min. The orange solution was poured over ice, and the resultant light-yellow solid was filtered, washed with water, then dried to yield **2** (5.9 g, 97.5%) [36]. Mp 131–133 °C, light-yellow powder; ^1^HNMR (600 MHz, CDCl_3_) δ 7.61 (d, *J* = 7.1 Hz, 1H, H-6), 6.76 (d, *J* = 12.3 Hz, 1H, H-3), 3.99 (s, 3H, OMe), 3.96 (s, 3H, OMe).^13^CNMR (150 MHz, CDCl_3_) δ 155.09 (d, *J* = 9.6 Hz, C-5), 152.00 (d, *J _C-F_* = 261.0 Hz, C-4), 151.14 (s, C-1), 144.99 (d, *J _C-F_* = 2.5 Hz, C-2), 107.25 (d, *J _C-F_* = 1.9 Hz, C-6), 100.86 (d, *J_C-F_* = 26.8 Hz, C-3), 56.90 (s, OMe), 56.65 (s, OMe). HRMS (ESI): *m/z* calcd. C_8_HFNNaO_4_ [M+Na]^+^: 224.0335, found 224.0352.

3-bromo-6-methoxy-2-vinylphenol (**3**)

Potassium tert-butoxide (5.04 g, 45 mmol) and methyltriphenylphosphonium bromide (16 g, 45 mmol) were dissolved in 100 mL anhydrous THF under N_2_. After reacting for 1 h at room temperature, the mixture was added to the 100 mL anhydrous THF solvent of 6-bromo-2-hydroxy-2-methoxybenzaldehyde (9.24 g, 40 mmol) at −40 °C. After stirring for 2 h at RT, the reaction was filtered through a celite, and the filtrate was concentrated to yield the cured product. Compound **3** (8.1 g, 85%) was obtained by silica gel column chromatography (petroleum ether:DCM, 10:1). Mp 95–97 °C, light-yellow oil; ^1^HNMR (600 MHz, CDCl_3_) δ 7.12 (d, *J* = 8.6 Hz, 1H, Ar-H), 6.86 (dd, *J* = 17.8, 11.9 Hz, H-8a), 6.66 (d, *J* = 8.6 Hz, 1H, Ar-H), 6.18 (s, 1H, OH), 6.13 (dd, *J* = 17.8, 1.5 Hz, 1H, H-8b), 5.66 (dd, *J* = 11.9, 1.5 Hz, H-7), 3.92 (s, 3H, OMe). ^13^CNMR (150 MHz, CDCl_3_) δ 146.02, 144.91, 132.13, 123.32, 121.43, 115.81, 110.24, 56.33 (OMe). HRMS (ESI): *m/z* calcd. C_9_H_9_BrNaO_2_ [M+Na]^+^: 250.9684, found 250.9692.

1-bromo-3-(4,5-dimethoxy-2-nitrophenoxy)-4-methoxy-2-vinylbenzene (**4**)

Compounds **2** (5.9 g, 29 mmol) and **3** (7.3 g, 30 mmol) were dissolved in 50 mL of N, N-dimethylacetamide, and potassium carbonate (8.2 g, 60 mmol). After refluxing at 150 °C for 5 h, the mixture was extracted with ethyl acetate, washed with water, dried (Na_2_SO_4_), and concentrated under reduced pressure. The crude product was purified by silica gel column chromatography (petroleum ether:ethyl acetate, 6:1) to yield **4** (3.00 g, 88%). Mp 143–145 °C, light-yellow powder; ^1^HNMR (600 MHz, CDCl_3_) δ 7.63 (s, 1H, H-1), 7.48 (d, *J* = 8.9 Hz, 1H, H-9), 6.81 (d, *J* = 8.9 Hz, 1H, H-8), 6.64 (dd, *J* = 17.8, 11.8 Hz, 1H, H-13), 6.03 (s, 1H, H-4), 5.96 (dd, *J* = 17.8, 1.3 Hz, 1H, H-14a), 5.51 (dd, *J* = 11.8, 1.3 Hz, 1H, H-14b), 3.92 (s, 3H, OMe), 3.73 (s, 3H, OMe), 3.69 (s, 3H, OMe). ^13^CNMR (150 MHz, CDCl_3_) δ 154.50, 151.70, 147.87, 143.22, 140.74, 132.42, 130.62, 130.42, 130.37, 123.03, 114.93, 112.65, 108.26, 98.57, 56.49 (OMe), 56.38 (OMe), 56.36 (OMe). HRMS (ESI): *m/z* calcd. C_17_H_16_BrNNaO_6_ [M+Na]^+^: 432.0059, found 432.0067.

2-(3-bromo-6-methoxy-2-vinylphenoxy)-4,5-dimethoxyaniline (**5**)

Compound **4** (10.9 g, 25 mmol) was dissolved in 100 mL of anhydrous ethanol, Tin(II) dichloride dihydrate (11.2 g, 50 mmol), and 0.5 mL of concentrated hydrochloric acid successively. After being heated at 70 °C for 12 h, the mixture was quenched by the addition of 2N sodium hydroxide solution until the PH was adjusted to 10. The mixture was evaporated to remove the bulk ethanol, then extracted with ethyl acetate and concentrated under reduced pressure. Crude product was purified by silica gel column chromatography (petroleum ether:ethyl acetate, 3:1) to yield **5** (7.5 g, 80%). Colorless oil; ^1^HNMR (600 MHz, CDCl_3_) δ 7.44 (d, *J* = 8.8 Hz, 1H, H-9), 6.79 (d, *J* = 8.9 Hz, 1H, H-8), 6.69 (dd, *J* = 17.8, 11.8 Hz, 1H, H-13), 6.45 (s, 1H, H-4), 6.06 (s, 1H, H-1), 5.85 (dd, *J* = 17.8, 1.6 Hz, 1H, H-14a), 5.52 (dd, *J* = 11.8, 1.6 Hz, 1H, H-14b), 3.83 (s, 3H, OMe), 3.74 (s, 3H, OMe), 3.63 (s, 3H, OMe).^13^CNMR (150 MHz, CDCl_3_) δ 152.30, 144.63, 142.82, 141.27, 138.88, 132.74, 131.31, 129.40, 129.21, 122.02, 114.63, 112.50, 101.79, 101.07, 57.19 (OMe), 56.30 (OMe), 56.30 (OMe). HRMS (ESI): *m/z* calcd. C_17_H_18_BrNNaO_4_ [M+Na]^+^: 402.0317, found 402.0324.

1-bromo-3-(2-iodo-4,5-dimethoxyphenoxy)-4-methoxy-2-vinylbenzene (**6**) and 1-bromo-9-(iodomethyl)-4,6,7-trimethoxy-9H-xanthene (**7**)

Compound **5** (7.5 g, 20 mmol) and *p*-Toluenesulfonic acid monohydrate (11 g, 60 mmol) were dissolved in 100 mL of acetonitrile. A total of 3 mL of sodium nitrite (2.75 g, 40 mmol) aqueous solution was added dropwise via a syringe over 5 min at 5 °C. After stirring for 30 min, 10 mL of potassium iodide (8.3 g, 50 mmol) aqueous solution was added slowly. After stirring for another 1 h at RT, the reaction was concentrated and extracted with EA. Compounds **6** (3.9 g, 40%) and **7** (4.41 g, 45%) were obtained by silica gel column chromatography (petroleum ether:ethyl acetate, 4:1). Compound 6: Mp 154–156 °C, white powder; ^1^HNMR (600 MHz, CDCl_3_) δ 7.44 (d, *J* = 8.8 Hz, 1H, H-9), 7.22 (s, 1H, H-4), 6.79 (d, *J* = 8.9 Hz, 1H, H-8), 6.63 (dd, *J* = 17.8, 11.8 Hz, 1H, H-13), 5.95 (s, 1H, H-1), 5.90 (dd, *J* = 17.8, 1.4 Hz, 1H, H-14a), 5.51 (dd, *J* = 11.8, 1.4 Hz, 1H, H-14b), 3.84 (s, 3H, OMe), 3.73 (s, 3H, OMe), 3.61 (s, 3H, OMe).^13^CNMR (150 MHz, CDCl_3_) δ 152.21, 151.01, 149.96, 144.75, 142.21, 132.55, 130.88, 129.89, 114.87, 112.83, 98.38, 56.55 (OMe), 56.44 (OMe), 56.12 (OMe). HRMS (ESI): *m/z* calcd. C_17_H_16_BrINaO_4_ [M+Na]^+^: 512.9174, found 512.9164.

Compound **7**: Mp 180–182 °C, white powder; ^1^HNMR (500 MHz, CDCl_3_) δ 7.28 (d, *J* = 8.7 Hz, 1H, H-9), 6.84 (s, 1H, H-4), 6.82 (s, H-1), 6.79 (t, *J* = 8.0 Hz, 1H, H-8), 4.44 (dd, *J* = 7.0, 3.1 Hz, 1H, H-13), 3.94 (s, 3H, OMe), 3.92 (s, 3H, OMe), 3.89 (s, 3H, OMe), 3.51 (dd, *J* = 9.8, 3.1 Hz, 1H, H-14a), 3.45 (dd, *J* = 9.8, 7.0 Hz, 1H, H-14b).^13^CNMR (125 MHz, CDCl_3_) δ 149.38, 147.90, 145.63, 145.630, 143.04, 126.68, 123.69, 113.69, 113.28, 111.86, 110.83, 100.77, 56.56 (OMe), 56.51 (OMe), 56.29 (OMe), 41.14 (C-13), 15.58 (C-14). HRMS (ESI): *m/z* calcd. C_17_H_16_BrINaO_4_ [M+Na]^+^: 512.9174, found 512.9177.

1-bromo-4,6,7-trimethoxy-9-methylene-9H-xanthene (**8**)

Compound **6** (2.92 g,7.5 mmol), Pd (dppf)Cl_2_ (270 mg,0.375 mmol), sodium acetate (1.23 g, 15 mmol), and triethylamine (1.8 mL, 15 mmol) were dissolved in 50 mL of DMF under N_2_. After stirring at 90 °C for 5 h, the reaction was extracted with EA and concentrated under reduced pressure. Compound **8** (1.95 g, 72 %) was obtained by silica gel column chromatography (petroleum ether:ethyl acetate, 4:1). Mp 126–128 °C, white powder; ^1^HNMR (500 MHz, CDCl_3_) δ 7.35 (d, *J* = 8.7 Hz, 1H, H-8), 7.02 (s, 1H, H-4), 6.79 (s, 1H, H-1), 6.72 (d, *J* = 8.8 Hz, 1H, H-9), 6.04 (s, 1H, H-14a), 5.56 (s, 1H, H-14b), 3.93 (s, 3H, OMe), 3.92 (s, 3H, OMe), 3.89 (s, 3H, OMe).^13^CNMR (125 MHz, CDCl_3_) δ 150.31, 148.03, 146.37, 145.16, 143.14, 132.43, 128.86, 122.68, 116.30, 112.06, 111.38, 110.15, 106.06, 100.26, 56.52 (OMe), 56.51 (OMe), 56.38 (OMe). HRMS (ESI): *m/z* calcd. C_17_H_15_BrNaO_4_ [M + Na]^+^: 385.0054, found 385.0051.

(1-bromo-4,6,7-trimethoxy-9*H*-xanthen-9-yl)methanol (**9**)

Procedure A: **8** (1.92 g, 4 mmol) was dissolved in 10 mL anhydrous THF under N_2_ at 0 °C. A solution of borane ether (1 M, 8 mL) was added. After stirring for 4 h, the reaction was slowly added to a mixture of 30% hydrogen peroxide (2.5 mL) and sodium hydroxide (1 M, 5.5 mL) at 0 °C. Upon completion of TLC analysis, the mixture was concentrated under reduced pressure. Crude product was purified by silica gel column chromatography (petroleum ether:ethyl acetate, 1:1) to yield **9** (1.28 g, 84%).

Procedure B: **7** (4.4 g, 9 mmol) was dissolved in 20 mL of anhydrous ethanol, followed by sodium hydroxide (360 mg,9 mmol). The reaction was refluxed for 1 h. The mixture was concentrated under reduced pressure. Crude product was purified by silica gel column chromatography (petroleum ether:ethyl acetate, 1:1) to yield **9** (3.08 g, 90%).

Compound **9**: Mp 156–158 °C, white solid; ^1^HNMR (500 MHz, CDCl_3_) δ 7.30 (d, *J* = 8.9 Hz, 1H, H-8), 6.87 (s, 1H, H-4), 6.81 (s, 1H, H-1), 6.78 (d, *J* = 8.8 Hz, 1H, H-9), 4.32 (dd, *J* = 6.6, 4.0 Hz, 1H, H-13), 3.95 (s, 3H, OMe), 3.92 (s, 3H, OMe), 3.90 (s, 3H, OMe), 3.87 (dd, *J* = 10.9, 3.7 Hz, 1H, H-14a), 3.72 (dd, *J* = 10.7, 6.7 Hz, 1H, H-14b).^13^CNMR (125 MHz, CDCl_3_) δ 149.19, 147.75, 146.05, 145.77, 143.55, 126.61, 122.38, 113.96, 112.58, 111.63, 110.88, 100.98, 67.19 (C-14), 56.61 (OMe), 56.44 (OMe), 56.34 (OMe), 42.74 (C-13). HRMS (ESI): *m/z* calcd. C_17_H_12_NaO_6_ [M+Na]^+^: 403.0157, found 403.0131.

1-bromo-4,7,8-trimethoxydibenzo[*b,f*]oxepine (**1**)

Compound **9** (4.0 g, 10 mmol) and P_2_O_5_ (7.0 g, 50 mmol) were dissolved in 50 mL of toluene. After refluxing for 1 h, the reaction was quenched with distilled water, extracted with EA, and concentrated under reduced pressure. Crude product was purified by silica gel column chromatography (petroleum ether:ethyl acetate, 1:1) to yield **1** (2.50 g, 69%). Mp 186–188 °C, white solid; ^1^H NMR (500 MHz, CDCl_3_) δ 7.29 (d, *J* = 8.8 Hz, 1H, H-4), 6.94 (d, *J* = 11.4 Hz, 1H, H-12/13), 6.88 (s, 1H, H-8), 6.82 (d, *J* = 11.5 Hz, 1H, H-12/13), 6.79 (d, *J* = 8.8 Hz, 1H, H-1), 6.67 (s, 1H, H-9), 3.92 (s, 3H, OMe), 3.90 (s, 3H, OMe), 3.85 (s, 3H, OMe). ^13^CNMR (125 MHz, CDCl_3_) δ 151.33, 150.71, 146.47, 131.36, 131.19, 128.86, 128.11, 122.19, 113.45, 112.98, 110.70, 105.66, 56.51 (OMe), 56.44 (OMe), 56.39 (OMe). HRMS (ESI): *m/z* calcd. C_17_H_15_BrNaO_4_ [M + Na]^+^: 385.0051, found 385.0093.

Methyl (*E*)-3-(4,7,8-trimethoxydibenzo[*b,f*]oxepin-1-yl)acrylate (**10**)

Compound **1** (3.0 g, 8 mmol), Pd (OAc)_2_ (90 mg, 0.4 mmol), triphenylphosphine (105 mg, 0.4 mmol), triethylamine (1.8 mL, 15 mmol), and methyl acrylate (2.25 mL, 25 mmol) were dissolved in 50 mL of DMF under N_2_. After stirring at 90 °C for 8 h, the reaction was extracted with EA and concentrated under reduced pressure. Compound **10** (2.5 g, 75 %) was obtained by silica gel column chromatography (petroleum ether:ethyl acetate, 2:1). Mp 194–196 °C, white powder; ^1^HNMR (400 MHz, CDCl_3_) δ 7.96 (d, *J* = 18.5 Hz, 1H, H-16), 7.35 (d, *J* = 8.6 Hz, 1H, H-8), 6.97 (d, *J* = 11.4 Hz, 1H, H-12/13), 6.95 (d, *J* = 8.6 Hz, 1H, H-9), 6.94 (s, 1H, H-4), 6.92 (d, *J* = 13.1 Hz, 1H, H-12/13), 6.70 (s, 1H, H-1), 6.28 (d, *J* = 15.8 Hz, 1H, H-15), 3.98 (s, 3H, OMe), 3.92 (s, 3H, OMe), 3.87 (s, 3H, OMe), 3.82 (s, 3H, OMe).^13^CNMR (150 MHz, CDCl_3_) δ 172.04 (C-17), 153.22, 151.49, 150.56, 146.71, 146.27, 143.89, 131.74, 131.18, 124.93, 124.49 (s), 122.26, 117.62, 111.88, 110.45, 105.42, 56.26, 56.22, 56.21, 51.57. HRMS (ESI): *m/z* calcd. C_21_H_20_NaO_6_ [M+Na]^+^: 391.1158, found 391.1198.

(*E*)-3-(4,7,8-trimethoxydibenzo[*b,f*]oxepin-1-yl)acrylic acid (**11**)

Compound **10** (2.2 g, 6 mmol) and LiOH (288 mg, 12 mmol) were dissolved in 5 mL of methanol, 5 mL of THF, and 10 mL of distilled water. After stirring at rt for 12 h, the reaction was concentrated under reduced pressure. Crude product was purified by silica gel column chromatography (petroleum ether:ethyl acetate, 1:1) to yield **11** (1.8 g, 85%). Mp 226–228 °C, white powder; ^1^HNMR (600 MHz, CDCl_3_) δ 8.08 (d, *J* = 15.7 Hz, 1H, H-16), 7.41 (d, *J* = 8.6 Hz, 1H, H-8), 7.03 (d, *J* = 11.5 Hz, 1H, H-12/13), 6.97 (d, *J* = 8.7 Hz, 1H, H-9), 6.92 (d, *J* = 10.5 Hz, 1H, H-12/13), 6.92 (s, 1H, H-4), 6.71 (s, 1H, H-1), 6.31 (d, *J* = 15.7 Hz, 1H, H-15), 4.00 (s, 3H, OMe), 3.93 (s, 3H, OMe), 3.88 (s, 3H, OMe).^13^CNMR (150 MHz, CDCl_3_) δ 172.04 (C-17), 153.22, 151.49, 150.56, 146.71, 146.27, 143.89, 131.74, 131.18, 124.93 (s), 124.90, 124.49, 122.26, 117.62, 111.88, 110.45, 105.42, 56.28 (OMe), 56.22 (OMe), 56.21 (OMe). HRMS (ESI): *m/z* calcd. C_20_H_18_NaO_6_ [M + Na]^+^: 377.1001, found 377.1040.

Tournefolic acid B (TAB)

Compound **11** (1.77 g, 5 mmol) was dissolved in 10 mL of DCM and BBr_3_ (1 M in DCM, 30 mL) at 0 °C. After stirring at room temperature for 2 h, the reaction was concentrated by 5 mL of water, extracted with DCM, and concentrated under reduced pressure. Crude product was purified by silica gel column chromatography (DCM:MeOH, 20:1) to yield TAB (1.05 mg, 67%). Mp 251–253 °C, yellow powder; ^1^HNMR (600 MHz, DMSO) δ 7.95 (d, *J* = 15.8 Hz, 1H, H-16), 7.42 (d, *J* = 8.5 Hz, 1H, H-8), 6.97–6.93 (m, 3H), 6.87 (d, *J* = 11.5 Hz, 1H, H-12/13), 6.70 (s, 1H, H-1), 6.32 (d, *J* = 15.7 Hz, 1H, H-15).^13^CNMR (150 MHz, DMSO) δ 168.91 (C-17), 150.83, 150.67, 147.10, 145.89, 142.47, 141.49, 131.42, 130.99, 124.03, 123.82, 123.70, 121.82, 117.80, 116.47, 114.28, 108.61. HRMS (ESI): *m*/*z* calcd. C_17_H_12_NaO_6_ [M + Na]^+^: 335.0532, found 335.0538.

(*E*)-*N*-butyl-3-(4,7,8-trihydroxydibenzo[*b,f*]oxepin-1-yl)acrylamide (**12a**)

TAB (62 mg, 0.2 mmol) was dissolved in 2 mL of DMF, HATU (91 mg, 0.24 mmol), and DIEA (88 μL, 0.5 mmol). After stirring at room temperature for 5 min, butan-1-amine hydrochloride (27 mg, 0.25 mmol) was added. The mixture was stirred for 2 h at RT and concentrated under reduced pressure. Crude product was purified by silica gel column chromatography (DCM:EA, 10:1) to yield **12a** (51 mg, 70%). Mp 220–222 °C, light-yellow powder; ^1^HNMR (500 MHz, CDCl_3_) δ 7.88 (d, *J* = 15.3 Hz, 1H, H-16), 7.28 (d, *J* = 8.5 Hz,1H, H-8), 7.03 (d, *J* = 11.4 Hz, 1H, H-12/13), 6.91 (d, *J* = 8.5 Hz, 1H, H-9), 6.91 (s, 1H, H-4), 6.85 (d, *J* = 11.5 Hz, 1H, H-12/13), 6.69 (s, 1H, H-1), 6.20 (d, *J* = 15.3 Hz, 1H, H-15), 5.70 (s, 1H, NH), 3.40 (dd, *J* = 13.2, 6.9 Hz, 2H, H-19), 1.58–1.54 (m, 2H, H-20), 1.44–1.38 (m, 2H, H-21), 0.96 (t, *J* = 7.3 Hz, 3H, H-22).^13^CNMR (125 MHz, CDCl_3_) δ 165.96 (C-17), 151.59, 151.07, 147.49, 146.38, 142.86, 141.88, 131.82, 131.25, 124.42, 124.21, 123.90, 122.21, 118.19, 116.87, 114.67, 109.00, 39.70 (C-19), 31.97 (C-20), 20.34 (C-21), 14.01 (C-22). HRMS (ESI): *m/z* calcd. C_21_H_21_NNaO_5_ [M+Na]^+^: 390.1317, found 390.1334.

(*E*)-N-(prop-2-yn-1-yl)-3-(4,7,8-trihydroxydibenzo[*b,f*]oxepin-1-yl)acrylamide (**12b**)

Compound **12b** (39 mg, 50%) was synthesized by the same method as **12a**, except using prop-2-yn-1-amine. Mp 194–196 °C, light-yellow powder; ^1^HNMR (500 MHz, CDCl_3_) δ 7.56 (d, *J* = 15.5 Hz, 1H, H-16), 7.02 (s, 1H, H-4), 6.90–6.86 (m, 2H), 6.69 (d, *J* = 11.3 Hz, 1H, H-12/13), 6.68 (s, 1H, H-1), 6.62 (d, *J* = 11.7 Hz, 2H, H-12/13), 6.30 (d, *J* = 15.5 Hz, 1H, H-15), 5.77 (t, *J* = 5.0 Hz, 1H, NH), 4.19 (dd, *J* = 5.2, 2.5 Hz, 2H, H-19), 2.27 (t, *J* = 2.5 Hz, 1H, H-21).^13^CNMR (125 MHz, CDCl_3_) δ 165.47 (C-17), 149.55, 149.32, 145.75, 144.54, 141.31, 140.14, 130.07, 129.64, 122.68, 122.47, 122.31, 120.47, 116.45, 115.12, 112.93, 107.26, 79.52 (C-20), 72.06 (C-21), 29.66 (C-19C). HRMS (ESI): *m/z* calcd. C_20_H_15_NNaO_5_ [M+Na]^+^: 372.0848, found 372.0871.

(*E*)-N-(2,2,2-trifluoroethyl)-3-(4,7,8-trihydroxydibenzo[*b,f*]oxepin-1-yl)acrylamide (**12c**)

Compound **12c** (43 mg, 55%) was synthesized by the same method as **12a**, except using trifluoroethylamine hydrochloride. Mp 246–248 °C, light-yellow powder; ^1^HNMR (500 MHz, CDCl_3_) δ 7.60 (d, *J* = 15.5 Hz, 1H, H-16), 7.00 (m, 1H, H-8), 6.88 (m, 1H, H-9), 6.85 (s, 1H, H-4), 6.67 (d, *J* = 11.3 Hz, 1H, H-12/13), 6.62 (s, 1H, H-1), 6.57 (d, *J* = 11.3 Hz, 1H, H-12/13), 6.36 (d, *J* = 15.5 Hz, 1H, H-15), 6.10 (t, *J* = 6.4 Hz, 1H, NH), 4.08–4.01 (m, 2H, H-19). ^13^C NMR (125 MHz, CDCl_3_) δ 166.08 (C-17), 150.42, 150.27, 146.69, 145.48, 142.06, 141.08, 131.02, 130.58, 124.35 (q, *J_C-F_* = 276.0 Hz, C-20), 123.62, 123.41, 123.30, 121.41, 117.39, 116.07, 113.87, 108.20, 40.92 (q, *J_C-F_* = 34.7 Hz, C-19). HRMS (ESI): *m/z* calcd. C_19_H_14_F_3_NNaO_5_ [M+Na]^+^: 416.0722, found 416.0756.

(*E*)-1-(piperidin-1-yl)-3-(4,7,8-trihydroxydibenzo[*b,f*]oxepin-1-yl)prop-2-en-1-one (**12d**)

Compound **12d** (28 mg, 36%) was synthesized by the same method as **12a**, except using piperidine. Mp 220–222 °C, light-yellow powder; ^1^HNMR (500 MHz, CDCl_3_) δ 7.56 (d, *J* = 11.5 Hz, 1H, H-12/13), 7.04–6.95 (m, 2H, H-8,9), 6.89 (s, 1H, H-4), 6.82 (d, *J* = 15.4 Hz, 1H, H-16), 6.72 (d, *J* = 11.3 Hz, 1H, H-12/13), 6.67 (s, d, *J* = 15.4 Hz, 1H, H-15), 6.67 (s, 1H, H-1), 3.68 (s, 2H, H-19/23), 3.60 (s, 2H, H-19/23), 1.71 (d, *J* = 4.5 Hz, 2H, H-20/22), 1.62–1.61 (m, 2H, H-20/22), 1.28 (t, *J* = 5 Hz, 2H, H-21).^13^CNMR (125 MHz, CDCl_3_) δ 165.38 (C-17), 15091, 150.77, 147.28, 145.98, 142.56, 141.66, 131.52, 131.08, 124.12, 123.91, 123.80, 121.91, 117.89, 116.57, 114.37, 108.90, 47.23 (C-19), 43.56 (C-23), 29.93 (C-20), 27.00 (C-22), 25.82 (C-21). HRMS (ESI): *m/z* calcd. C_22_H_21_NNaO_5_ [M + Na]^+^: 402.1317, found 402.1331.

(*E*)-1-morpholino-3-(4,7,8-trihydroxydibenzo[*b,f*]oxepin-1-yl)prop-2-en-1-one (**12e**)

Compound **12e** (47 mg, 62%) was synthesized by the same method as **12a**, except using morpholine. Mp 210–212 °C, light-yellow powder; ^1^HNMR (500 MHz, CDCl_3_) δ 7.98 (d, *J* = 15.1 Hz, 1H, H-16), 7.31 (d, *J* = 8.6 Hz, 1H, H-8), 7.02 (d, *J* = 11.4 Hz, 1H, H-12/13), 6.92 (d, *J* = 8.6 Hz, 1H, H-9), 6.91 (s, 1H, H-4), 6.86 (d, *J* = 11.5 Hz, 1H, H-12/13), 6.70 (s, 1H, H-1), 6.65 (d, *J* = 15.1 Hz, 1H, H-15), 3.76 (m, 6H, H of morpholine), 3.66 (m, 2H, H of morpholine).^13^CNMR (125 MHz, CDCl_3_) δ 165.64 (C-17), 149.12, 148.81, 145.39, 144.18, 140.66, 139.78, 129.72, 129.28, 122.32, 122.11, 122.00, 116.09, 114.77, 112.57, 106.98, 67.07 (C-20, 21), 46.47 (C-19/22), 42.69 (C-19/22). HRMS (ESI): *m/z* calcd. C_21_H_19_NNaO_6_ [M+Na]^+^: 404.1110, found 404.1107

(*E*)-N-cyclohexyl-3-(4,7,8-trihydroxydibenzo[*b,f*]oxepin-1-yl)acrylamide (**12f**)

Compound **12f** (41 mg, 52%) was synthesized by the same method as **12a**, except using cyclohexylamine hydrochloride. Mp 178–180 °C, light-yellow powder; ^1^HNMR (500 MHz, CDCl_3_) δ 7.51 (d, *J* = 15.5 Hz, 1H, H-16), 7.02–6.90 (m, 2H), 6.86 (s, 1H, H-4), 6.69 (d, *J* = 11.3 Hz, 1H, H-12.13), 6.63 (s, 1H, H-1), 6.62 (d, *J* = 11.6 Hz, 1H, H-12/13), 6.27 (d, *J* = 15.5 Hz, 1H, H-15), 5.44 (d, *J* = 8.0 Hz, 1H, NH), 1.98 (dd, *J* = 12.2, 2.7 Hz, 2H, H of cyclohexyl), 1.73 (dd, *J* = 10.0, 3.7 Hz, 2H, H of cyclohexyl), 1.64 (dd, *J* = 9.3, 3.7 Hz, 1H, H of cyclohexyl), 1.44–1.36 (m, 2H, H of cyclohexyl), 1.20–1.15 (m, 2H, H of cyclohexyl), 0.86 (dt, *J* = 18.6, 6.3 Hz, 2H, H of cyclohexyl).^13^CNMR (150 MHz, CDCl_3_) δ 164.90 (C-17), 148.64, 148.50, 144.89, 143.68, 140.26, 139.28, 129.22, 128.85, 121.82, 121.61, 121.50, 119.61, 115.59, 114.27, 112.18, 106.40, 48.56 (C-19), 33.48 (C-20, 24), 29.92 (C-22), 25.74 (C-21/23), 25.0625.74 (C-21/23). HRMS (ESI): *m/z* calcd. C_23_H_23_NNaO_5_ [M + Na]^+^: 416.1474, found 416.1479.

2-bromo-10,11-dihydro-5*H*-dibenzo[*b,f*]azepine (**13**)

Iminodibenzyl (1.95 g, 10 mmol) and silica gel (20 g) were dissolved in 40 mL of CHCl_3_. NBS (1.78 g, 10 mmol) was added, and the mixture was stirred in the dark for 1 h. The mixture was filtered, and the filtrate was concentrated under reduced pressure. Crude product was purified by silica gel column chromatography (PE:EA, 10:1) to yield **13** (1.82 g, 33%). Mp 125–127 °C, brown solid; ^1^HNMR (600 MHz, CDCl_3_) δ 7.14 (m, 2H), 7.07 (t, *J* = 7.6 Hz, 1H), 7.03 (d, *J* = 7.4 Hz, 1H), 6.70 (d, *J* = 8.0 Hz, 1H), 6.57 (d, *J* = 8.3 Hz, 1H), 5.95 (s, 1H, NH), 3.09–2.96 (m, 4H, H-7, 8).^13^C NMR (151 MHz, CDCl_3_) δ 142.02, 141.71, 133.20, 130.87, 130.67, 129.63, 128.64, 127.12, 120.02, 119.62, 118.18, 111.35, 34.84 (C-7/8), 34.74 (C-7/8). HRMS (ESI): *m/z* calcd. C_14_H_12_BrNNa [M+Na]^+^: 296.0051, found 296.0079.

Methyl (E)-3-(10,11-dihydro-5H-dibenzo[b,f]azepin-2-yl)acrylate (14a) and phenethyl (E)-3-(10,11-dihydro-5H-dibenzo[b,f]azepin-2-yl)acrylate (14b)

Compound **13** (1.35 g, 5 mmol), Pd (OAc)_2_ (55 mg, 0.25 mmol), triphenylphosphine (65 mg, 0.25 mmol), triethylamine (1.8 mL, 15 mmol), and methyl acrylate (1.35 mL, 15 mmol) were dissolved in 10 mL of DMF under N_2_. After stirring at 90 °C for 12 h, the reaction was extracted with EA and concentrated under reduced pressure. Compound **14a** (1.06 g, 70%) was obtained by silica gel column chromatography (petroleum ether:ethyl acetate, 5:1). Mp 158–160 °C, yellow powder; ^1^HNMR (600 MHz, CDCl_3_) δ 7.60 (d, *J* = 15.9 Hz, 1H), 7.28 (dd, *J* = 8.3, 2.1 Hz, 1H), 7.22 (d, *J* = 1.9 Hz, 1H), 7.11 (t, *J* = 7.6 Hz, 1H), 7.07 (d, *J* = 7.4 Hz, 1H), 6.83 (td, *J* = 7.4, 0.9 Hz, 1H), 6.77 (dd, *J* = 7.9, 0.6 Hz, 1H), 6.71 (d, *J* = 8.3 Hz, 1H), 6.27 (d, *J* = 15.9 Hz, 2H, H-16, 17), 3.79 (s, 3H, Me), 3.08 (s, 4H, H-10, 11).^13^CNMR (150 MHz, CDCl_3_) δ 168.09 (C-18), 144.73, 144.30, 141.36, 131.51, 130.65, 129.27, 127.04, 126.97, 125.25, 120.37, 118.32, 118.27, 113.82, 51.57 (Me), 35.31 (C-10/11), 34.84 (C-10/11). HRMS (ESI): *m/z* calcd. C_18_H_17_NNaO_2_ [M+Na]^+^: 302.1157, found 302.1168.

Compound **14b** (65% yield) was synthesized by the same method as **14a**. Mp 164–166 °C, yellow powder; ^1^HNMR (600 MHz, CDCl_3_) δ 7.61 (d, *J* = 15.9 Hz, 1H, H-16), 7.35 (t, *J* = 7.5 Hz, 2H), 7.30 (m, 3H), 7.27–7.26 (m, 1H), 7.24 (s, 1H), 7.13 (t *J* = 10.9, 1H), 7.10 (d, *J* = 7.4 Hz, 1H), 6.86 (t, *J* = 7.3 Hz, 1H), 6.80 (d, *J* = 7.9 Hz, 1H), 6.74 (d, *J* = 8.3 Hz, 1H), 6.30 (m, 2H), 4.44 (t, *J* = 7.1 Hz, 2H, H-19), 3.10 (s, 4H, H-10, 11), 3.04 (t, *J* = 7.1 Hz, 2H, H-20). ^13^C NMR (125 MHz, CDCl_3_) δ 167.72 (C-18), 144.35, 144.18, 141.37, 131.43, 130.62, 129.23, 127.85, 127.00, 126.91, 125.35, 120.32, 118.27, 118.23, 114.35, 64.17 (C-19), 35.26 (C-10/11), 34.80 (C-10/11), 30.83 (C-20). HRMS (ESI): *m/z* calcd. C_25_H_23_NNaO_2_ [M+Na]^+^: 392.1626, found 392.1654.

(*E*)-3-(10,11-dihydro-5*H*-dibenzo[*b,f*]azepin-2-yl)acrylic acid (**15**)

Compound **14a** (1.0 g, 2.8 mmol) and LiOH (144 mg, 6 mmol) were dissolved in 3 mL of methanol, 3 mL of THF, and 6 mL of distilled water. After stirring at rt for 12 h, the reaction was concentrated under reduced pressure. Crude product was purified by silica gel column chromatography (petroleum ether:ethyl acetate, 1:1) to yield **15** (530 mg, 71%). Mp 194–196 °C, yellow powder; ^1^HNMR (600 MHz, MeOD) δ 7.33 (d, *J* = 15.8 Hz, 1H, H-17), 7.25 (d, *J* = 8.2 Hz, 1H), 7.18 (m, 1H), 7.05–7.00 (m, 2H), 6.91 (m, 2H), 6.73 (t, *J* = 7.3 Hz, 1H), 6.34 (m, 1H), 3.05 (s, 4H, H-10, 11).^13^CNMR (150 MHz, MeOD) δ 175.15 (C-18), 144.00, 142.44, 139.85, 130.04, 129.87, 128.69, 127.54, 126.37, 126.06, 125.70, 120.88, 118.90, 117.80, 117.75, 35.27 (C-10/11), 34.96 (C-10/11). HRMS (ESI): *m/z* calcd. C_17_H_15_NNaO_2_ [M+Na]^+^: 288.1000, found 288.1046.

(*E*)-*N*-butyl-3-(10,11-dihydro-5*H*-dibenzo[*b*,*f*]azepin-2-yl)acrylamide (**16a**)

Compound **15** (26 mg, 0.2 mmol) was dissolved in 1 mL of DMF, HATU (91 mg, 0.24 mmol), and DIEA (88 μL, 0.5 mmol). After stirring at room temperature for 5 min, butan-1-amine hydrochloride (27 mg, 0.25 mmol) was added. The mixture was stirred for 2 h at RT and concentrated under reduced pressure. Crude product was purified by silica gel column chromatography (DCM:EA, 8:1) to yield **16a** (44 mg, 70%). Mp 163–165 °C, light-yellow powder; ^1^HNMR (500 MHz, CDCl_3_) δ 7.55 (d, *J* = 15.5 Hz, 1H, H-17), 7.26 (dd, *J* = 8.3, 1.7 Hz, 1H), 7.21 (d, *J* = 1.4 Hz, 1H), 7.12 (t, *J* = 7.6 Hz, 1H), 7.08 (d, *J* = 7.4 Hz, 1H), 6.83 (t, *J* = 7.4 Hz, 1H), 6.79 (d, *J* = 7.9 Hz, 1H), 6.73 (d, *J* = 8.2 Hz, 1H), 6.28 (d, *J* = 7.3 Hz, 1H), 6.25 (d, *J* = 15.5 Hz, 1H), 5.59 (d, *J* = 6.0 Hz, 1H, NH), 3.41 (dd, *J* = 13.1, 6.9 Hz, 2H, H-20), 3.09 (s, 4H, H-10, 11), 1.59–1.55 (m, 2H, H-21), 1.45–1.38 (m, 2H, H-22), 0.97 (t, *J* = 7.3 Hz, 3H, H-23).^13^CNMR (125 MHz, CDCl_3_) δ 166.65 (C-18), 143.88, 141.78, 140.78, 131.16, 130.83, 129.29, 128.14, 127.18, 126.73, 125.99, 120.33, 118.43, 117.30, 39.65 (C-20), 35.26 (C-10/11), 35.03 (C-10/11), 32.03 (C-21), 29.94 (C-22), 14.03 (C-23). HRMS (ESI): *m/z* calcd. C_21_H_24_N_2_NaO [M+Na]^+^: 343.1786, found 343.1808.

(*E*)-3-(10,11-dihydro-5*H*-dibenzo[*b,f*]azepin-2-yl)-*N*-(prop-2-yn-1-yl)acrylamide (**16b**)

Compound **16b** (42 mg, 69%) was synthesized by the same method as **16a**, except using prop-2-yn-1-amine. Mp 206–208 °C, yellow powder; ^1^HNMR (600 MHz, CDCl_3_) δ 7.59 (d, *J* = 15.9 Hz, 1H, H-17), 7.27–7.26 (m, 1H), 7.21 (d, *J* = 1.3 Hz, 1H), 7.11–7.09 (m, 1H), 7.06 (d, *J* = 7.4 Hz, 1H), 6.82 (t, *J* = 7.3 Hz, 1H), 6.76 (d, *J* = 7.9 Hz, 1H), 6.70 (d, *J* = 8.3 Hz, 1H), 6.26 (d, *J* = 15.9 Hz, 1H, H-16), 6.24 (s, 1H, NH), 4.01 (d, *J* = 2.9 Hz, 2H, H-20), 3.07 (s, 4H, H-10, 11), 2.09 (s, 1H, H-22).^13^CNMR (150 MHz, CDCl_3_) δ 166.27 (C-18), 144.15, 141.86, 141.68, 141.68, 131.39, 130.81, 129.34, 128.10, 127.19, 126.86, 120.41, 118.47, 116.14, 79.94 (C-21), 71.82 (C-22), 53.22 (C-20), 35.48 (C-10/11), 35.04 (C-10/11). HRMS (ESI): *m/z* calcd. C_20_H_18_N_2_NaO [M+Na]^+^: 325.1317, found 325.1336.

(*E*)-3-(10,11-dihydro-5*H*-dibenzo[*b,f*]azepin-2-yl)-*N*-(2,2,2-trifluoroethyl)acrylamide (**16c**)

Compound **16c** (36 mg, 52%) was synthesized by the same method as **16a**, except using trifluoroethylamine hydrochloride. Mp 187–189 °C, yellow powder; ^1^HNMR (500 MHz, CDCl_3_) δ 7.64 (d, *J* = 15.5 Hz, 1H), 7.23 (d, *J* = 5.8 Hz, 1H), 7.13 (t, *J* = 7.7 Hz, 1H), 7.09 (d, *J* = 7.5 Hz, 1H), 6.85 (t, *J* = 7.3 Hz, 1H), 6.79 (d, *J* = 8.0 Hz, 1H), 6.74 (d, *J* = 8.3 Hz, 1H), 6.28 (d, *J* = 15.4 Hz, 2H), 6.28 (s, 1H), 5.77–5.74 (m, 1H, NH), 4.16–4.01 (m, 2H), 3.10 (s, 4H). ^13^CNMR (125 MHz, CDCl_3_) δ 166.58, 144.38, 143.13, 141.56, 131.58, 130.83, 129.43, 128.11, 128.10 (q, *J* = 267.8 Hz), 127.23, 127.06, 125.33, 120.56, 118.49, 118.47, 115.28, 40.92 (q, *J* = 34.6 Hz), 35.48, 35.00. HRMS (ESI): *m/z* calcd. C_19_H_17_F_3_N_2_NaO [M+Na]^+^: 369.1191, found 369.1197.

(*E*)-3-(10,11-dihydro-5*H*-dibenzo[*b,f*]azepin-2-yl)-1-(piperidin-1-yl)prop-2-en-1-one (**16d**)

Compound **16d** (32 mg, 48%) was synthesized by the same method as **16a**, except using piperidine. Mp 192–194 °C, yellow powder; ^1^HNMR (500 MHz, CDCl_3_) δ 7.62 (d, *J* = 15.5 Hz, 1H, H-17), 7.21 (s, 1H), 7.11 (d, *J* = 7.5 Hz, 1H), 7.07 (d, *J* = 7.5 Hz, 1H), 6.83 (t, *J* = 7.3 Hz, 1H), 6.77 (d, *J* = 8.0 Hz, 1H), 6.71 (d, *J* = 8.3 Hz, 1H), 6.36–6.21 (m, 2H), 5.75–5.72 (m, 1H, NH), 3.68 (s, 2H, H-19/23), 3.60 (s, 2H, H-19/23), 3.07 (s, 4H, H-10, 11), 2.02–1.98 (m, 2H), 1.76–1.73 (m, 2H), 1.67–1.64 (m, 2H).^13^CNMR (125 MHz, CDCl_3_) δ 165.72, 143.89, 141.82, 140.68, 131.17, 130.80, 129.28, 129.04, 127.16, 126.68, 126.00, 120.28, 118.46, 117.58, 48.49, 45.96, 35.46, 35.06, 30.76, 29.92, 25.77, 25.10. HRMS (ESI): *m/z* calcd. C_22_H_24_N_2_NaO [M+Na]^+^: 355.1786, found 355.1795.

### 4.3. Cardioprotective Activity Assays

#### 4.3.1. Cell Culture and Treatment

H9c2 cardiomyocytes were obtained from the Cell Bank of the Chinese Academy of Sciences (Shanghai, China). The cells were plated and grown in Dulbecco’s modified Eagle’s medium (DMEM, ThermoFisher Scientific, USA) with 10% (*v*/*v*) fetal bovine serum (FBS, HyClone, South Logan, UT, USA) and 1% penicillin/streptomycin (Sigma-Aldrich, St. Louis, MO, USA) and maintained in a humidified incubator at 37 °C for containing 5% CO_2_.

#### 4.3.2. Hypoxia/Reoxygenation Protocol

H9c2 cardiomyocytes were plated and grown in a humidified incubator at 37 °C for at least 24 h containing 5% CO_2_. For hypoxia/reoxygenation (H/R) processes, cells suffered 6 h of hypoxia and 12 h of reoxygenation as previously described [4]. Briefly, H9c2 cells were cultured in DMEM supplemented with 10% FBS, to which samples were treated with vehicle (0.1% DMSO) or samples (final concentrations: 0, 0.5, 1, 2, 4, and 8 μM). After incubation for 24 h, cells were removed to an anaerobic glove box (Coy Laboratory, Michigan, USA), in which 5% CO_2_ was changed to a combination of 5% H_2_, 5% CO_2_, and 90% N_2_. After 6 h of hypoxia, cells were maintained in the regular incubator for 12 h as reperfusion.

#### 4.3.3. Cell Viability Assay

Cell viability assay was determined using Cell Counting Kit-8 (CCK8) (Dojindo) according to the manufacturer’s protocol. Briefly, 10 μL/well of CCK-8 solution was added to the cells cultured in 96-well plates (5 × 104 cells/well) and kept at 37 °C for 4 h. The absorbance at 450 nm was detected by a microplate reader, and the cell viability was presented by the percentage of CCK-8 reduction relative to that of the control.

## 5. Conclusions

The total synthesis of TAB was completed in 10 steps with an overall yield of 13%. In addition, analogs were synthesized, and their cardioprotective activity was evaluated on hypoxia/reoxygenation of H9c2 cells. Amidation of acid position is helpful to the activity, while methylation of phenolic hydroxyl groups greatly decreased the cardioprotective activity. The easily prepared azxepin analogs also showed cardioprotective activity. The application of the route solved the source issue of TAB, and the structure-activity relationship will promote further drug discovery.

## Figures and Tables

**Table 1:** The effects of TAB derivatives against MI/R injury in H9c2.

Compd.	Cell Viability (% of Control)						*c*logP ^a^
	0 μM	0.5 μM	1 μM	2 μM	4 μM	8 μM	
TAB	56.0 ± 0.7	60.2 ± 0.4	77.2 ± 1.1	68.6 ± 0.9	65.7 ± 0.6	65.5 ± 1.2	2.69
**10**	56.5 ± 1.3	58.2 ± 1.3	56.8 ± 0.9	62.5 ± 1.6	62.0 ± 0.8	59.5 ± 1.4	4.20
**11**	56.3 ± 0.8	60.5 ± 0.7	60.4 ± 0.6	61.7 ± 1.2	67.4 ± 0.5	64.3 ± 0.6	3.58
**12a**	56.6 ± 1.5	72.5 ± 0.5	75.7 ± 1.2	77.3 ± 1.5	74.6 ± 0.9	75.3 ± 1.1	3.99
**12b**	56.4 ± 0.5	68.6 ± 1.5	70.7 ± 1.4	73.8 ± 0.9	77.3 ± 1.1	70.6 ± 0.7	2.71
**12c**	56.7 ± 0.9	73.6 ± 0.5	78.5 ± 0.8	82.6 ± 1.1	80.4 ± 1.3	79.5 ± 0.8	3.48
**12d**	56.5 ± 0.5	71.0 ± 0.6	75.6 ± 1.1	74.6 ± 0.8	72.5 ± 1.6	59.6 ± 1.7	3.70
**12e**	56.1 ± 0.7	69.4 ± 1.2	74.1 ± 1.5	75.9 ± 1.2	73.7 ± 0.7	65.2 ± 0.3	2.64
**12f**	56.7 ± 0.3	63.3 ± 1.3	68.5 ± 0.6	68.3 ± 1.0	66.2 ± 1.6	60.9 ± 1.2	4.46
**14a**	56.9 ± 1.1	55.6 ± 1.0	57.4 ± 1.1	57.1 ± 1.4	59.7 ± 0.4	61.5 ± 1.1	4.62
**14b**	56.4 ± 1.4	58.4 ± 1.6	56.1 ± 1.2	57.4 ± 1.7	58.5 ± 1.8	62.5 ± 0.8	6.41
**15**	56.0 ± 1.2	60.3 ± 1.0	64.8 ± 0.5	71.8 ± 1.4	69.3 ± 1.1	63.9 ± 0.9	4.00
**16a**	55.8 ± 0.5	65.1 ± 0.6	65.7 ± 1.3	72.6 ± 0.3	70.8 ± 1.2	69.4 ± 0.5	5.30
**16b**	56.4 ± 0.8	67.3 ± 0.6	75.0 ± 1.2	72.5 ± 1.7	68.2 ± 0.9	66.3 ± 0.6	4.02
**16c**	56.2 ± 1.6	64.6 ± 1.5	72.4 ± 1.0	75.3 ± 1.2	69.3 ± 0.3	63.3 ± 0.5	4.79
**16d**	56.7 ± 0.5	69.5 ± 1.5	71.4 ± 0.6	72.9 ± 1.1	65.4 ± 0.8	67.2 ± 1.0	5.01

^a^ *c*logP calculated by Molinspiration [35].

## Data Availability

All data are available in the main text or the Supplementary Materials.

## Supplementary Materials

The following supporting information can be downloaded at: https://www.mdpi.com/1420-3049/28/13/5197/s1. Figures S1–S78: ^1^H NMR, ^13^C NMR and HRMS of compounds **2**–**16** and TAB.

## Sample Availability

TAB sample is available. Other samples are not available.
