# Supplementary material for: Gram-Scale Total Synthesis of TAB with Cardioprotective Activity and the Structure-Activity Relationship of Its Analogs
Source: Molecules. 2023 Jul 4;28(13):5197. doi: 10.3390/molecules28135197 (PMC10343337; doi:10.3390/molecules28135197)
Supplement: Supplementary file 1 [file molecules-28-05197-s001.zip › molecules-2458217-supplementary.pdf]

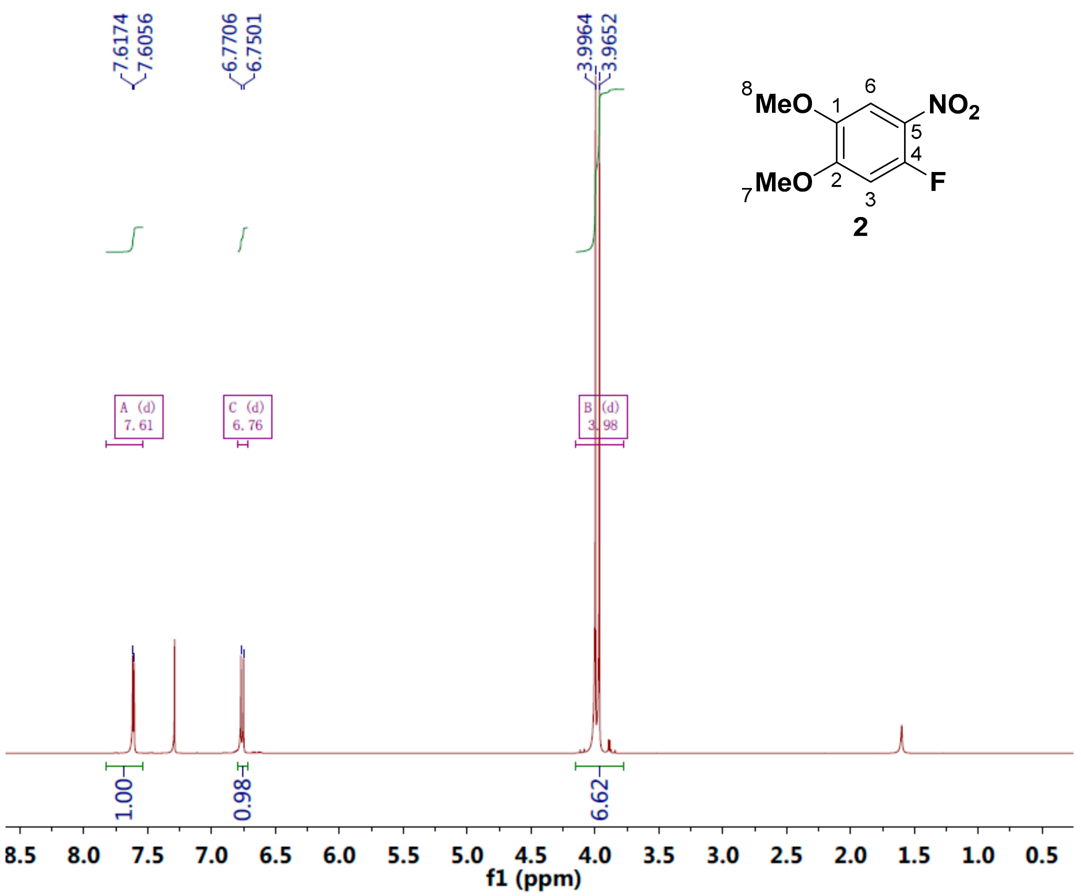

Figure S1. [1] H NMR of compound 2.

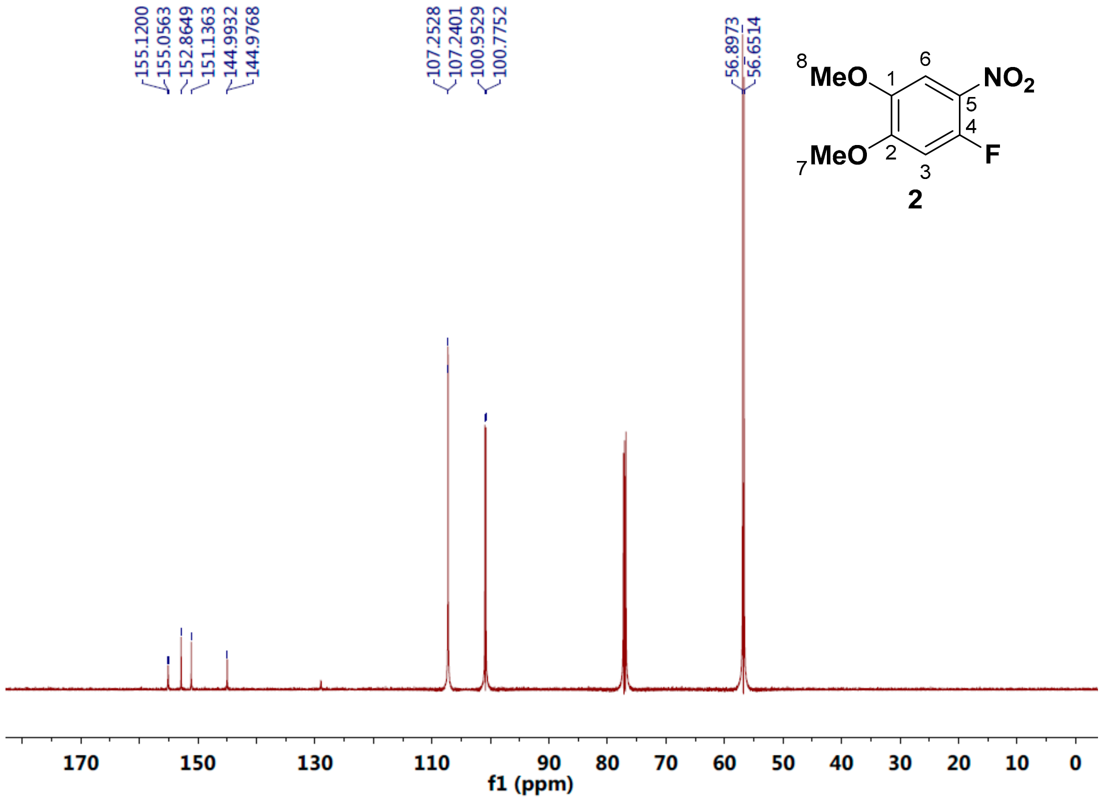

Figure S2. [13] C NMR of compound 2.

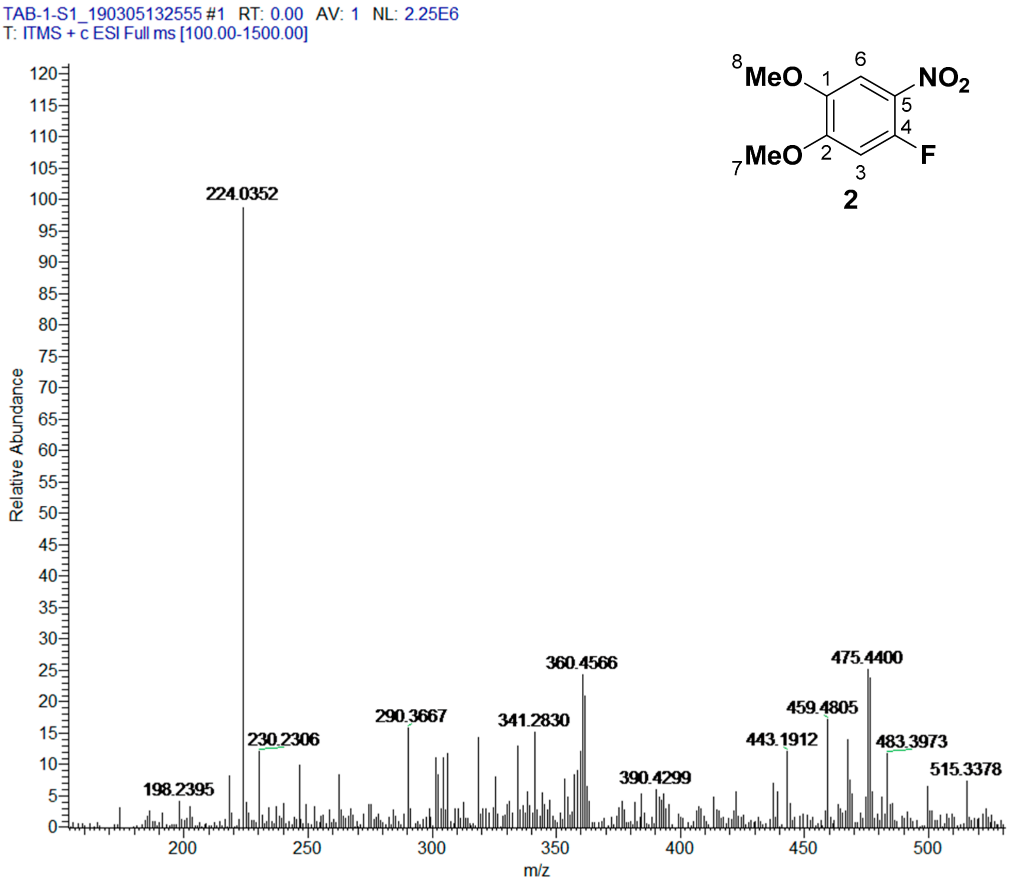

Figure S3. HR-MS of compound 2.

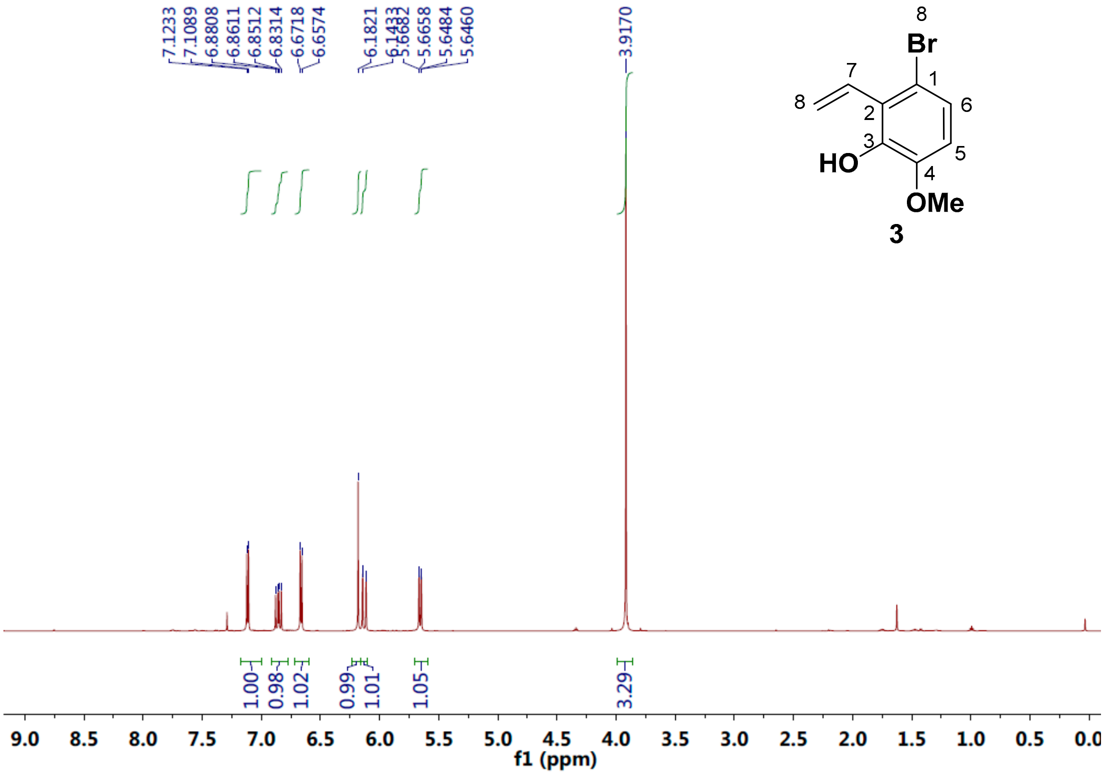

Figure S4. [1] H NMR of compound 3.

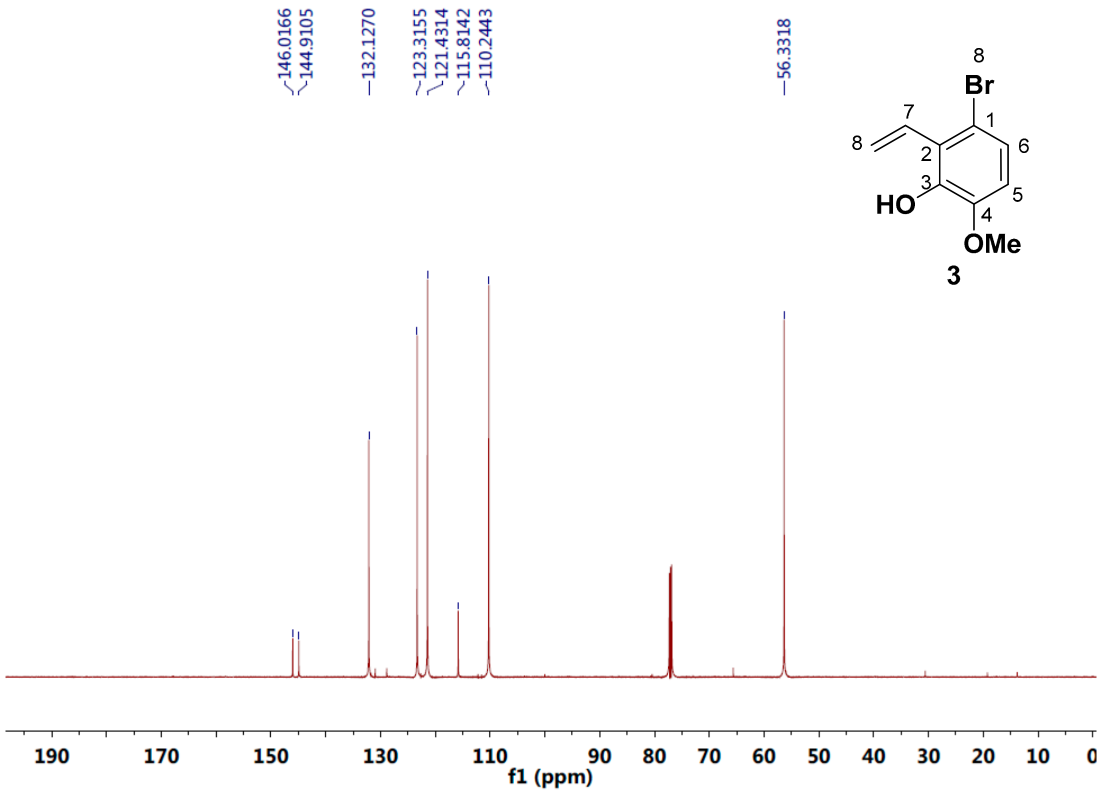

Figure S5. [13] C NMR of compound 3.

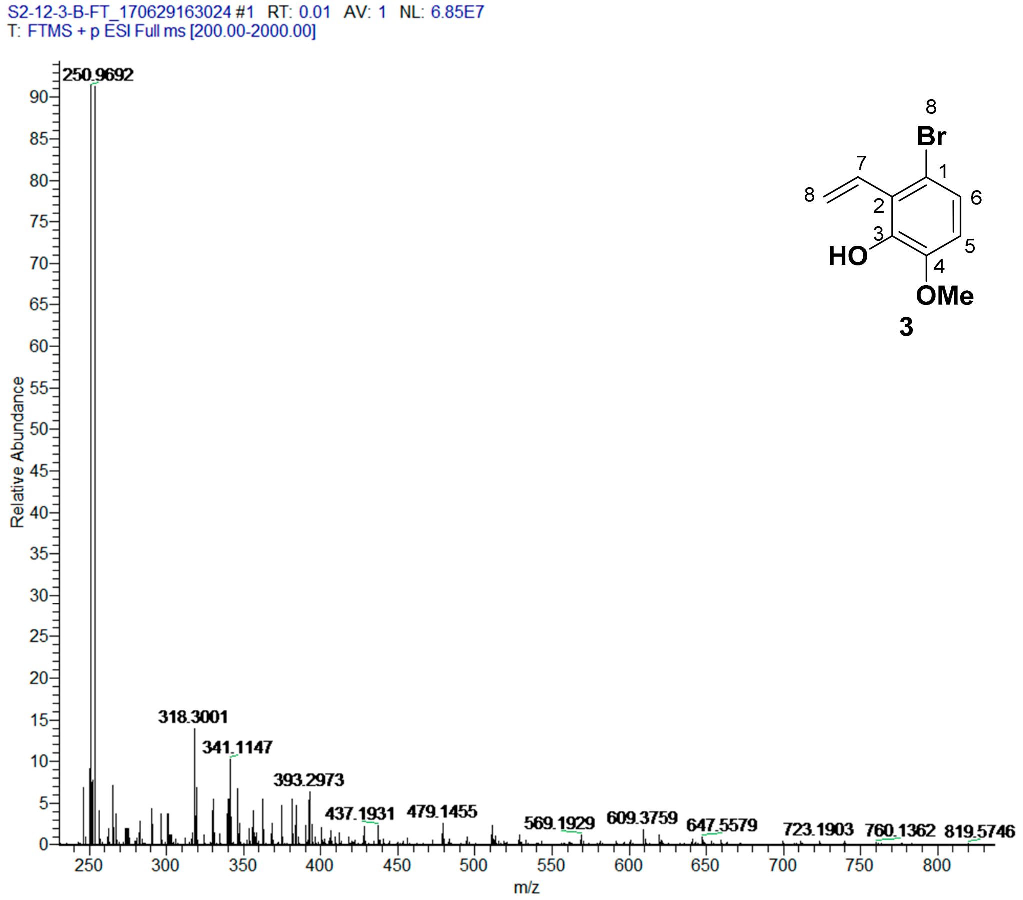

Figure S6. HR-MS of compound 3.

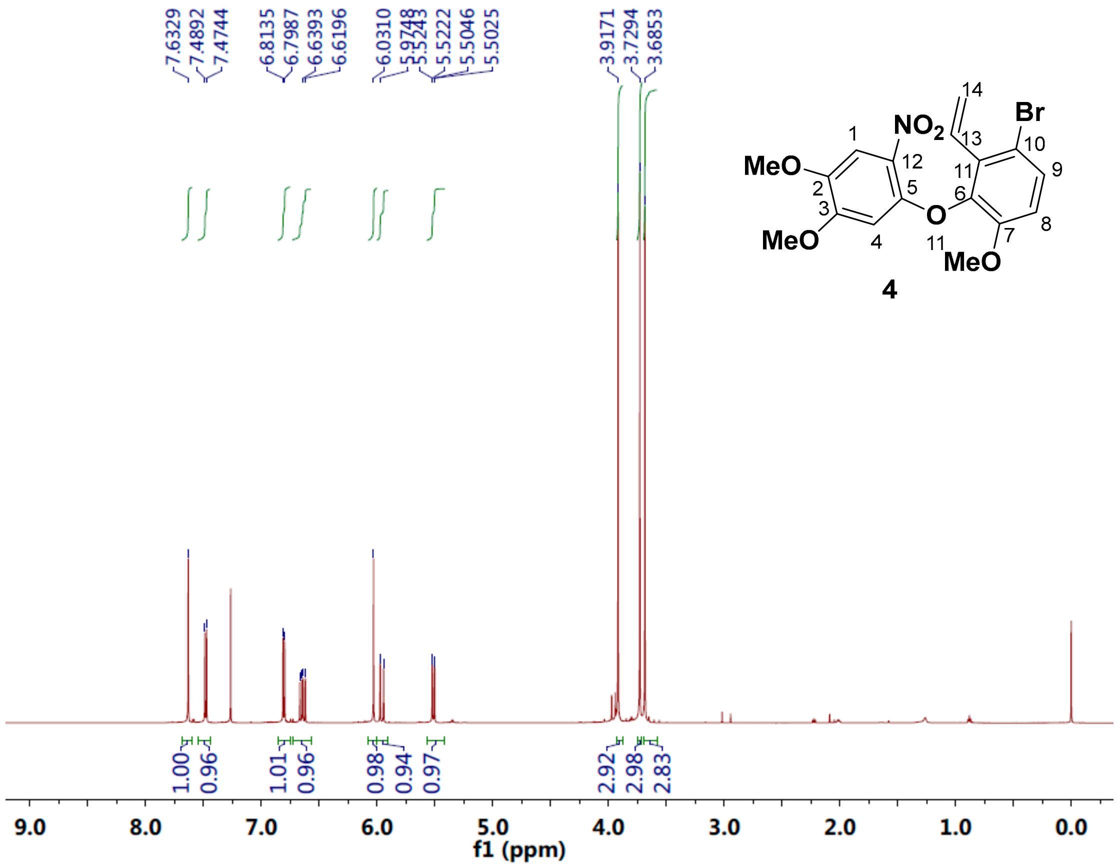

Figure S7. [1] H NMR of compound 4.

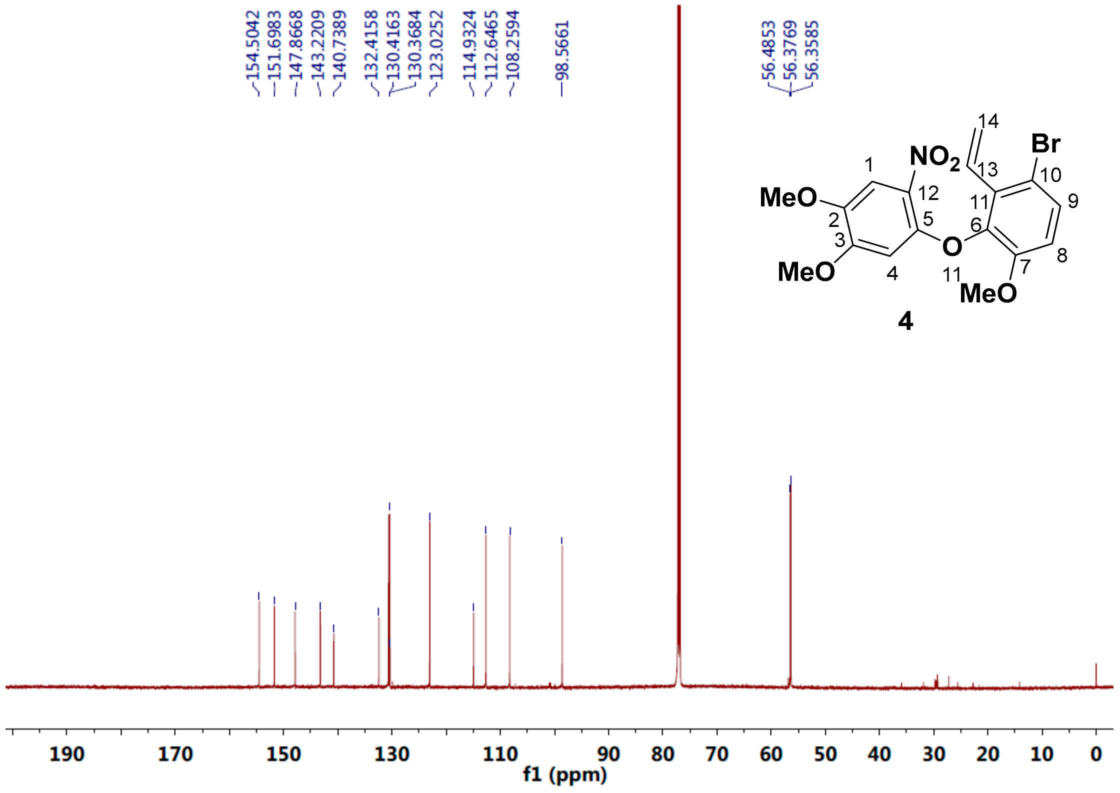

Figure S8. [13] C NMR of compound 4.

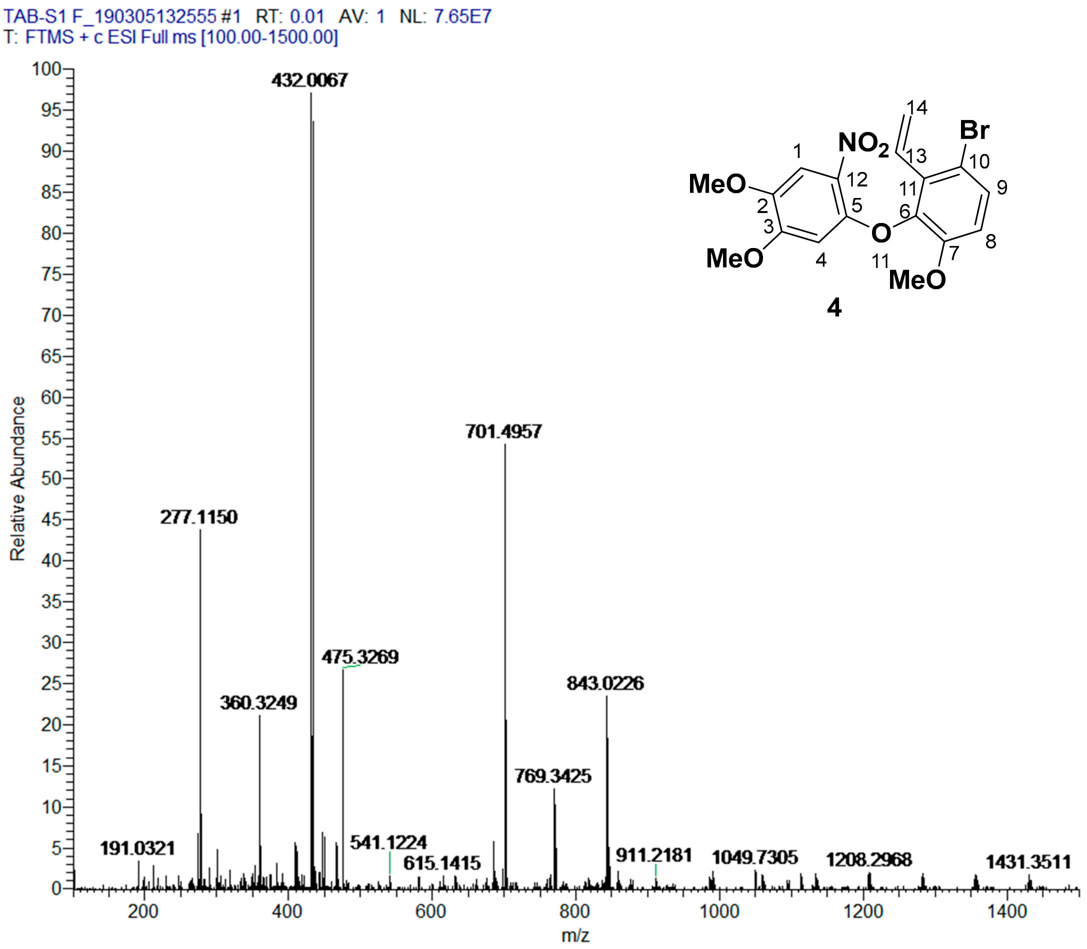

Figure S9. HR-MS of compound 4.

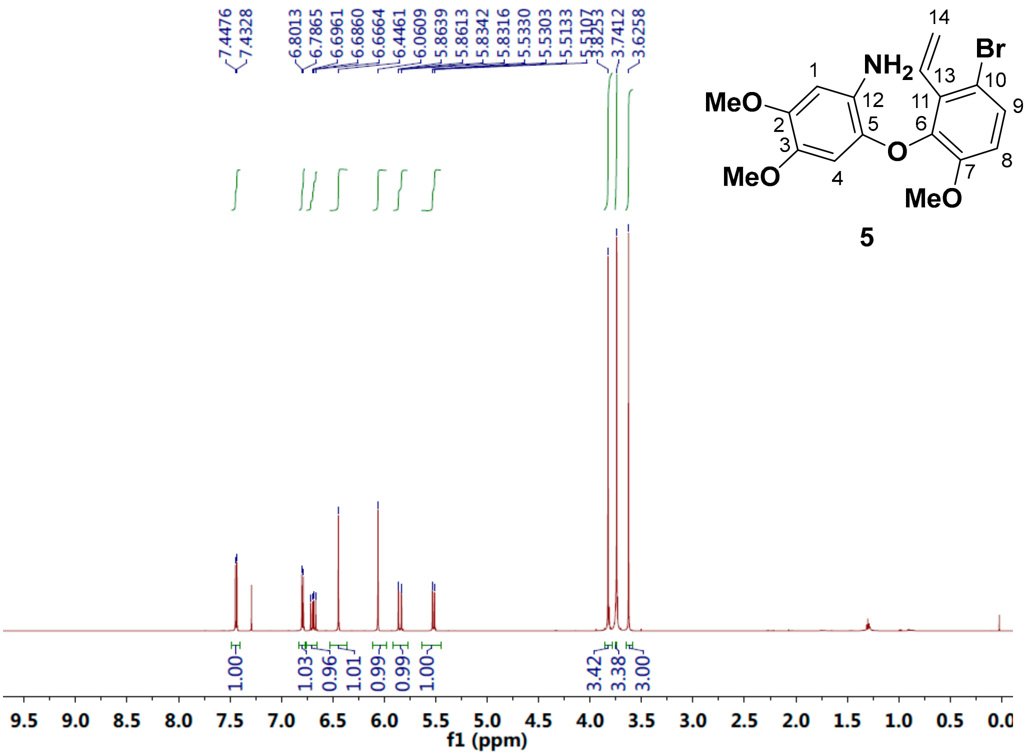

Figure S10. [1] H NMR of compound 5.

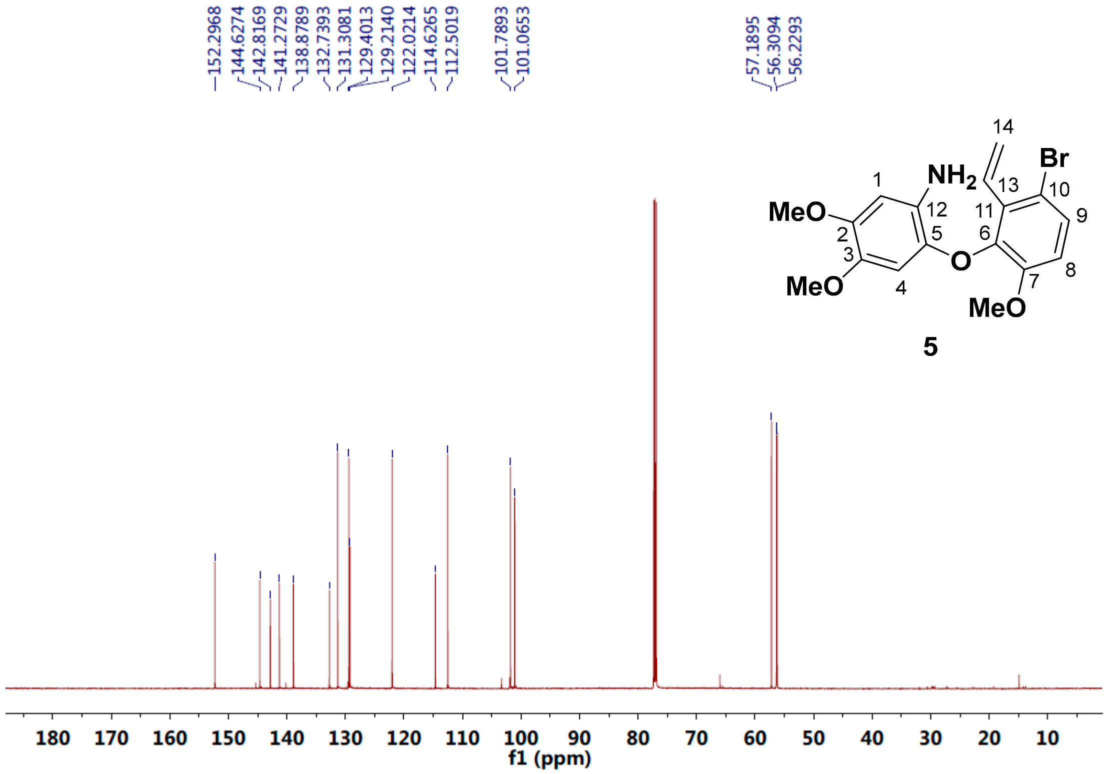

Figure S11. [13] C NMR of compound 5.

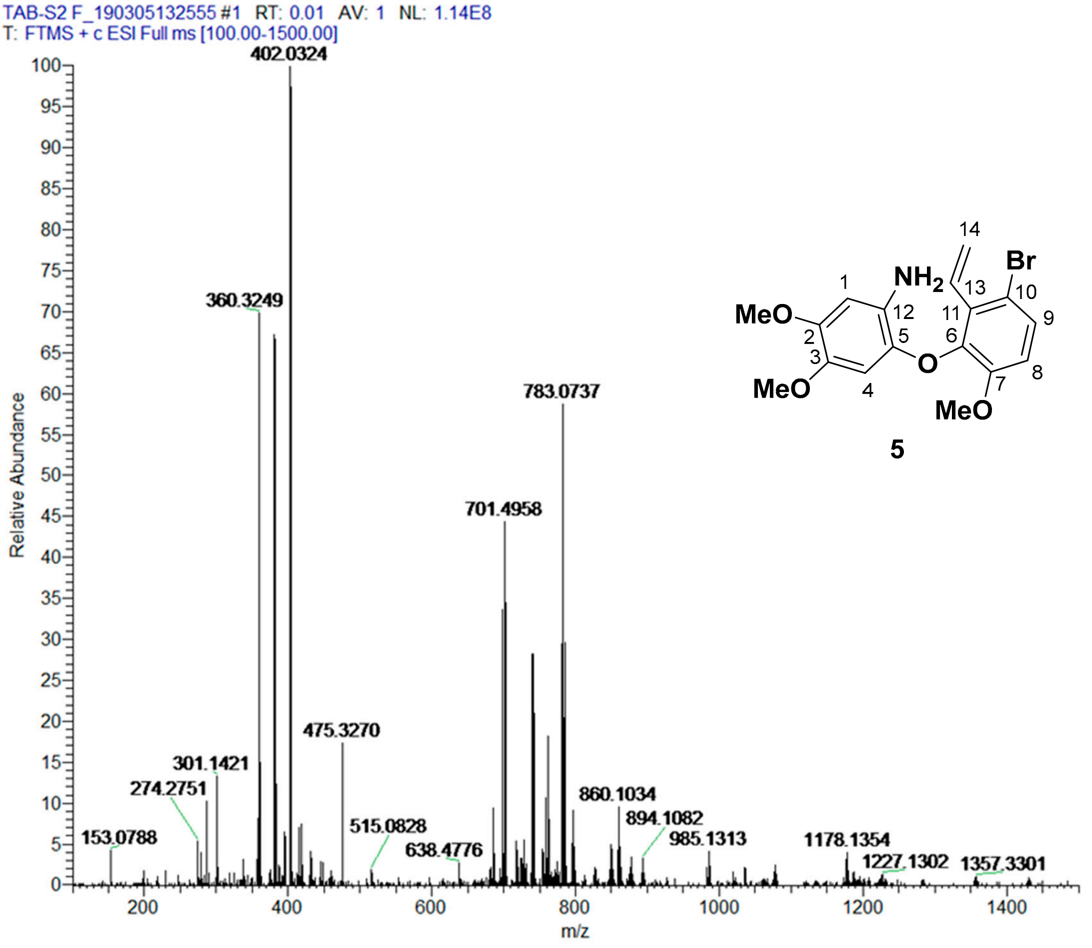

Figure S12. HR-MS of compound 5.

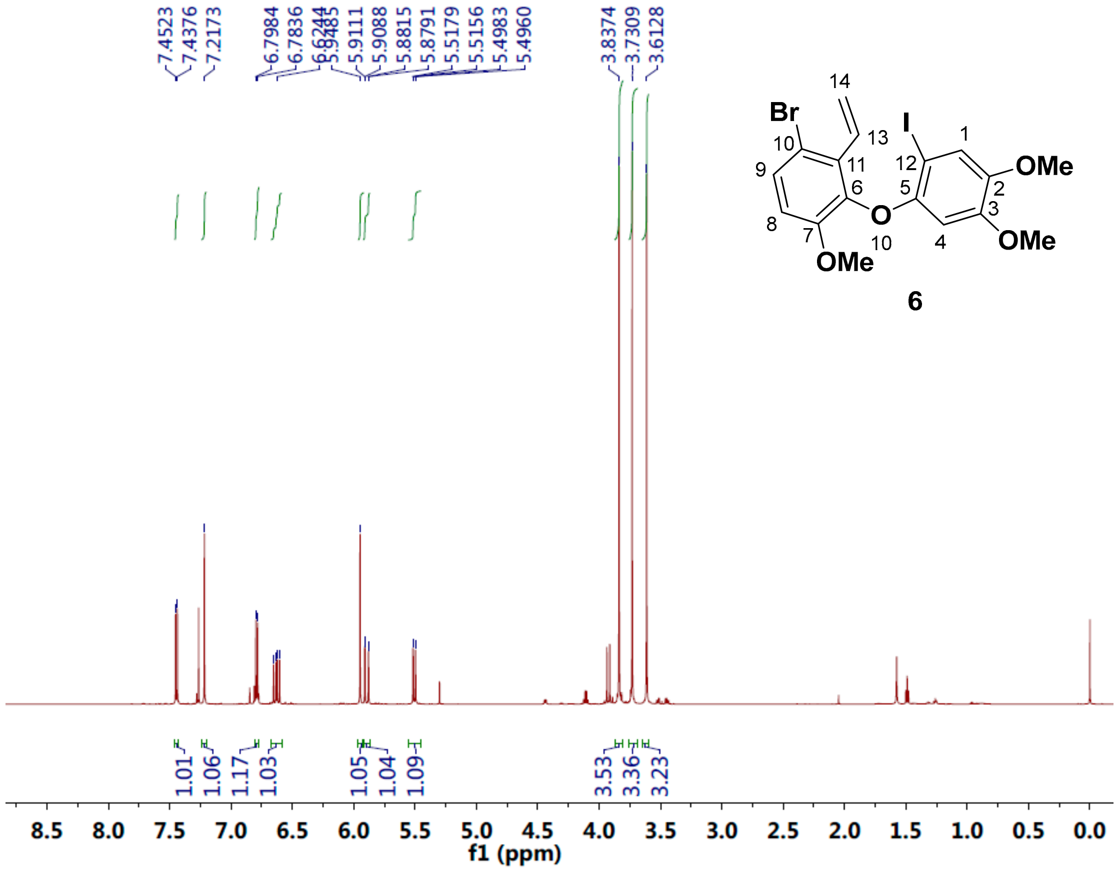

Figure S13. [1] H NMR of compound 6.

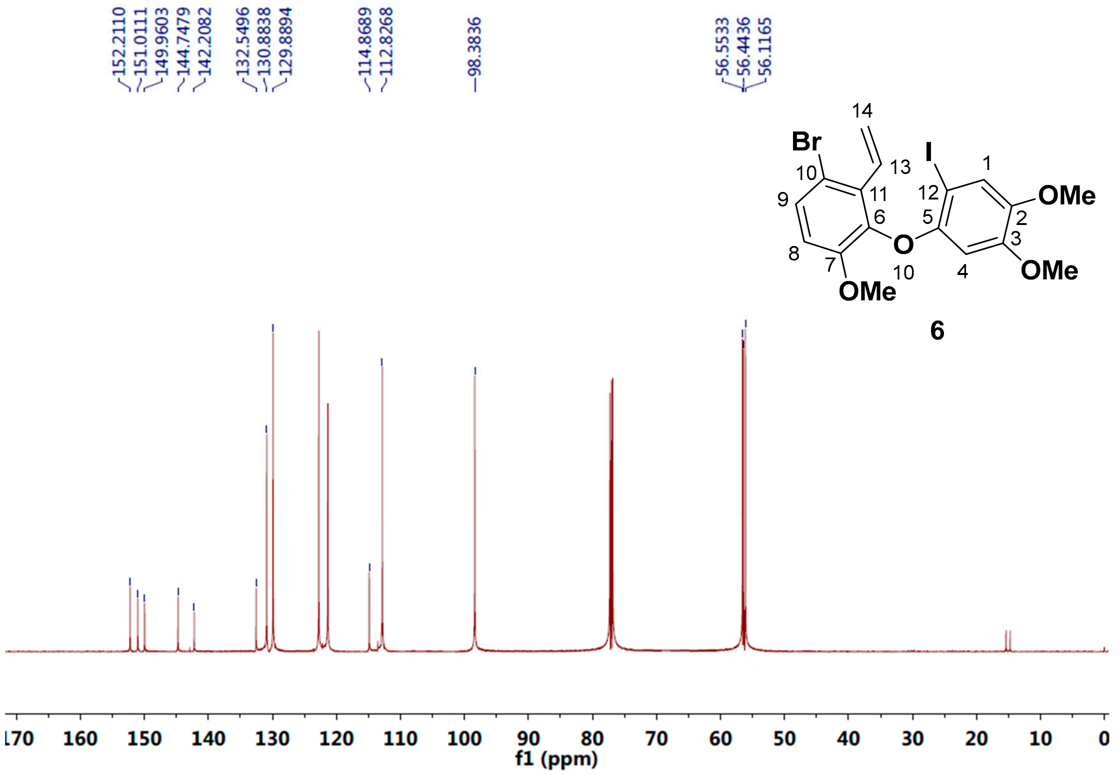

Figure S14. [13] C NMR of compound 6.

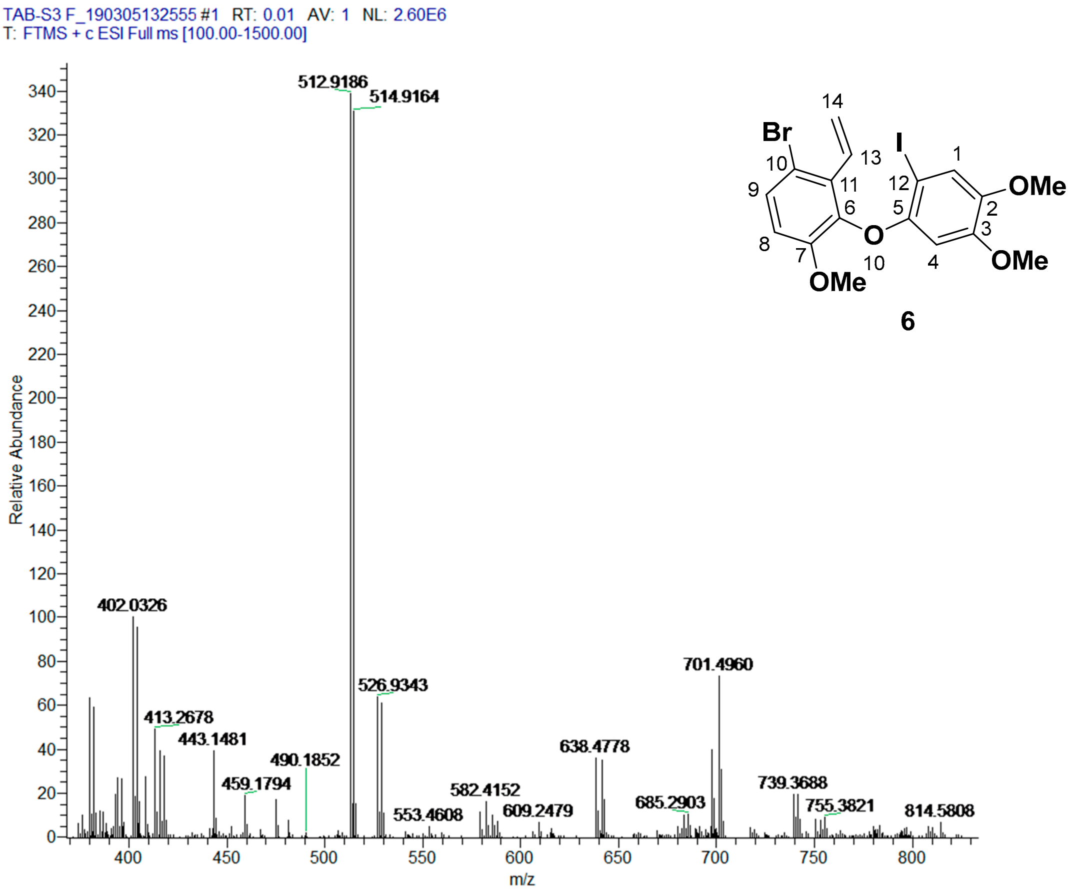

Figure S15. HR-MS of compound 6.

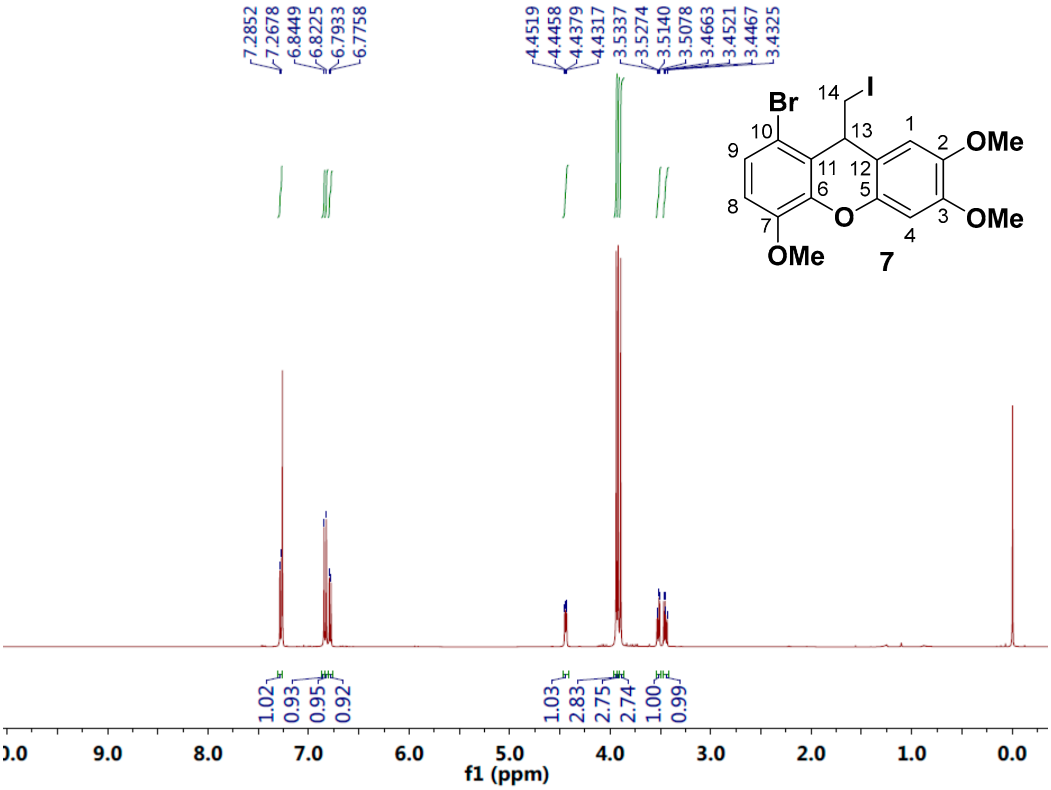

Figure S16. [1] H NMR of compound 7.

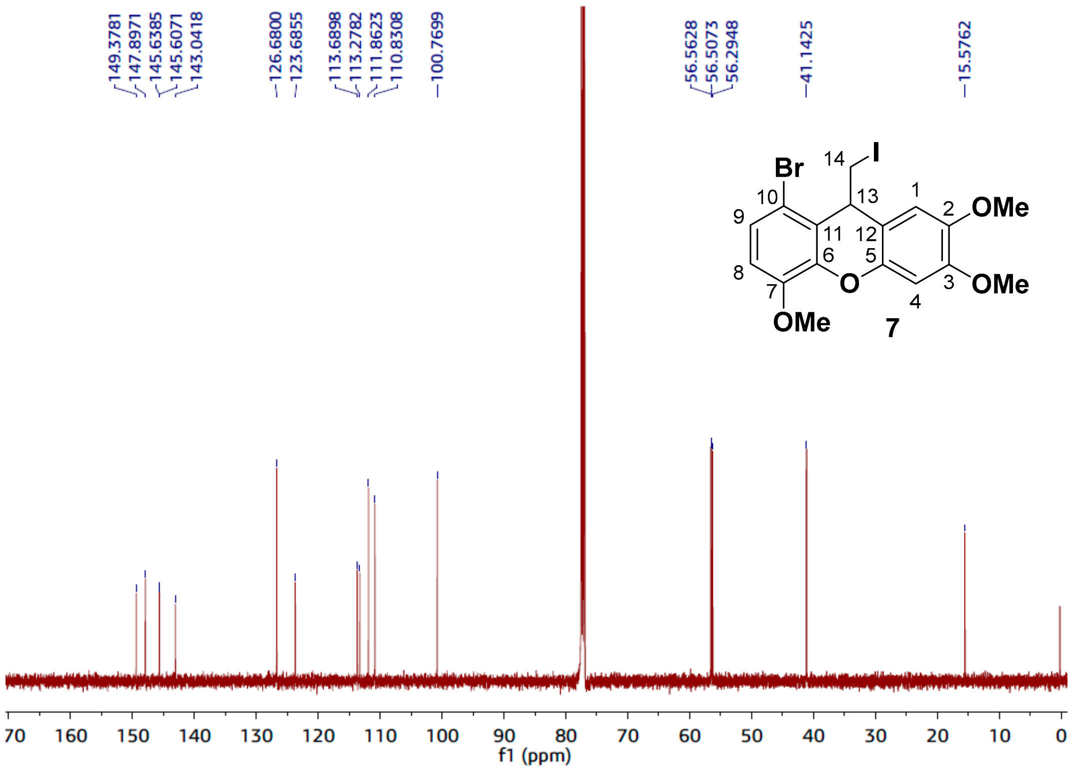

Figure S17. [13] C NMR of compound 7.

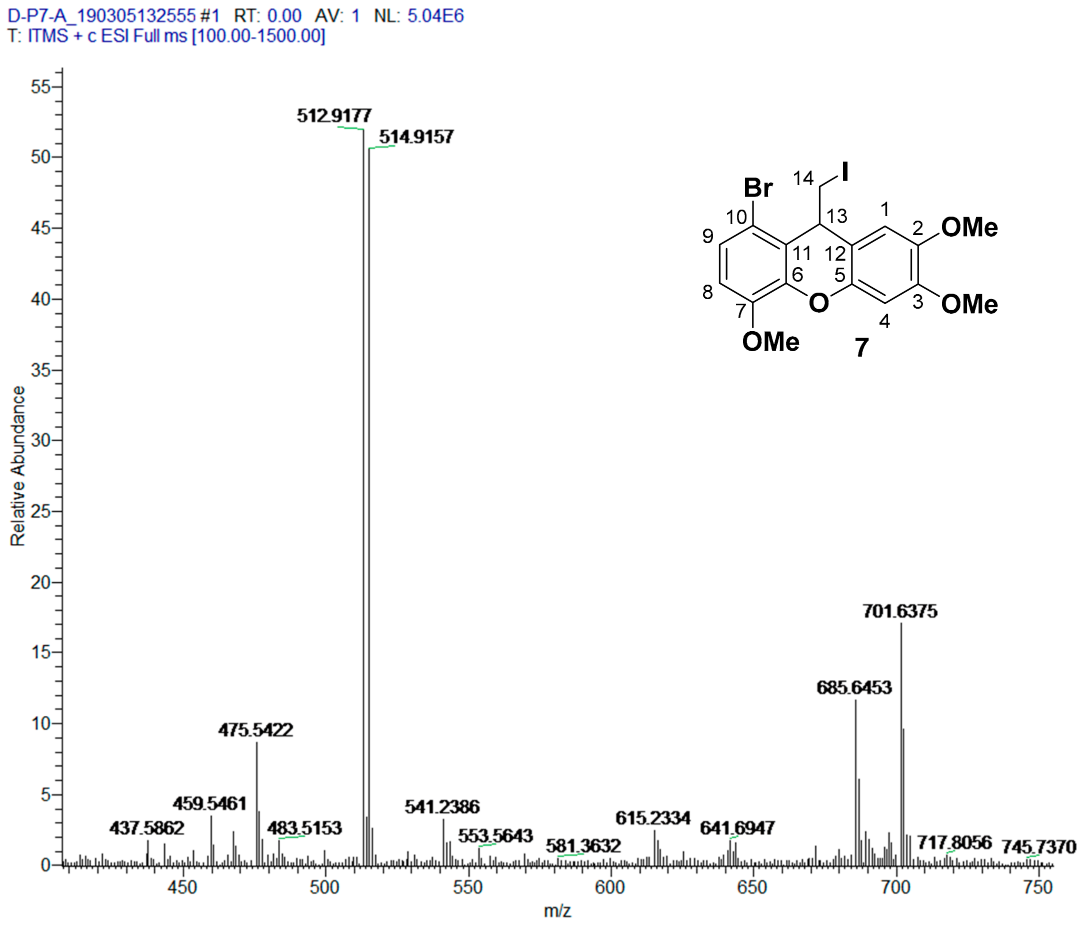

Figure S18. HR-MS of compound 7.

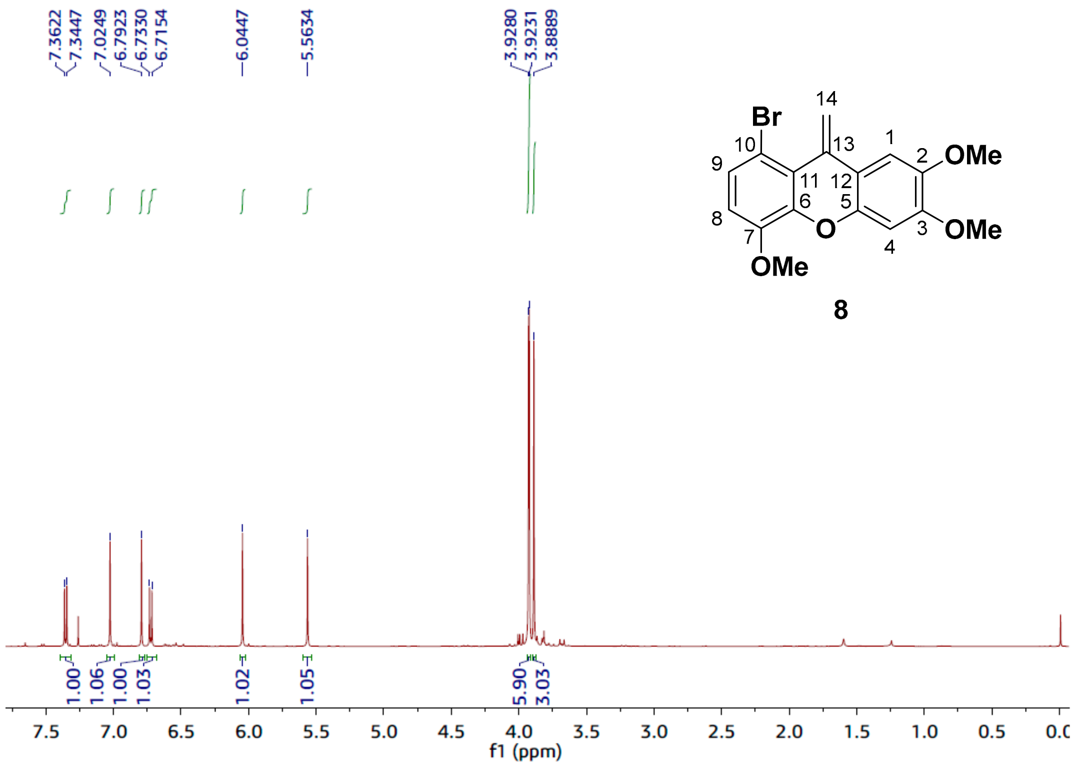

Figure S19. [1] H NMR of compound 8.

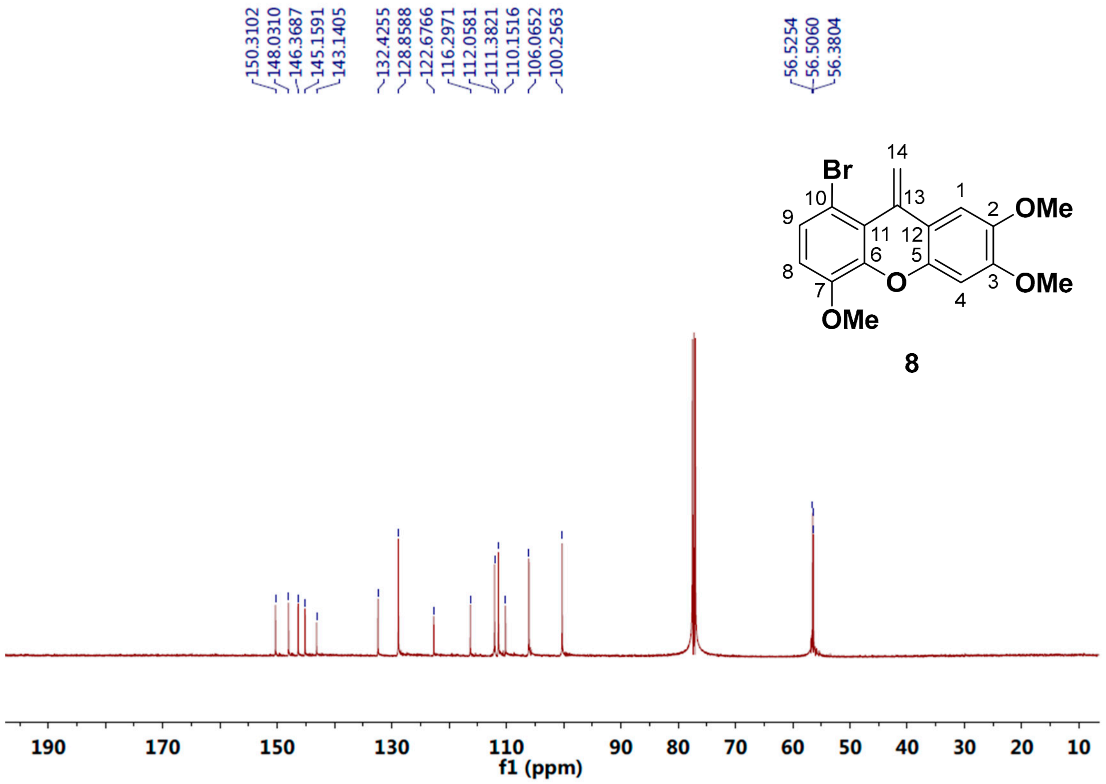

Figure S20. [13] C NMR of compound 8.

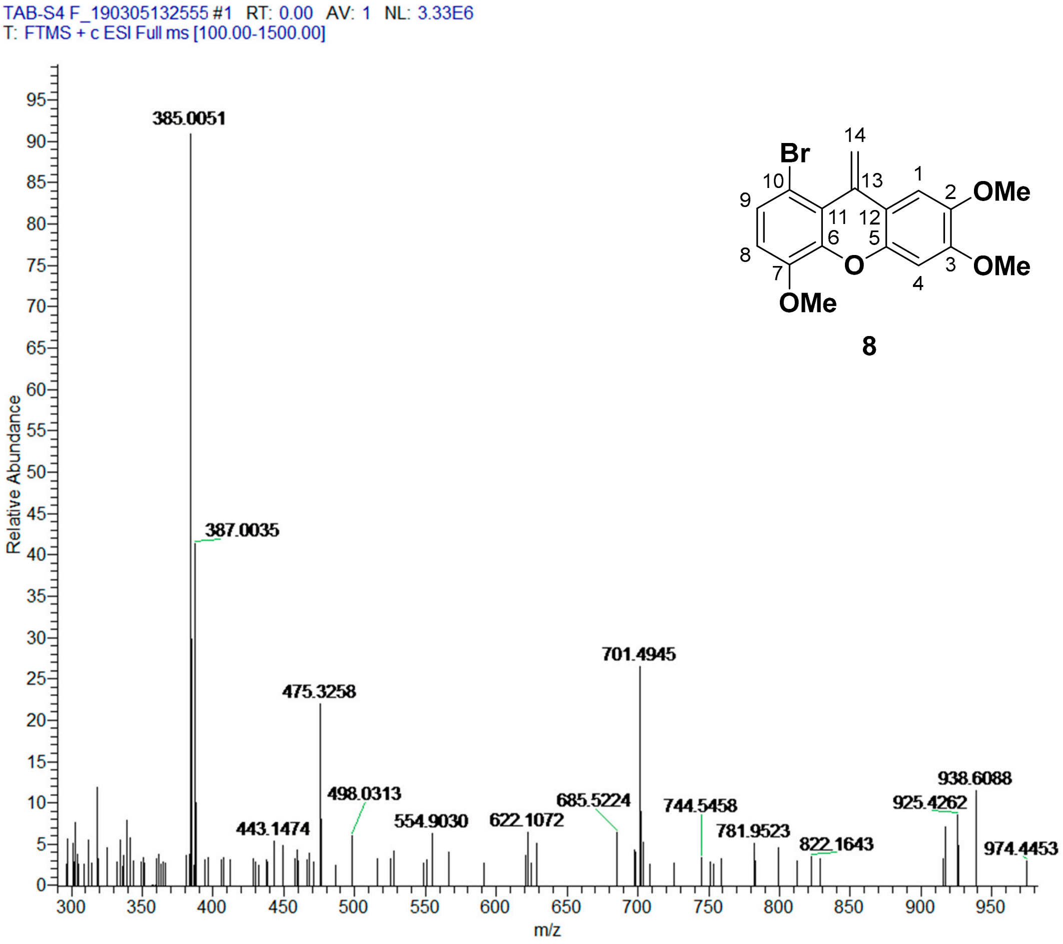

Figure S21. HR-MS of compound 8.

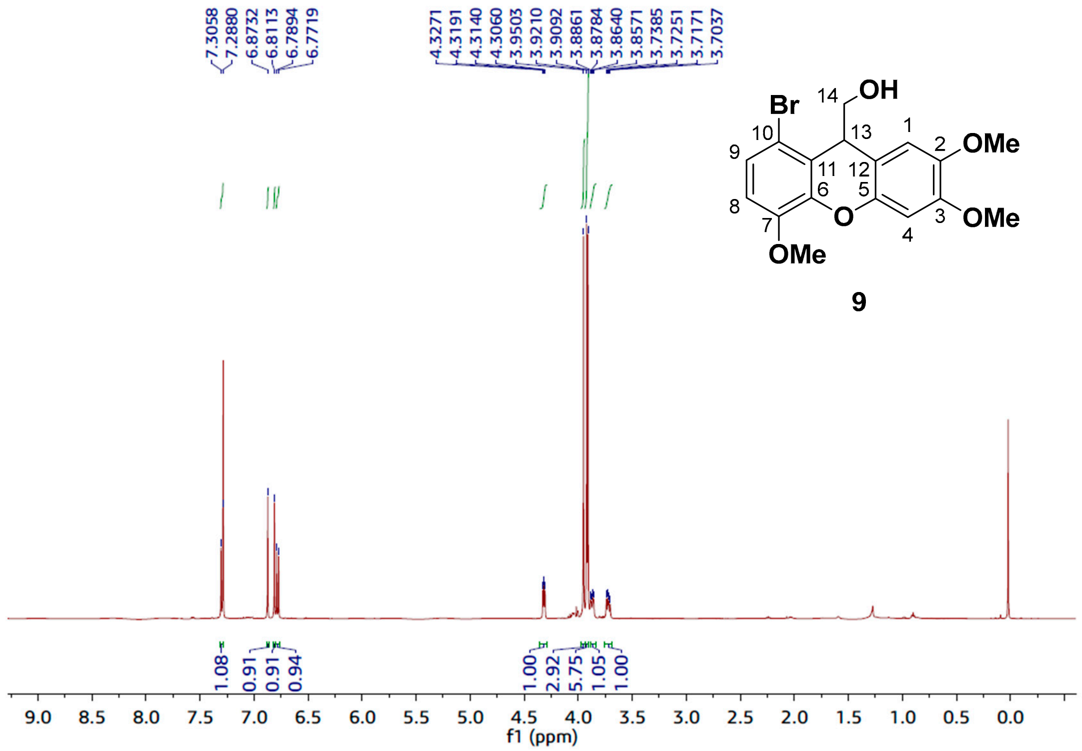

Figure S22. [1] H NMR of compound 9.

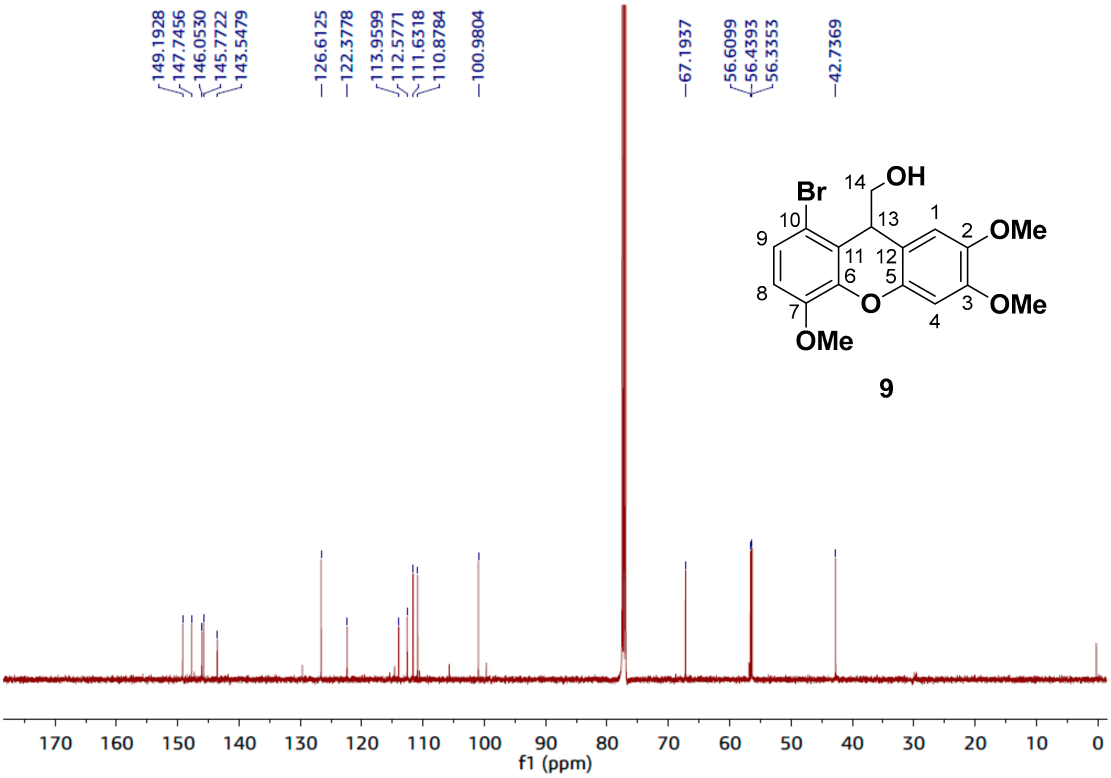

Figure S23. ^{13}C NMR of compound 9.

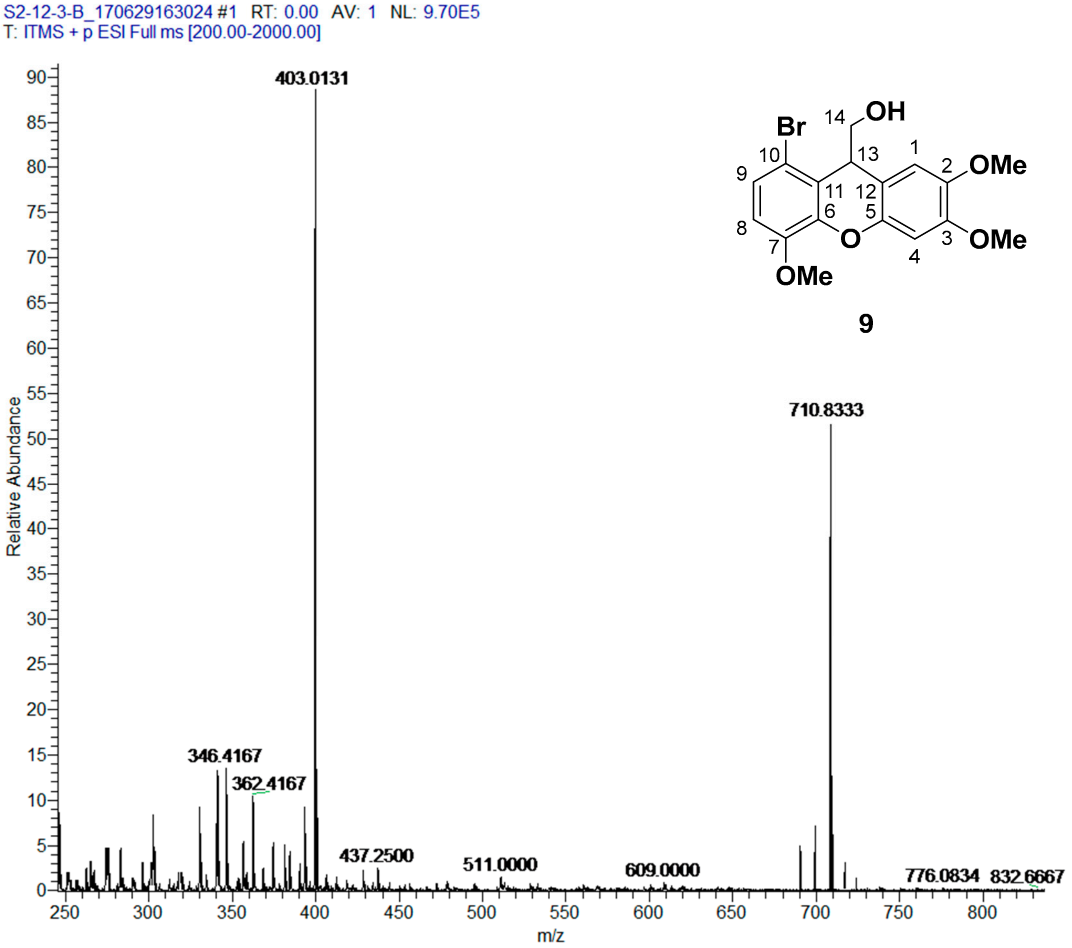

Figure S24. HR-MS of compound 9

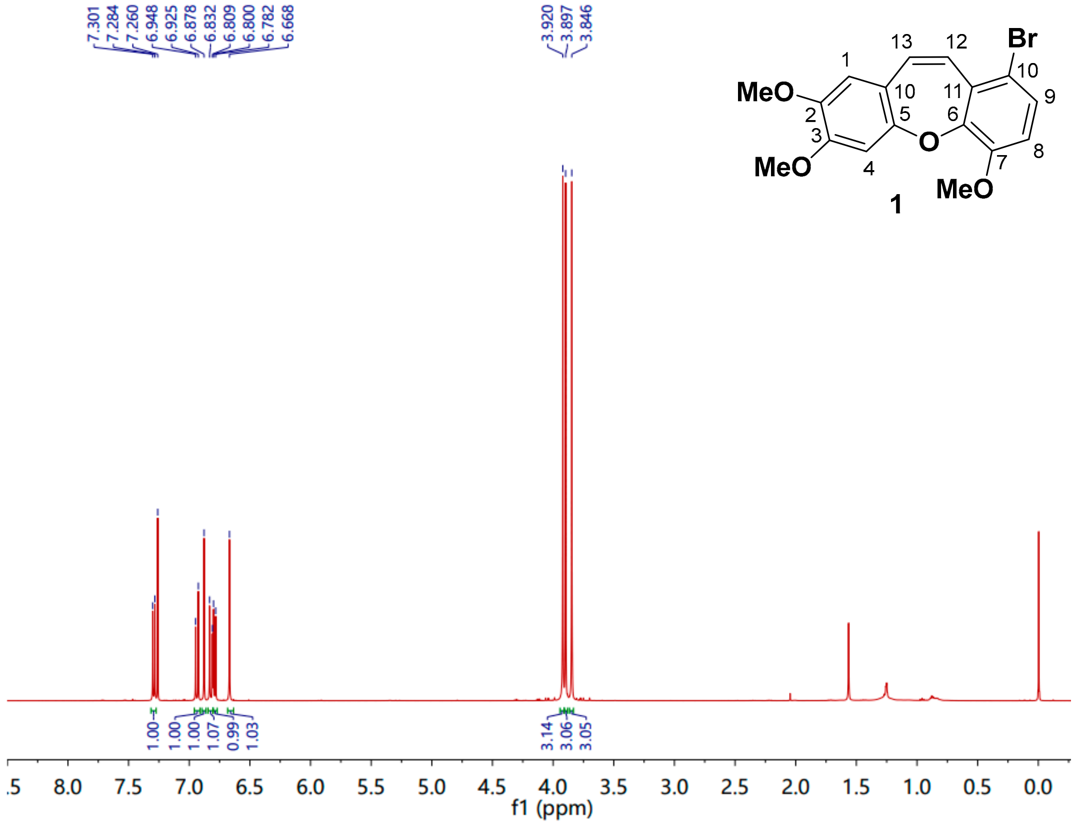

Figure S25. [1] H NMR of compound 1.

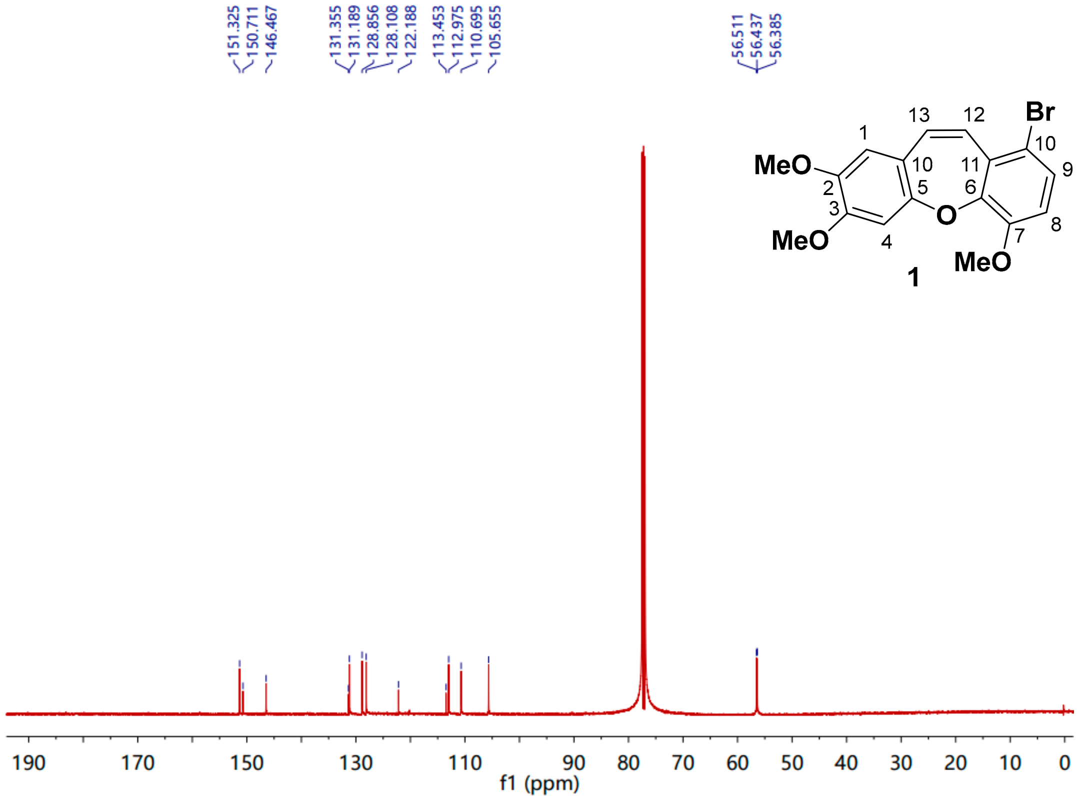

Figure S26. [13] C NMR of compound 1.

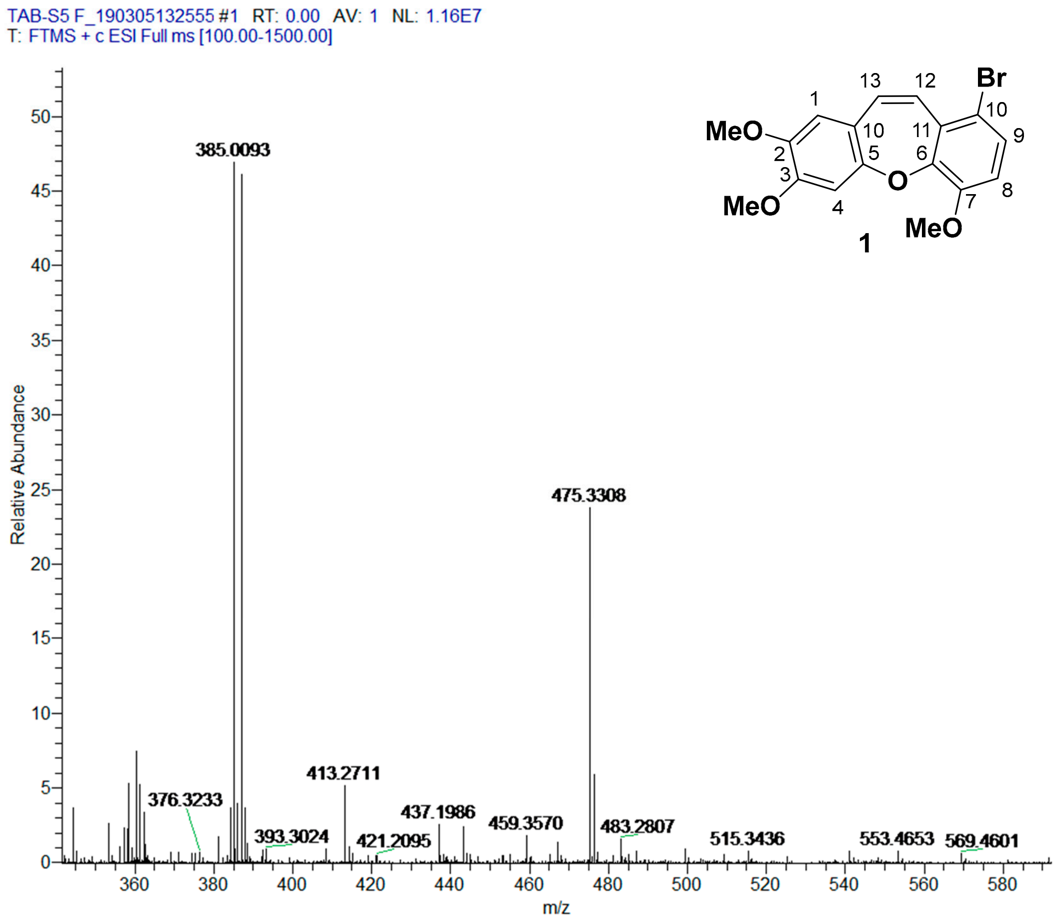

Figure S27. HR-MS of compound 1.

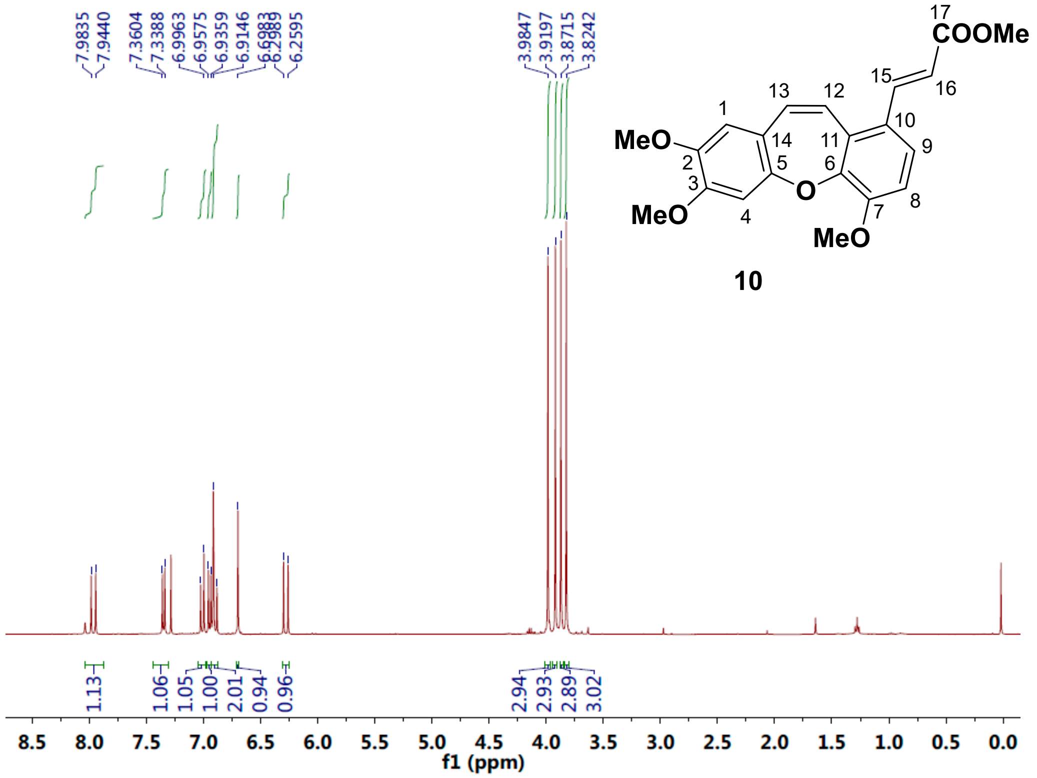

Figure S28. [1] H NMR of compound 10.

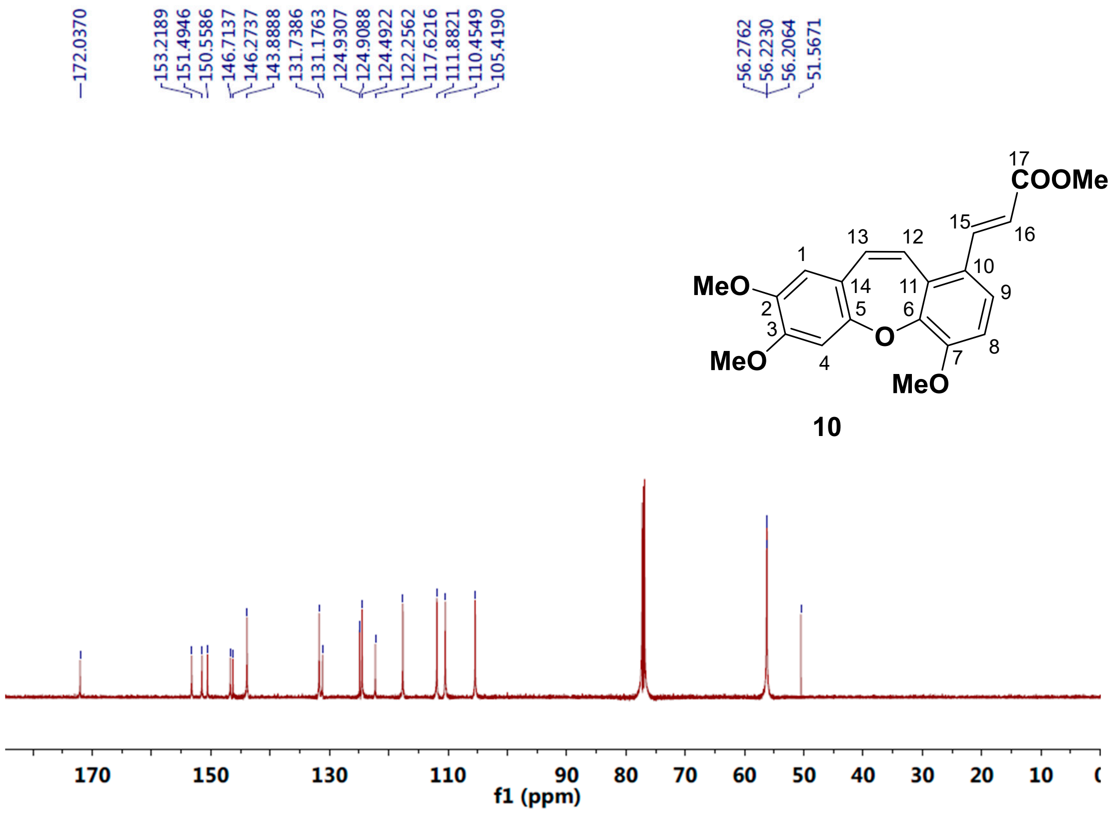

Figure S29. [13] C NMR of compound 10.

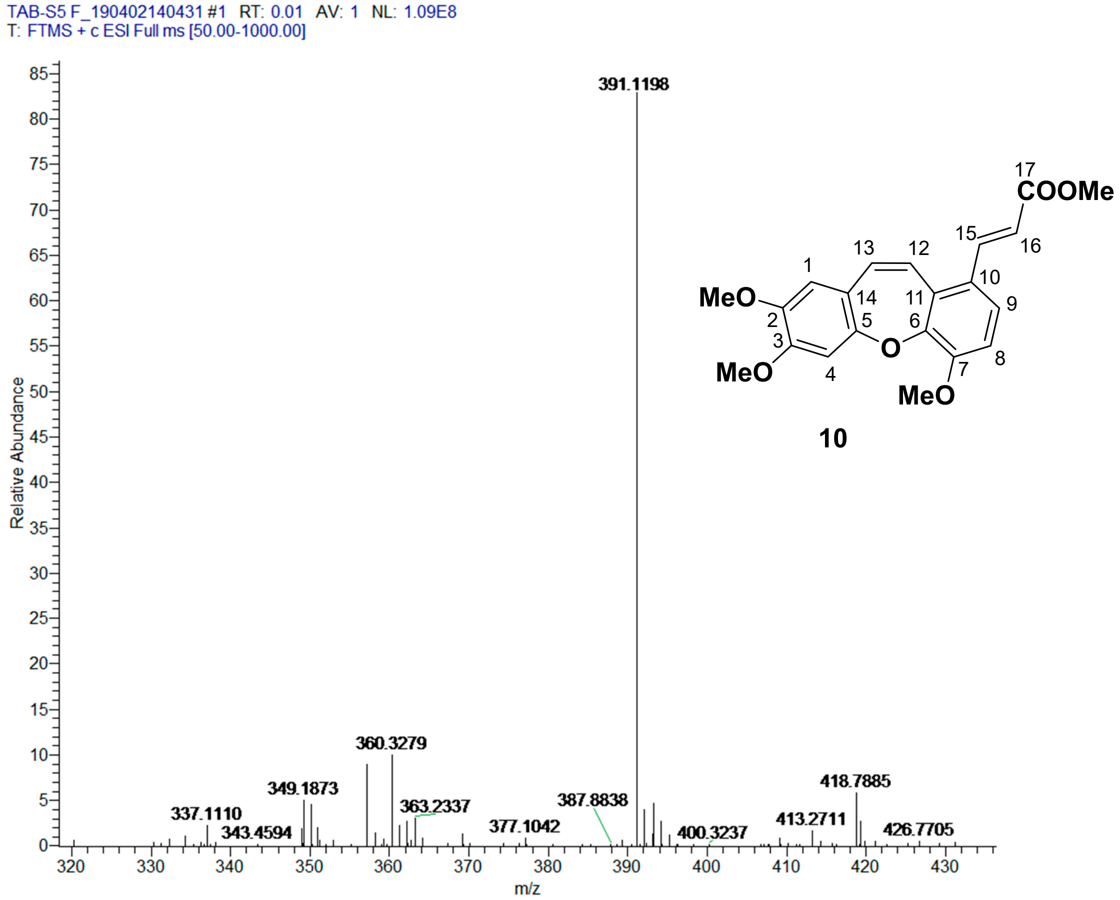

Figure S30. HR-MS of compound 10.

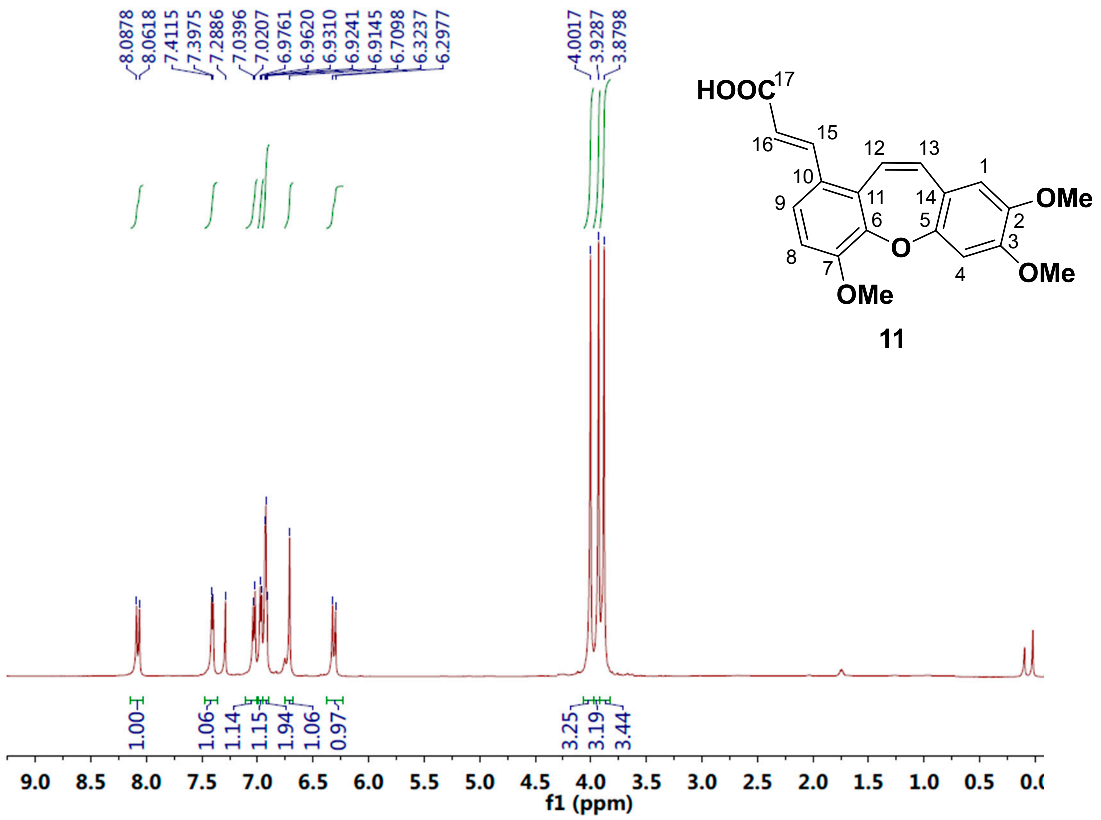

Figure S31. [1] H NMR of compound 11.

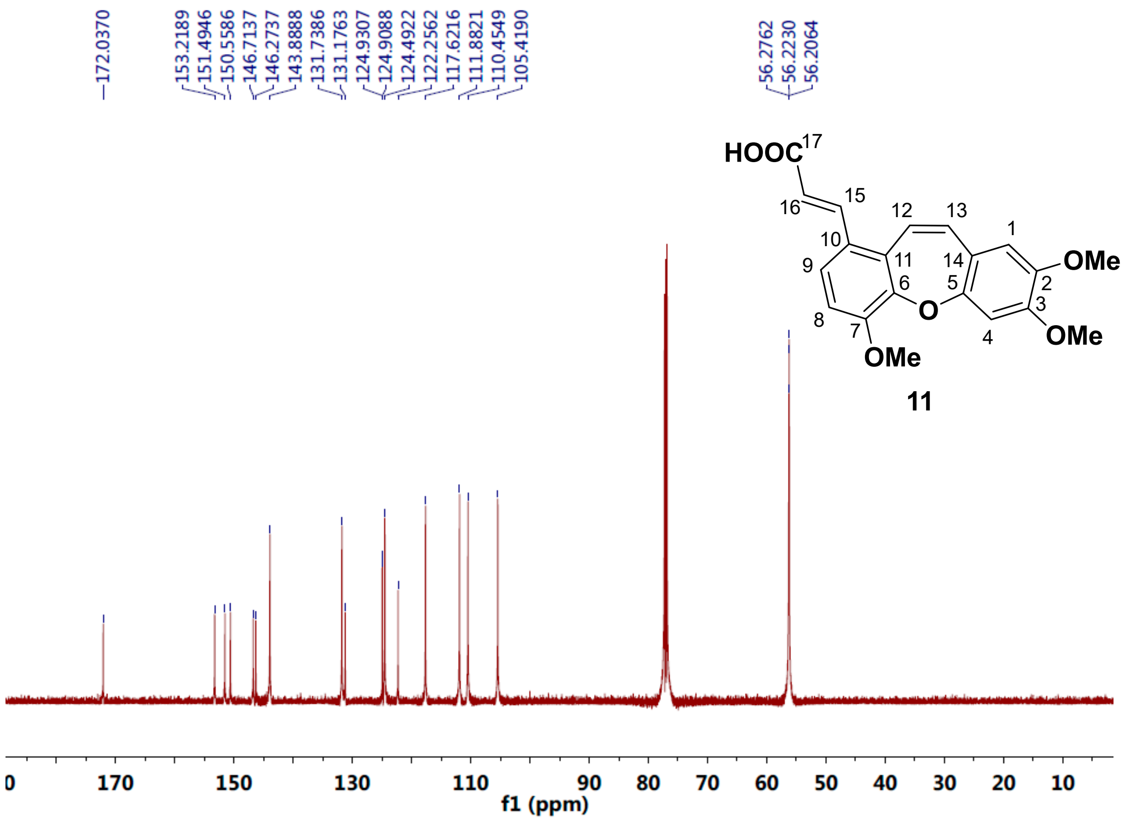

Figure S32. [13] C NMR of compound 11.

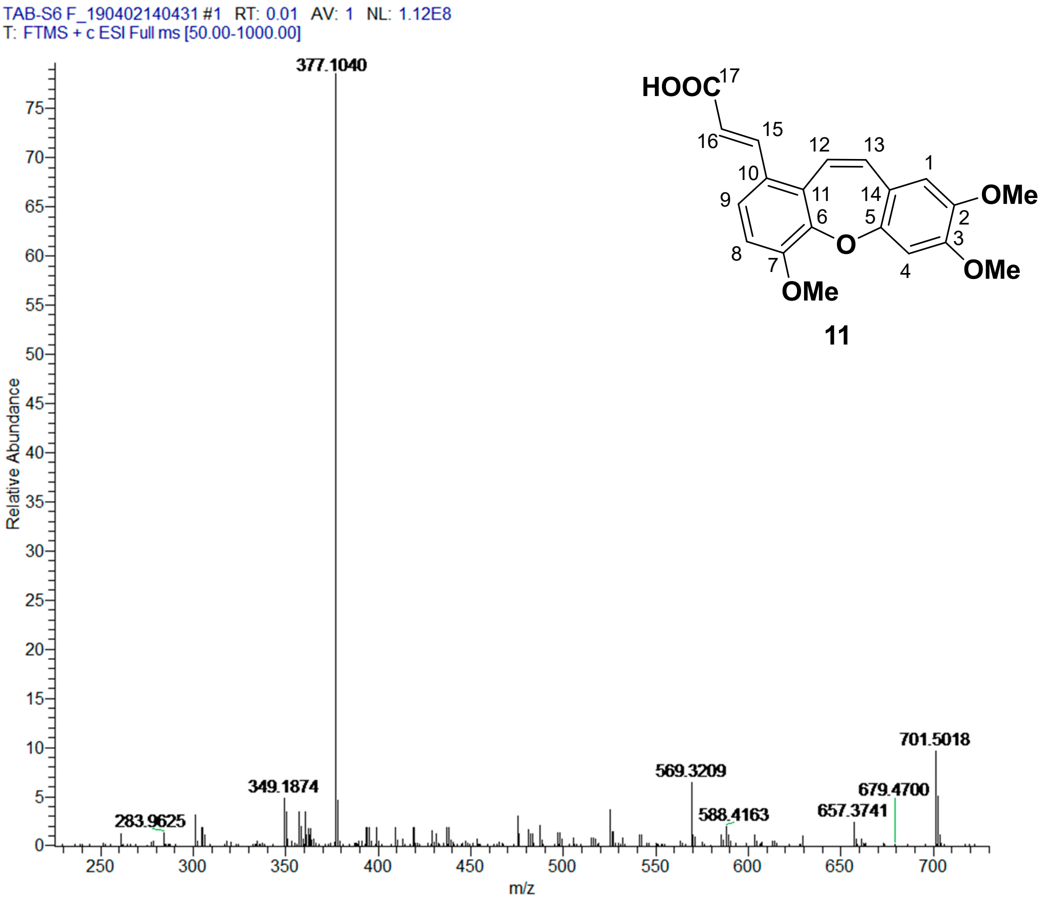

Figure S33. HR-MS of compound 11.

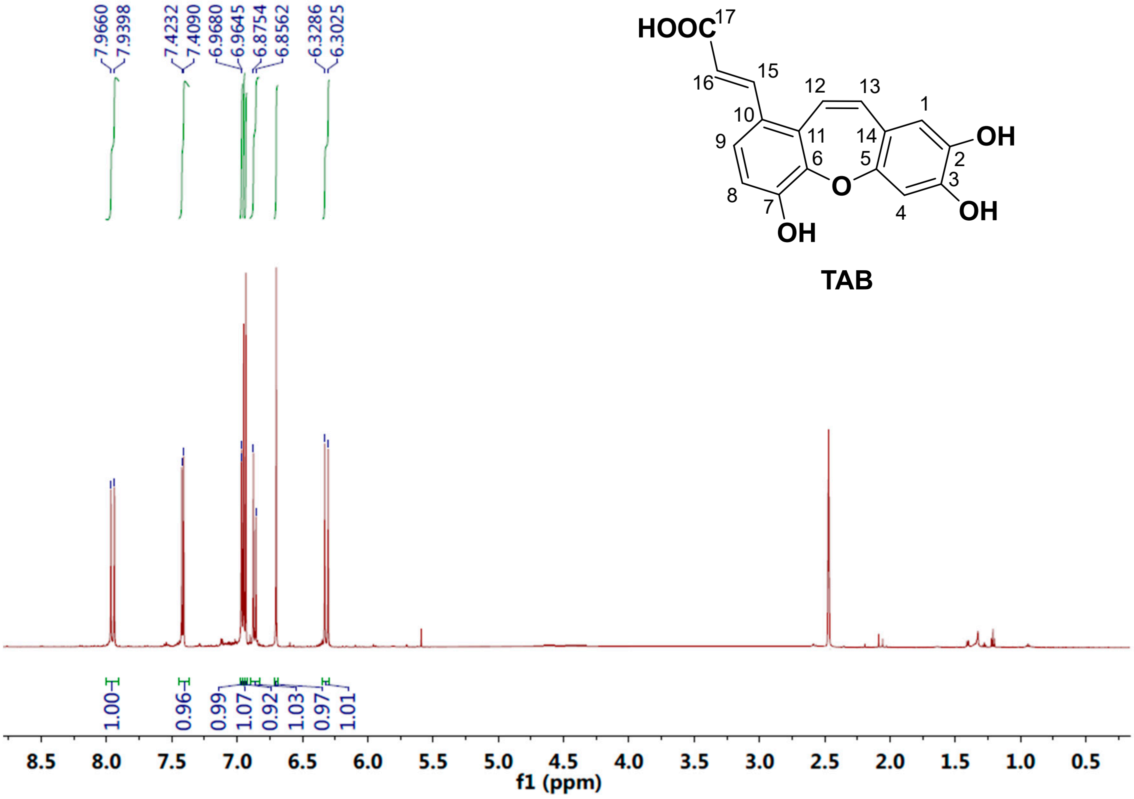

Figure S34. ^{1}H NMR of compound TAB.

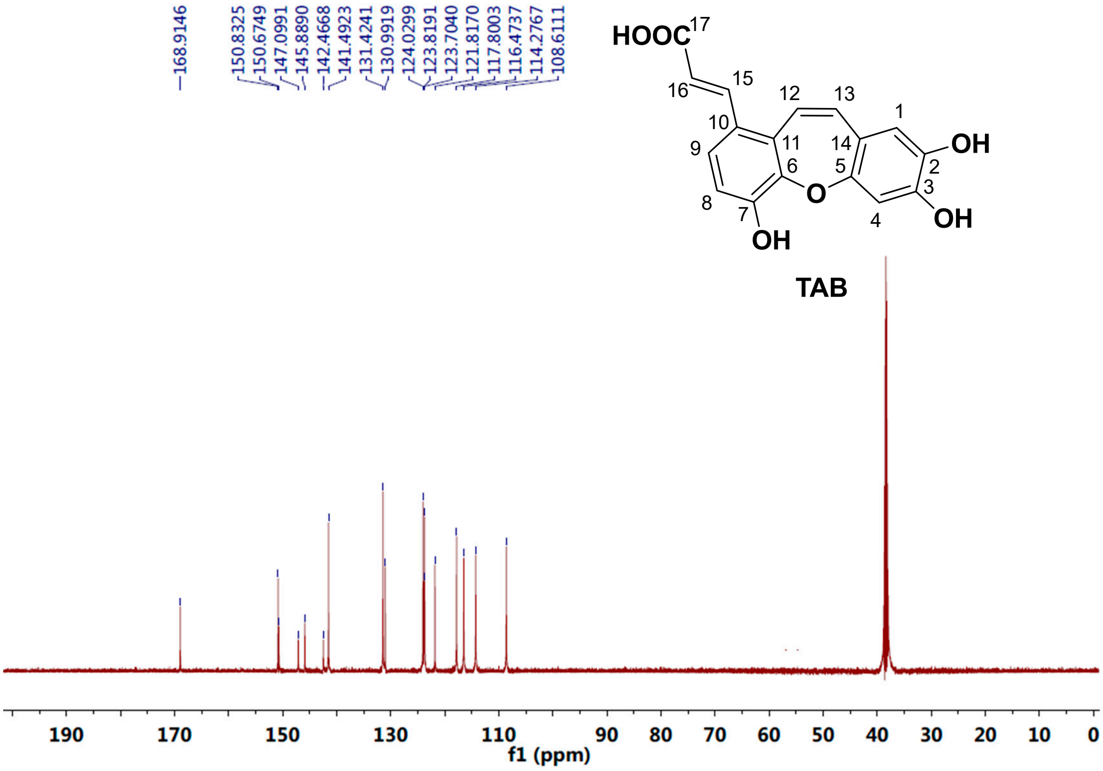

Figure S35. [13] C NMR of compound TAB.

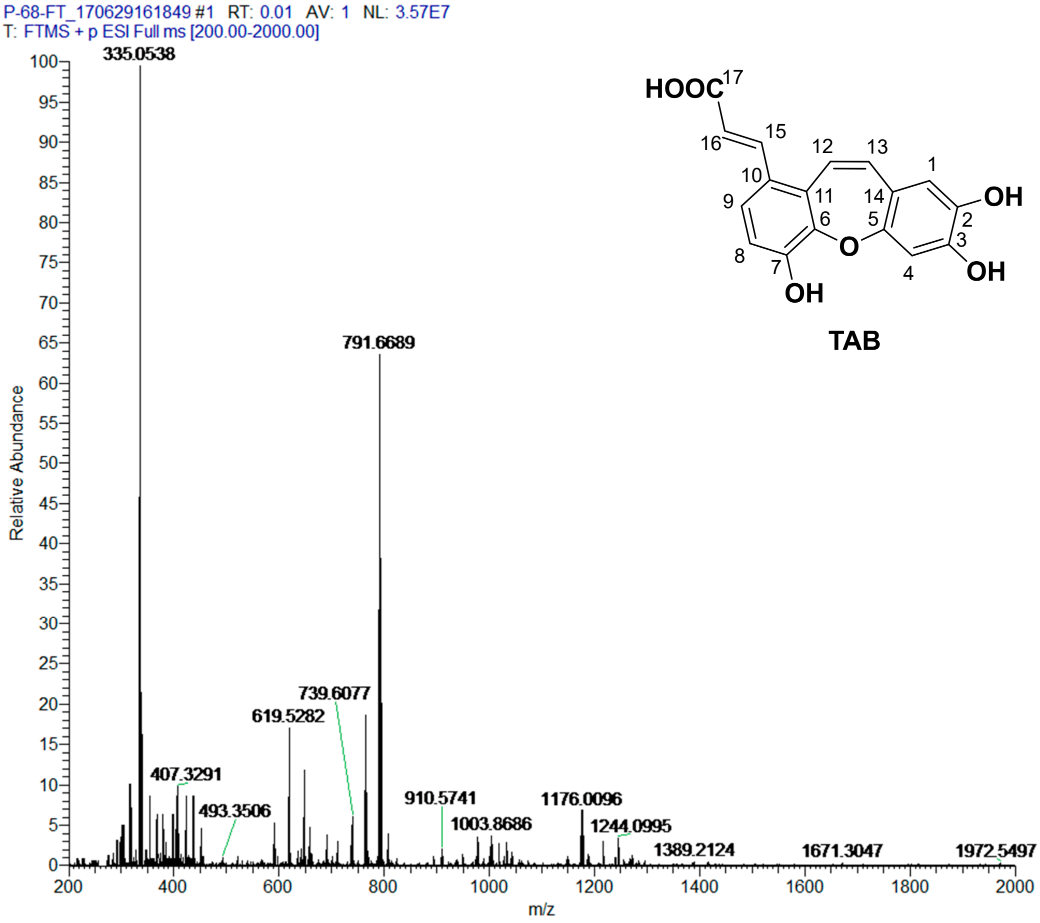

Figure S36. HR-MS of compound TAB.

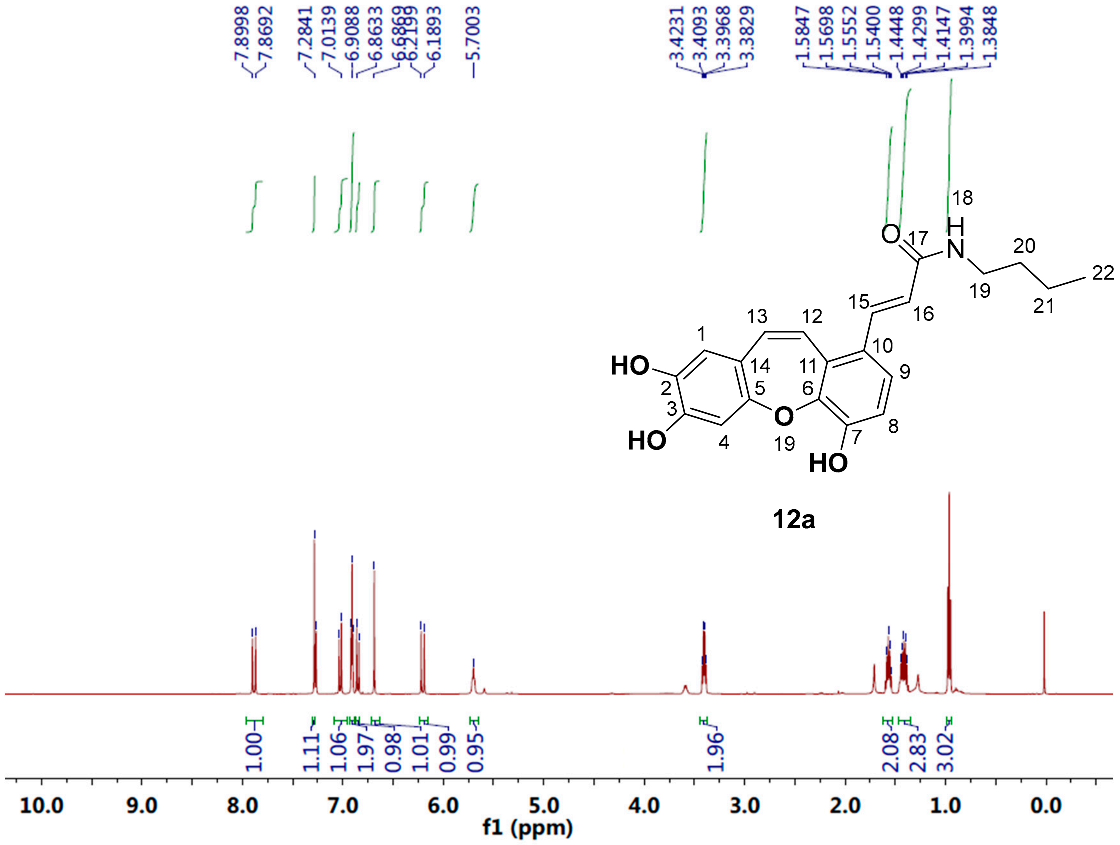

Figure S37. [1] H NMR of compound 12a.

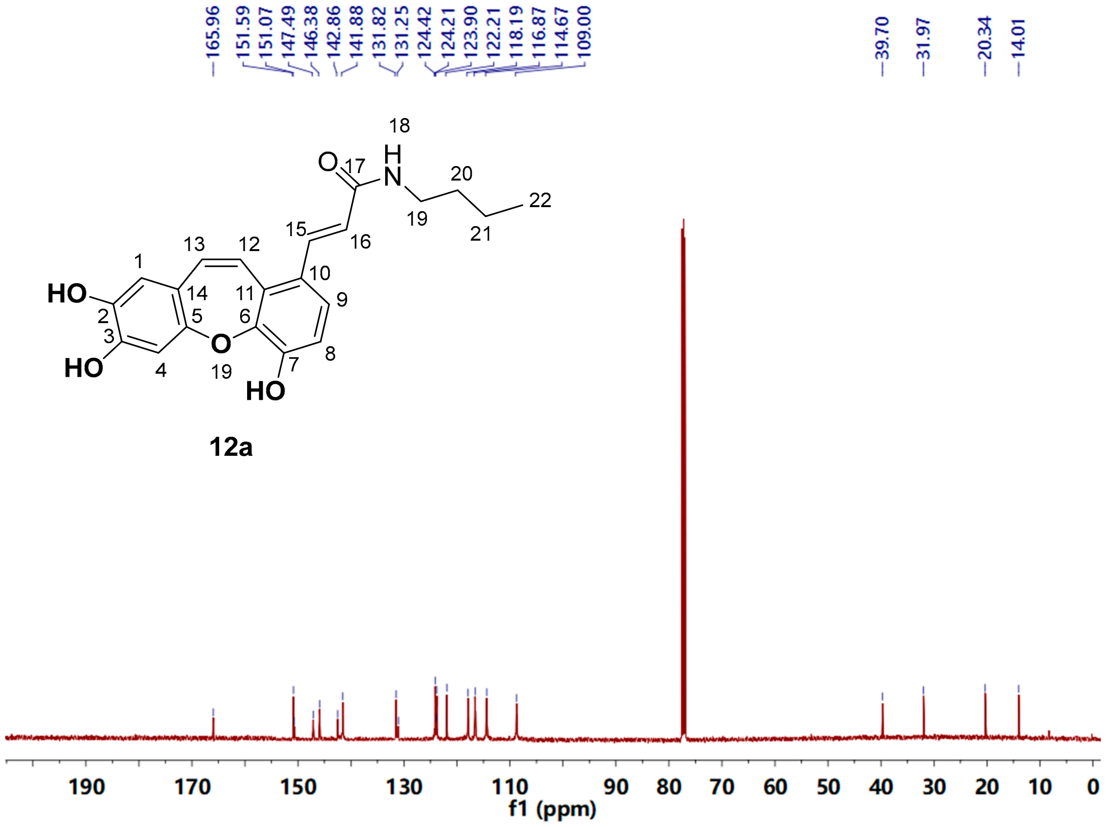

Figure S38. [13] C NMR of compound 12a

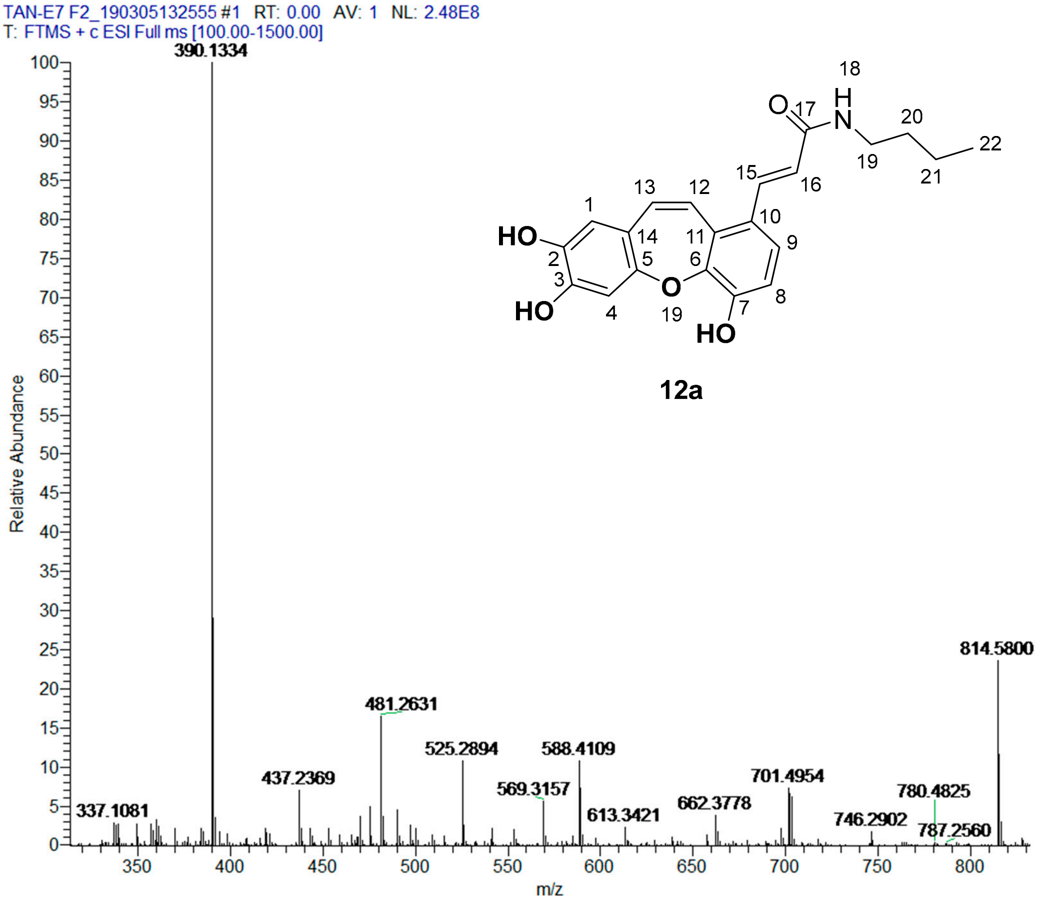

Figure S39. HR-MS of compound 12a.

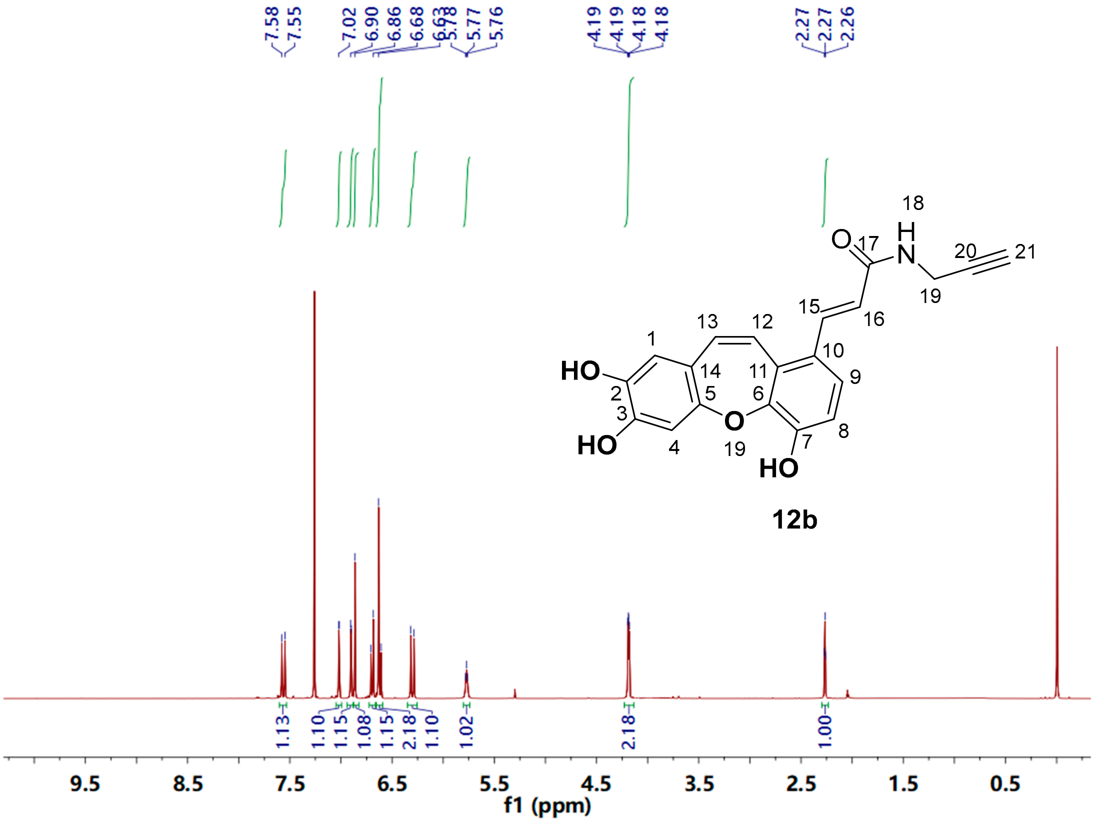

Figure S40. ^{1}H NMR of compound 12b.

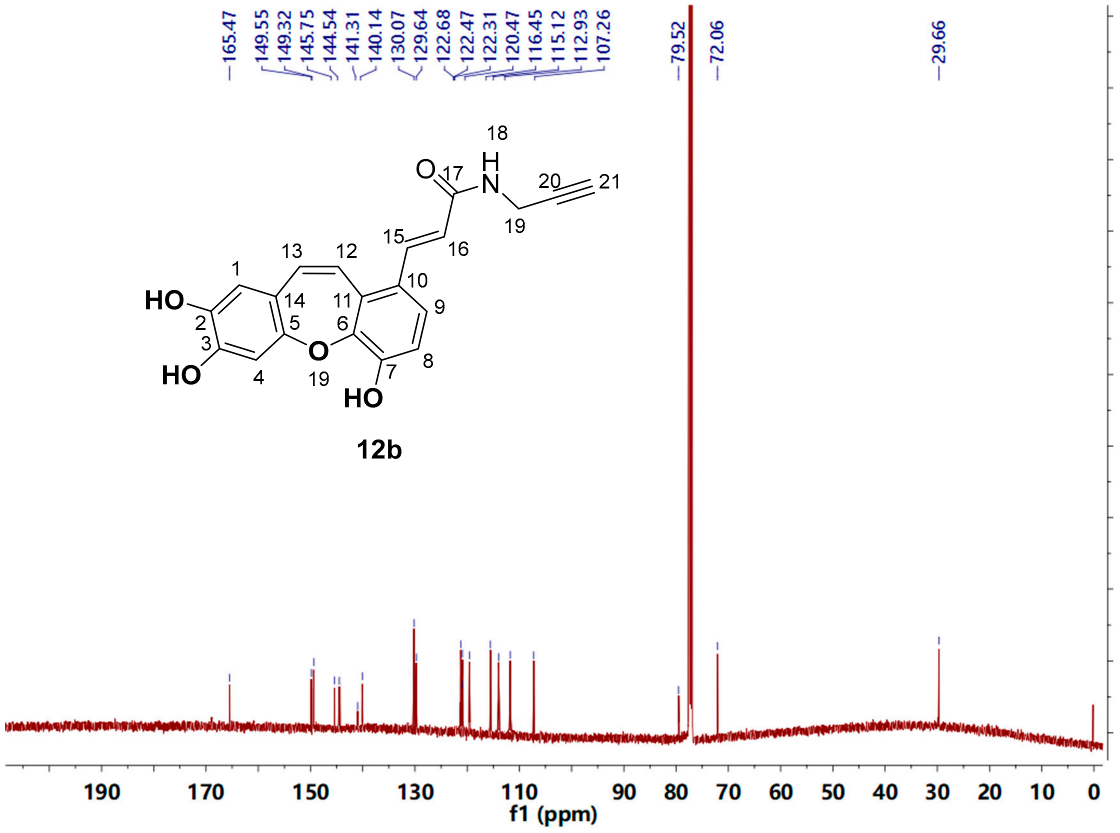

Figure S41. [13] C NMR of compound 12b.

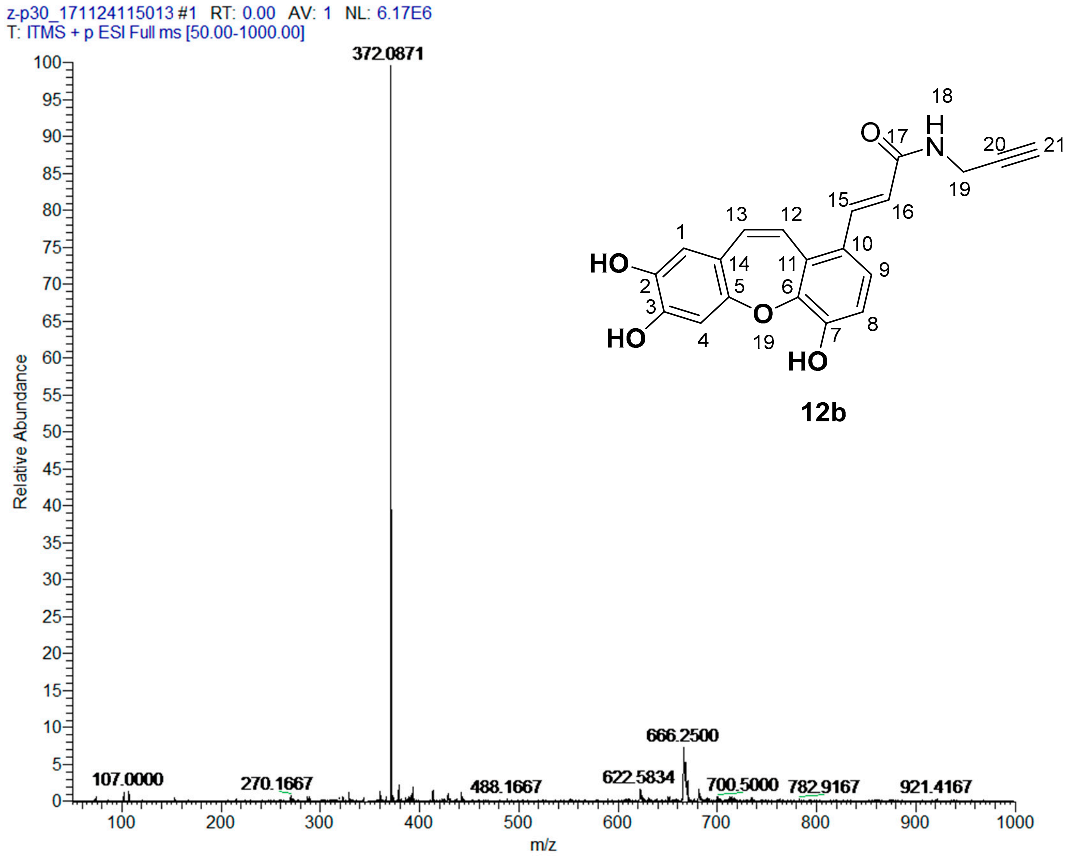

Figure S42. HR-MS of compound 12b.

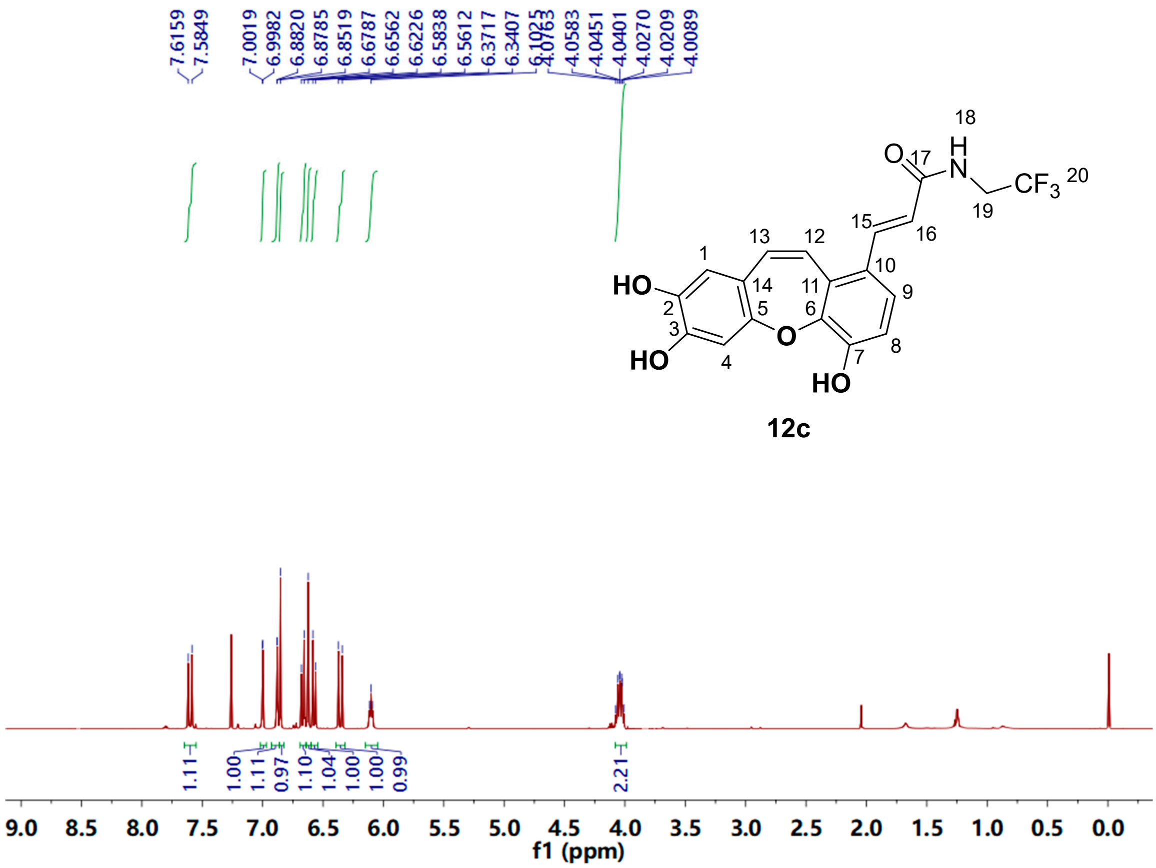

Figure S43. [1] H NMR of compound 12c.

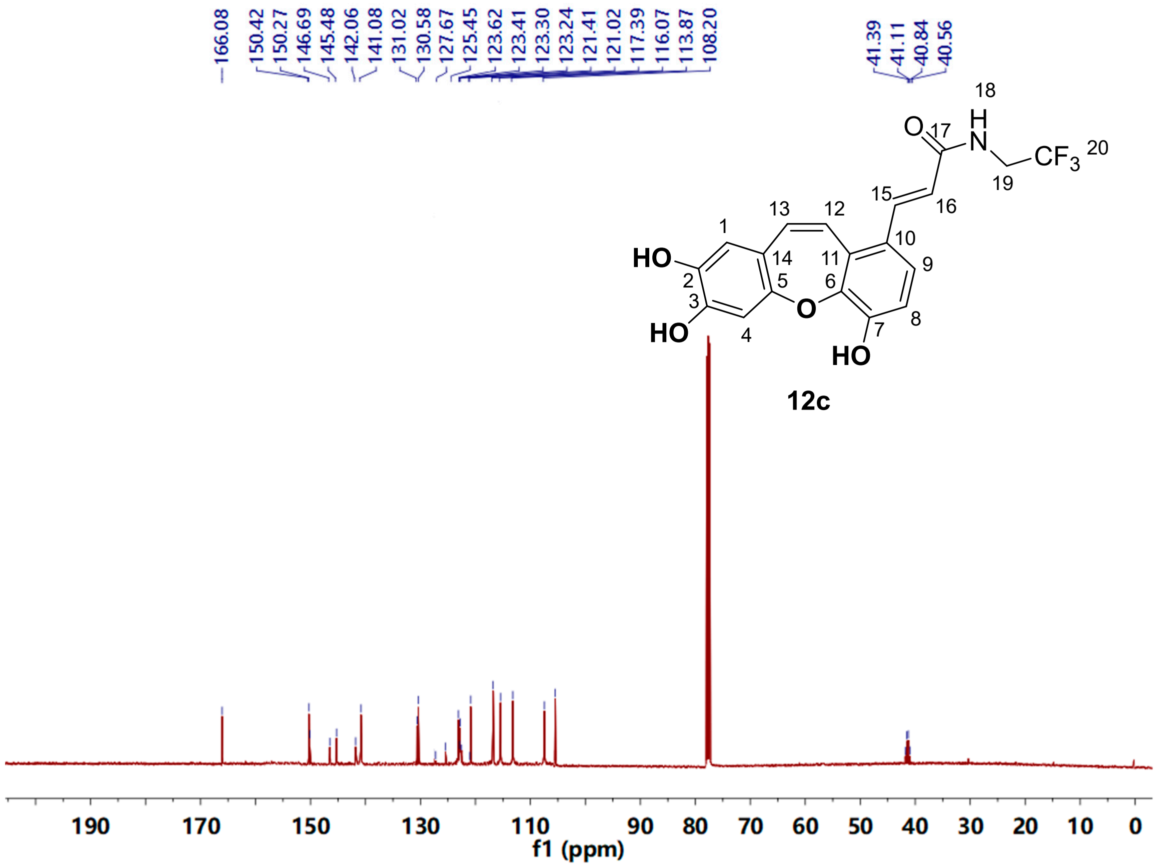

Figure S44. [13] C NMR of compound 12c.

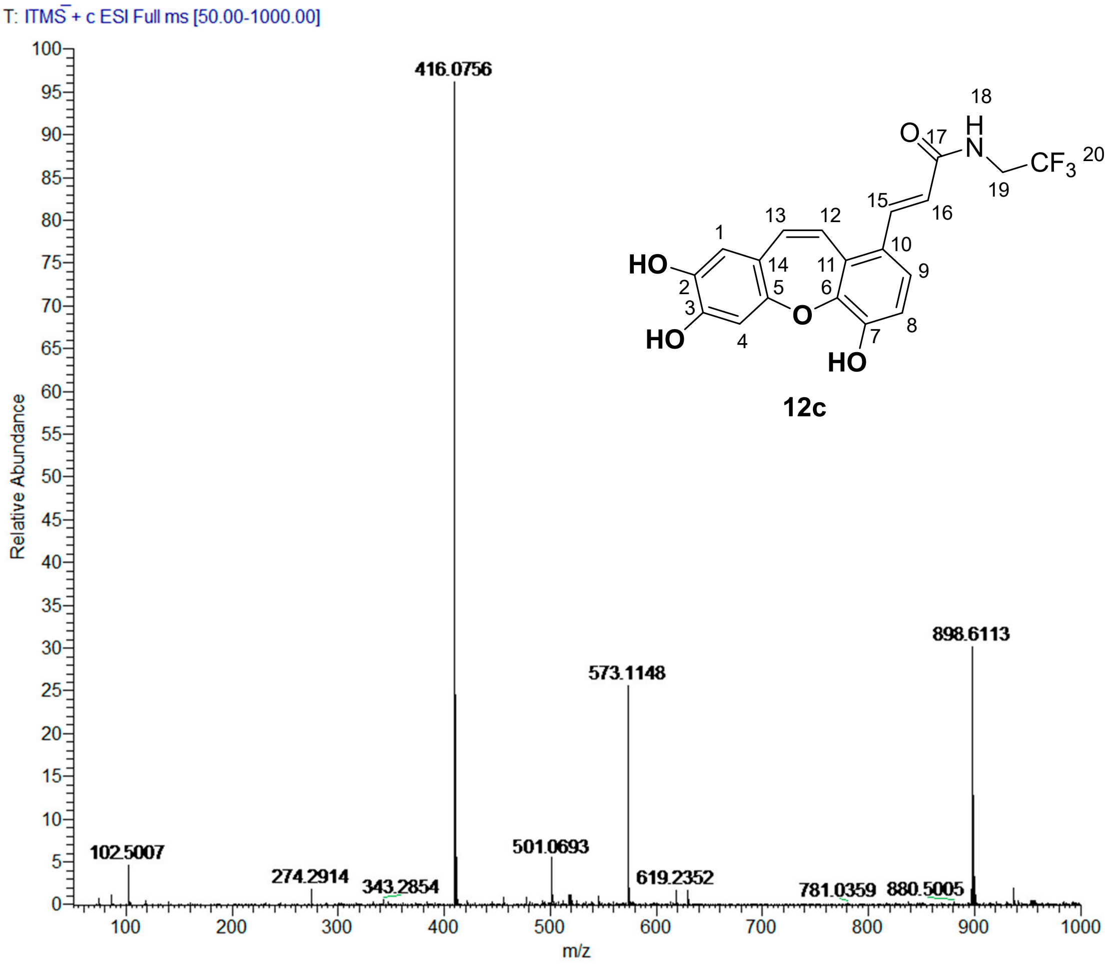

Figure S45. HR-MS of compound 12c.

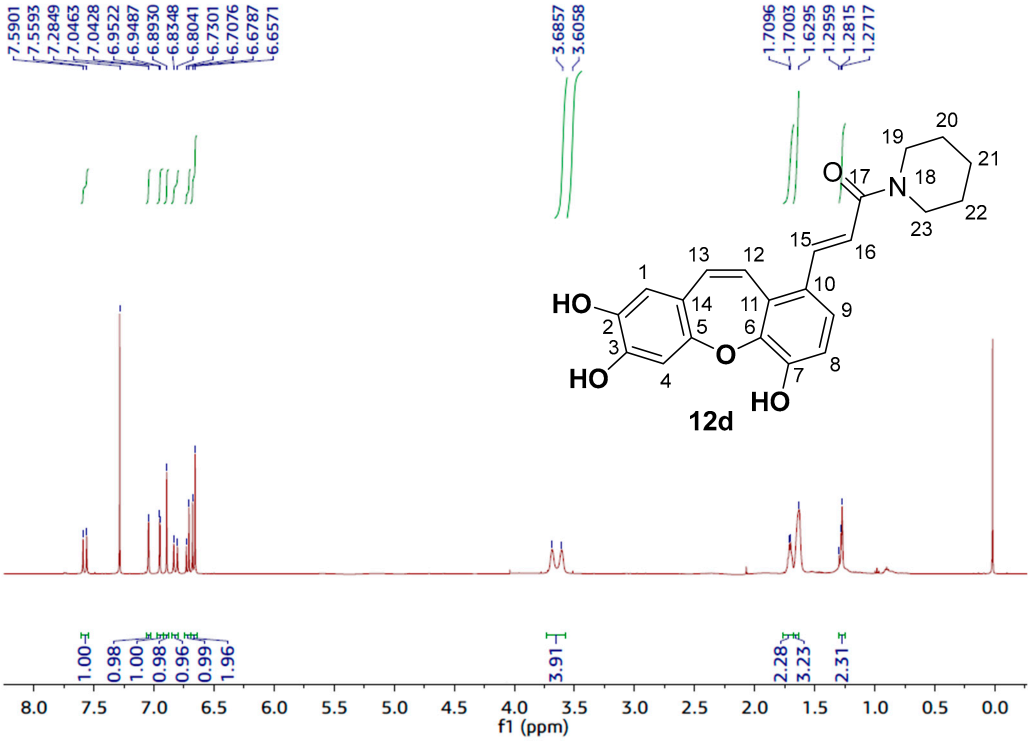

Figure S46. [1] H NMR of compound 12d.

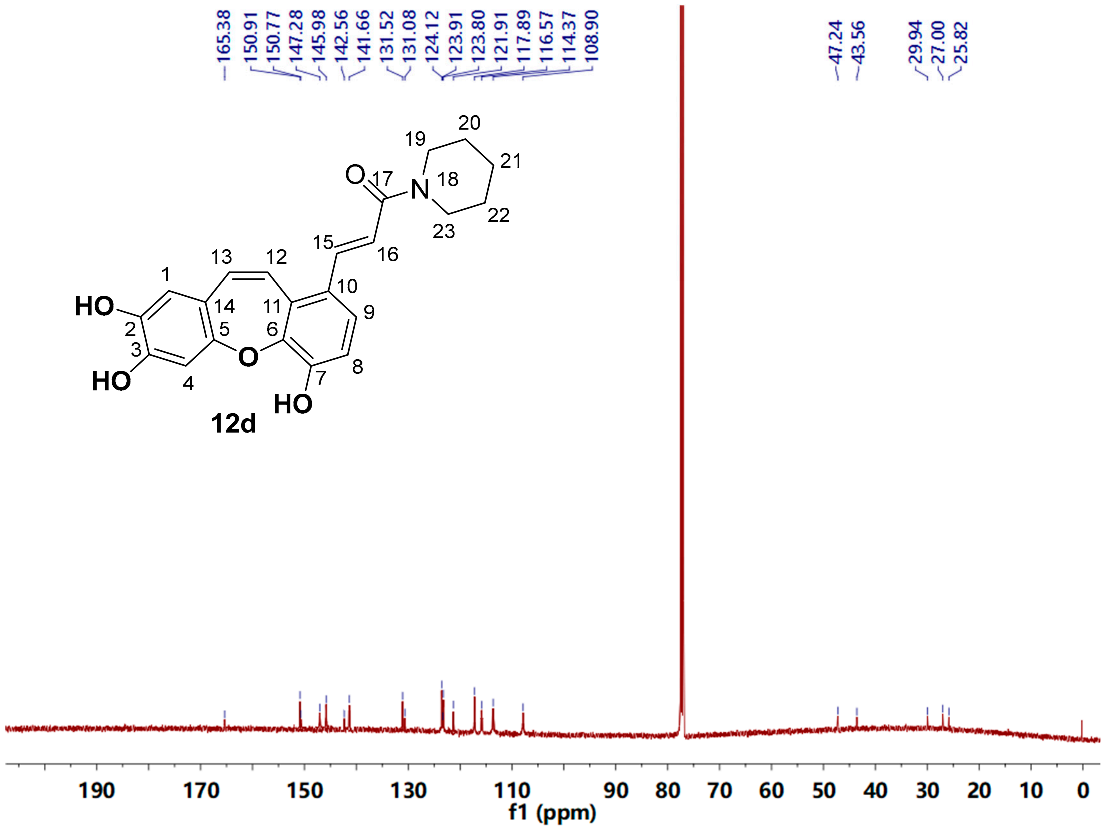

Figure S47. ^{13} C NMR of compound 12d.

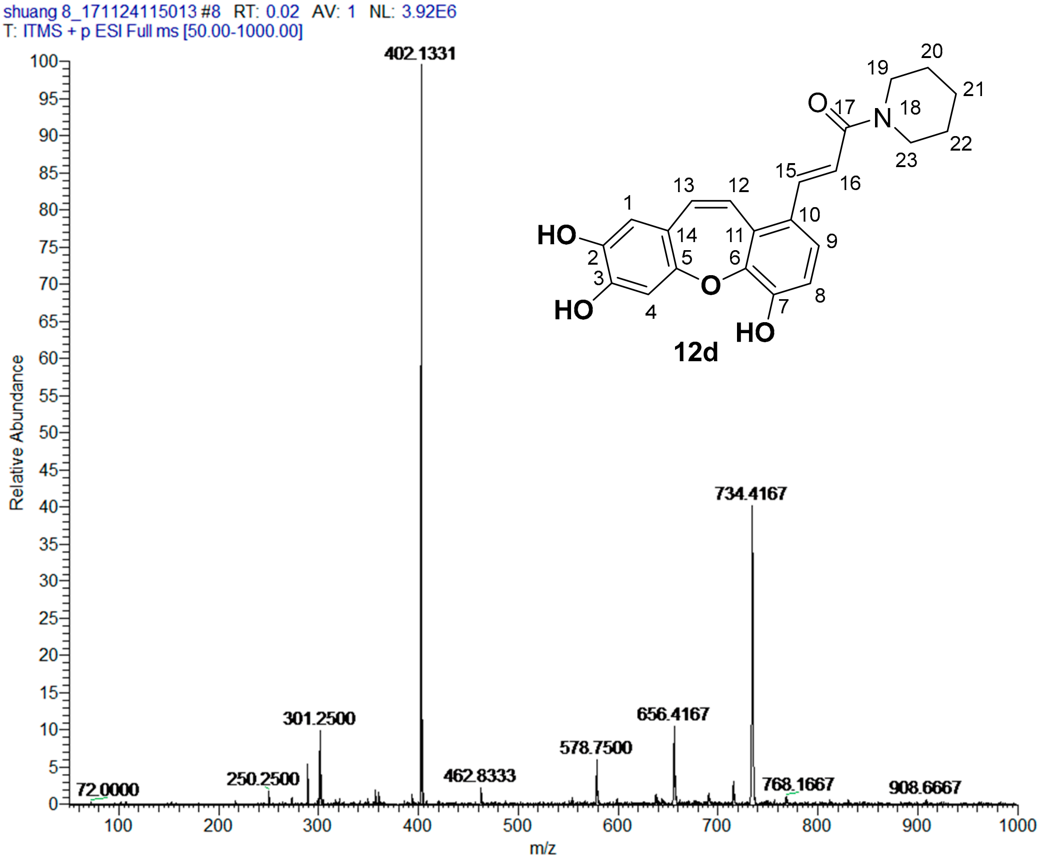

Figure S48. HR-MS of compound 12d.

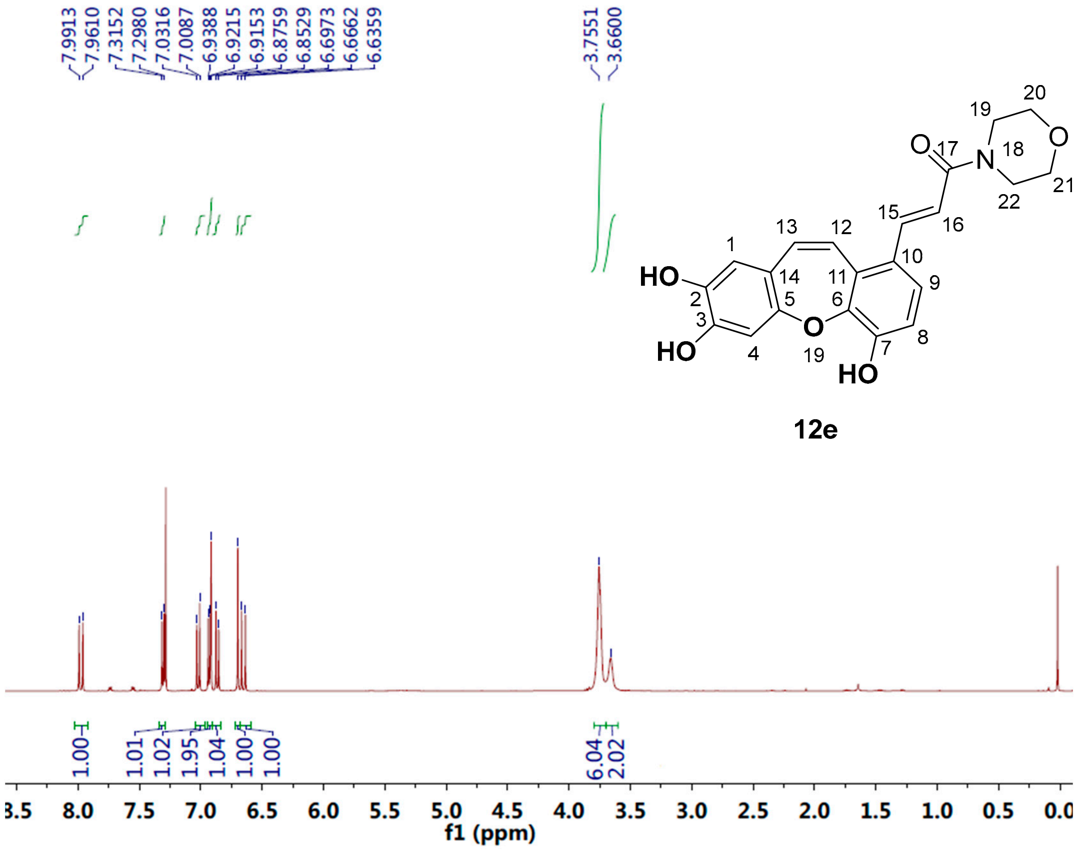

Figure S49. [1] H NMR of compound 12e.

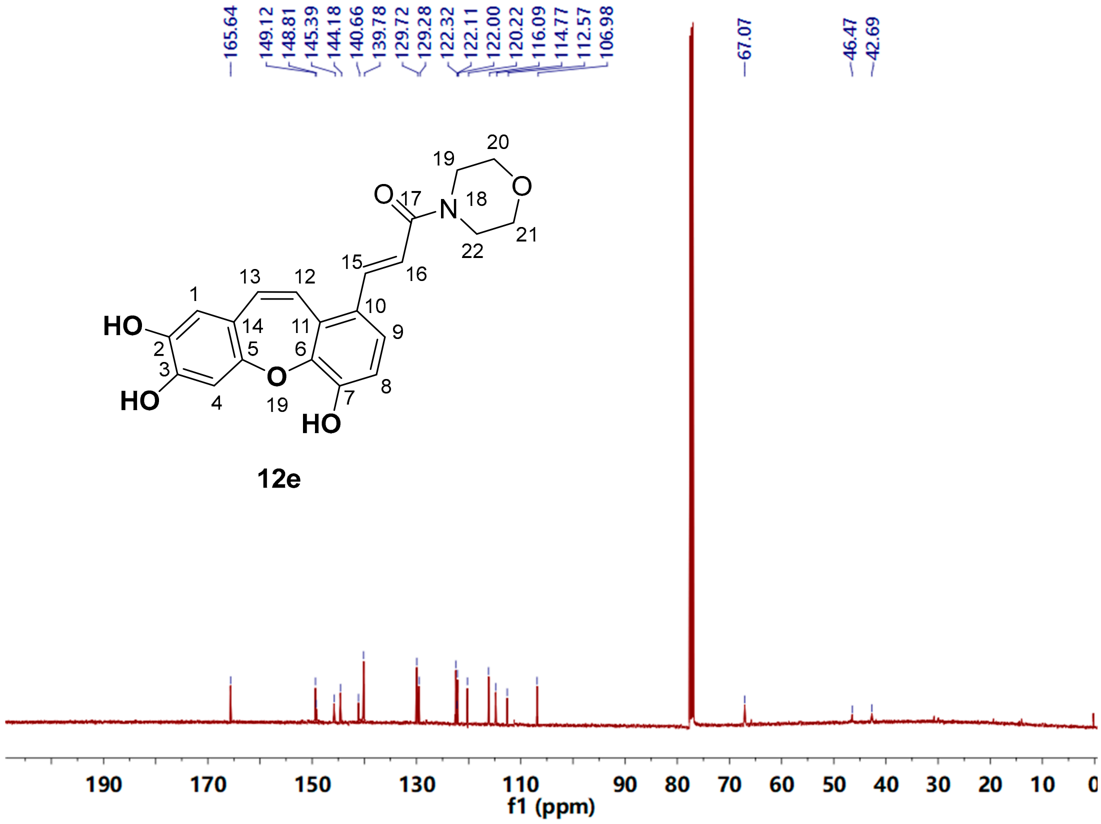

Figure S50. [13] C NMR of compound 12e.

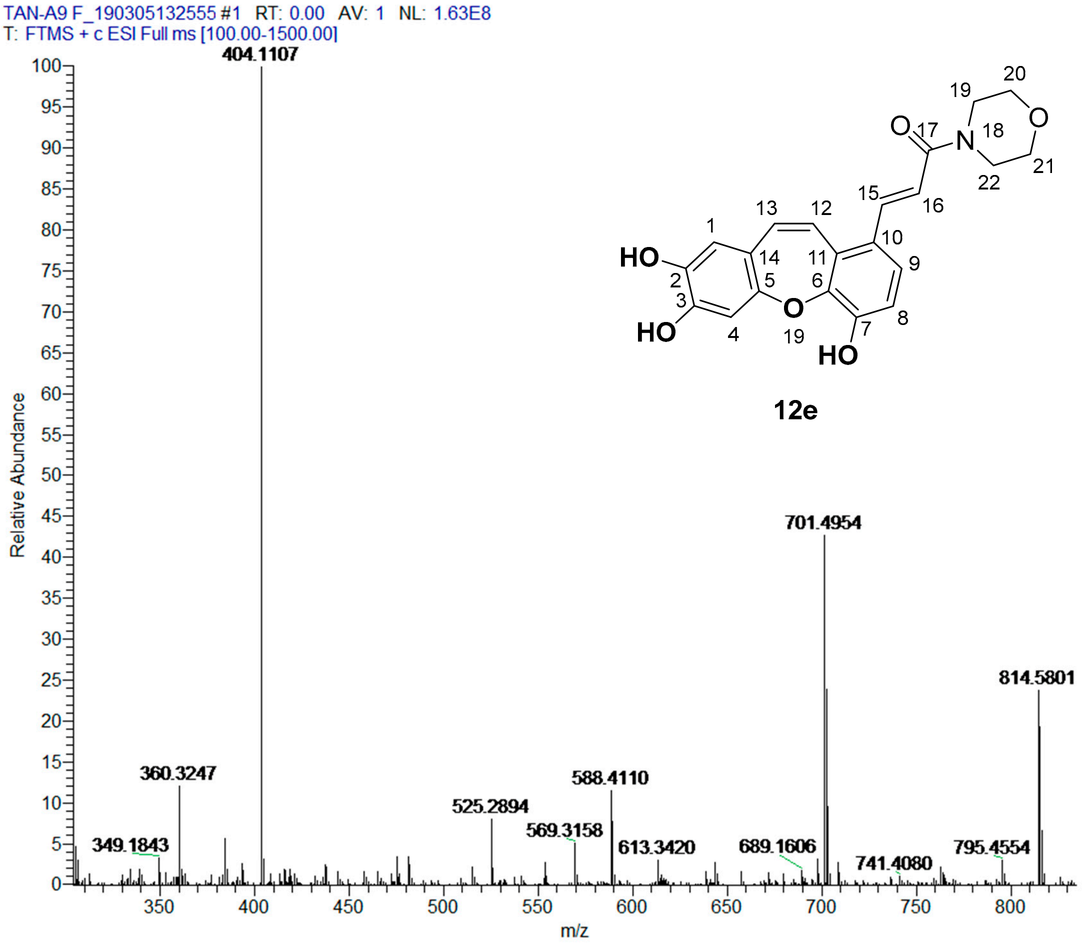

Figure S51. HR-MS of compound 12e.

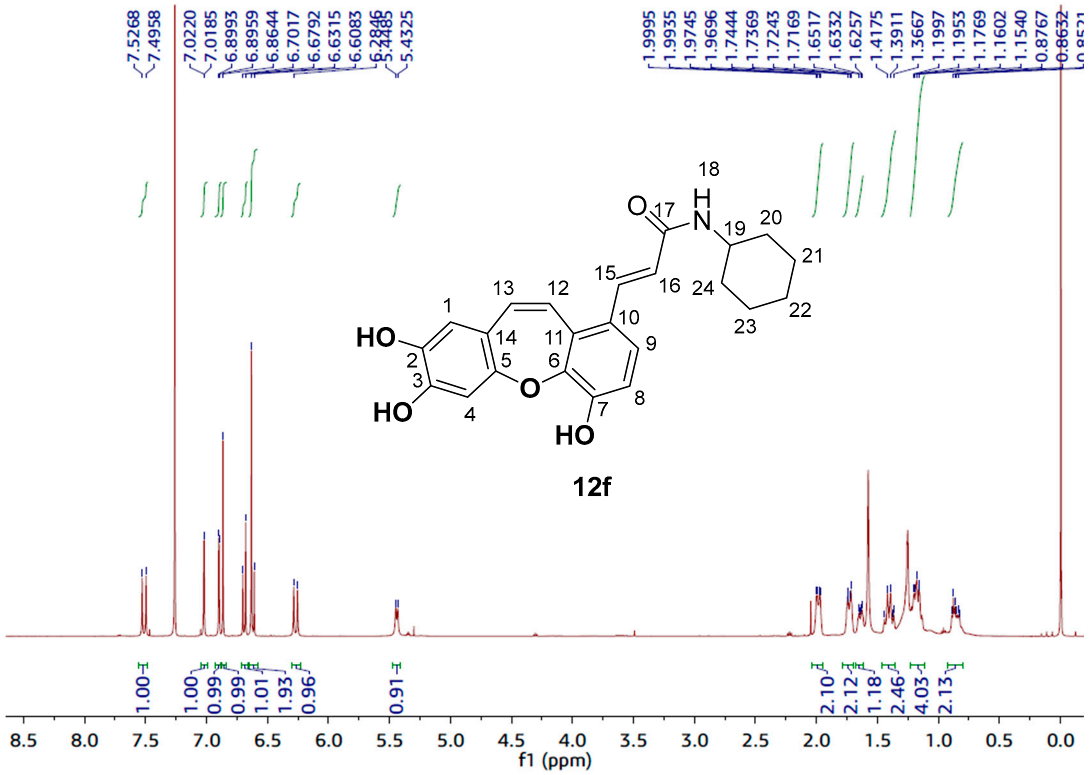

Figure S52. [1] H NMR of compound 12f.

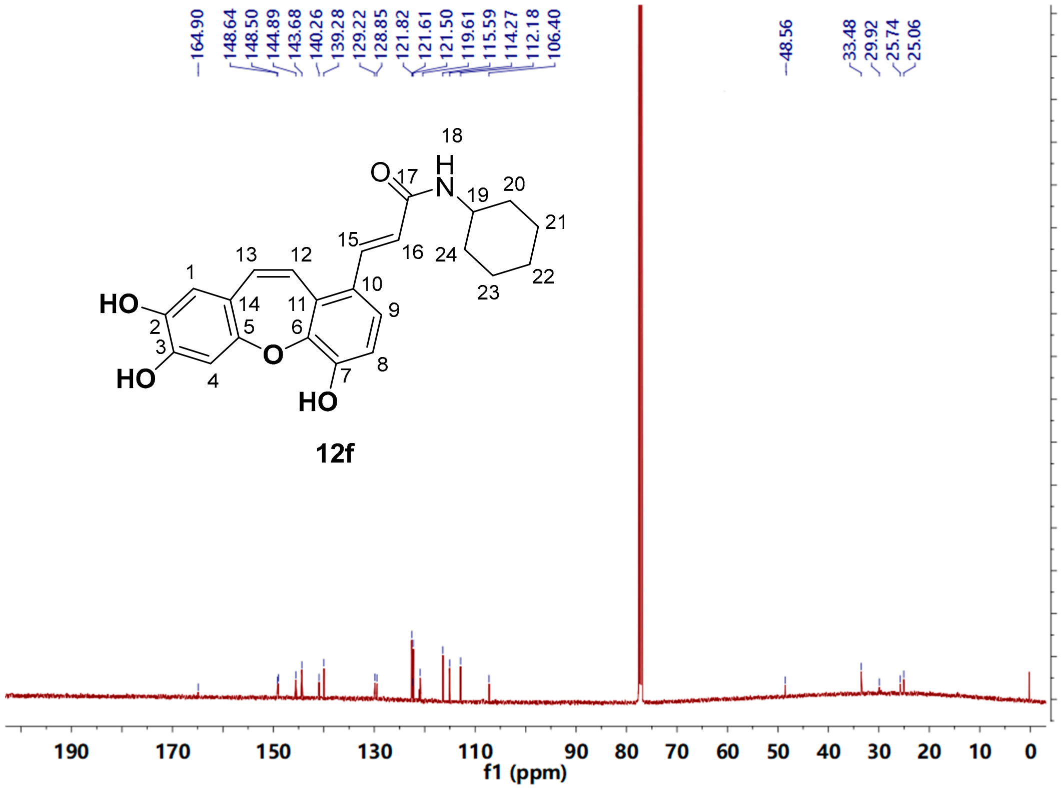

Figure S53. [13] C NMR of compound 12f.

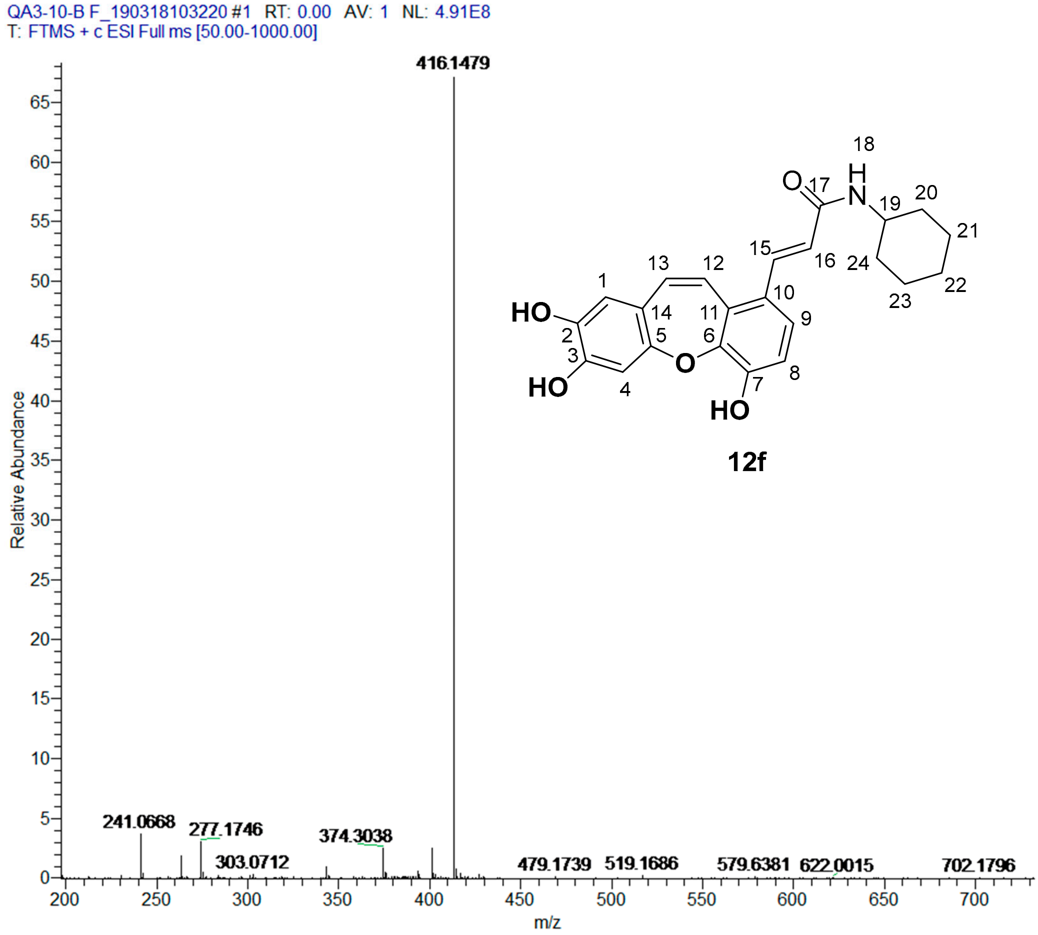

Figure S54. HR-MS of compound 12f.

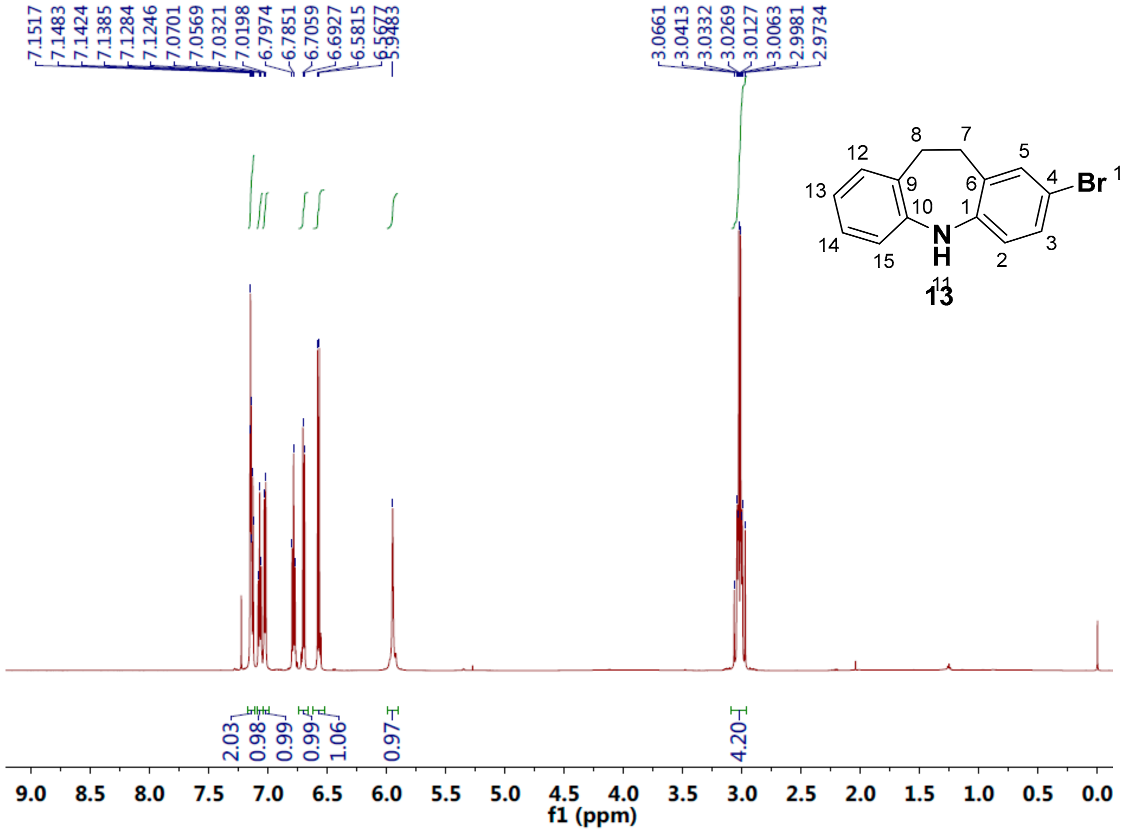

Figure S55. [1] H NMR of compound 13.

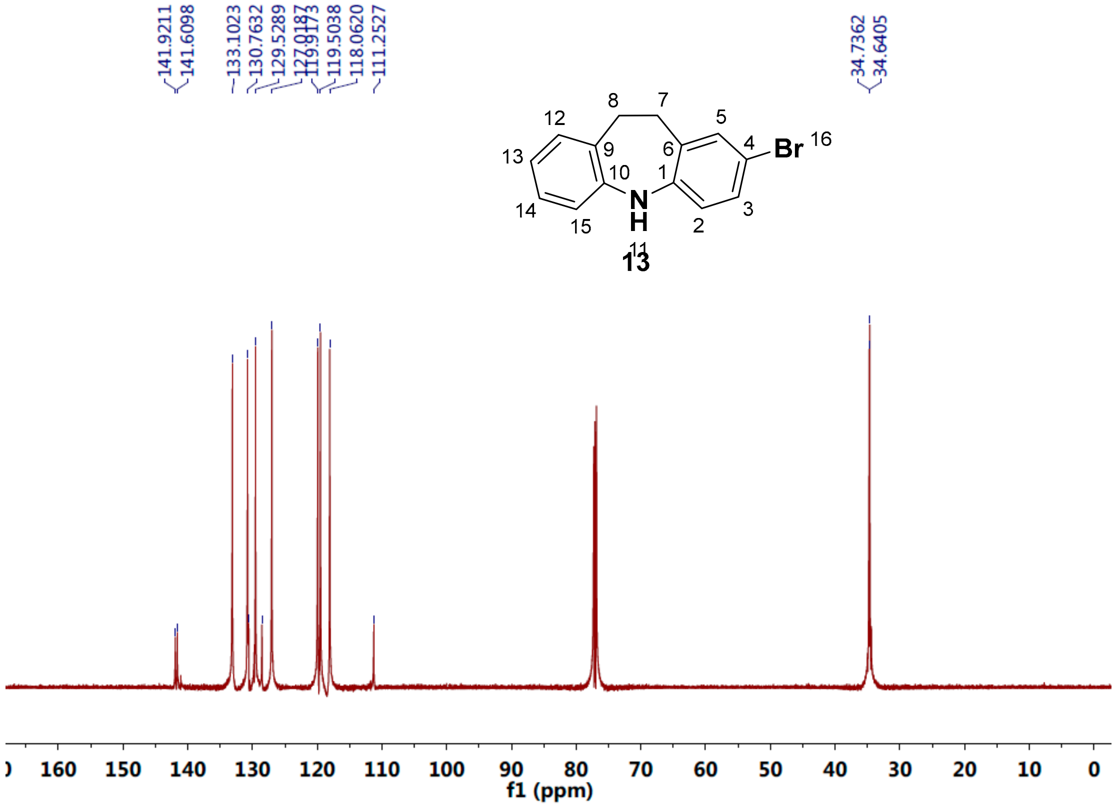

Figure S56. [13] C NMR of compound 13.

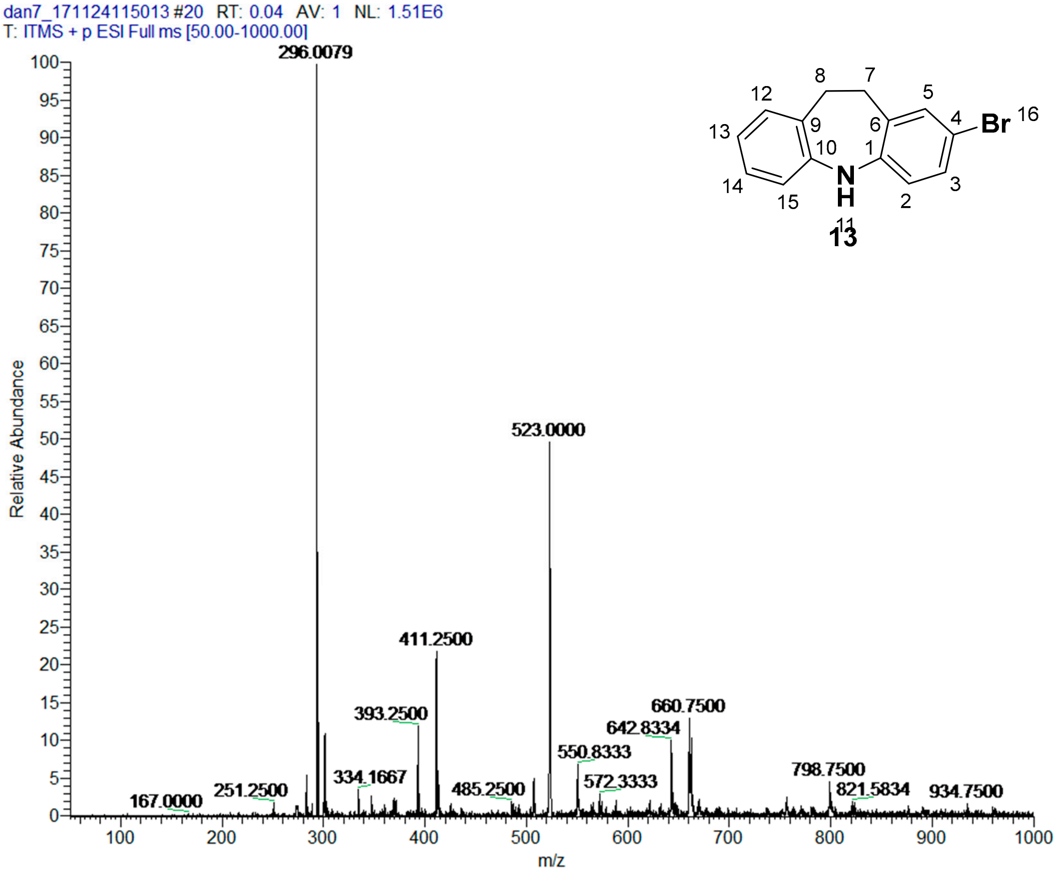

Figure S57. HR-MS of compound 13.

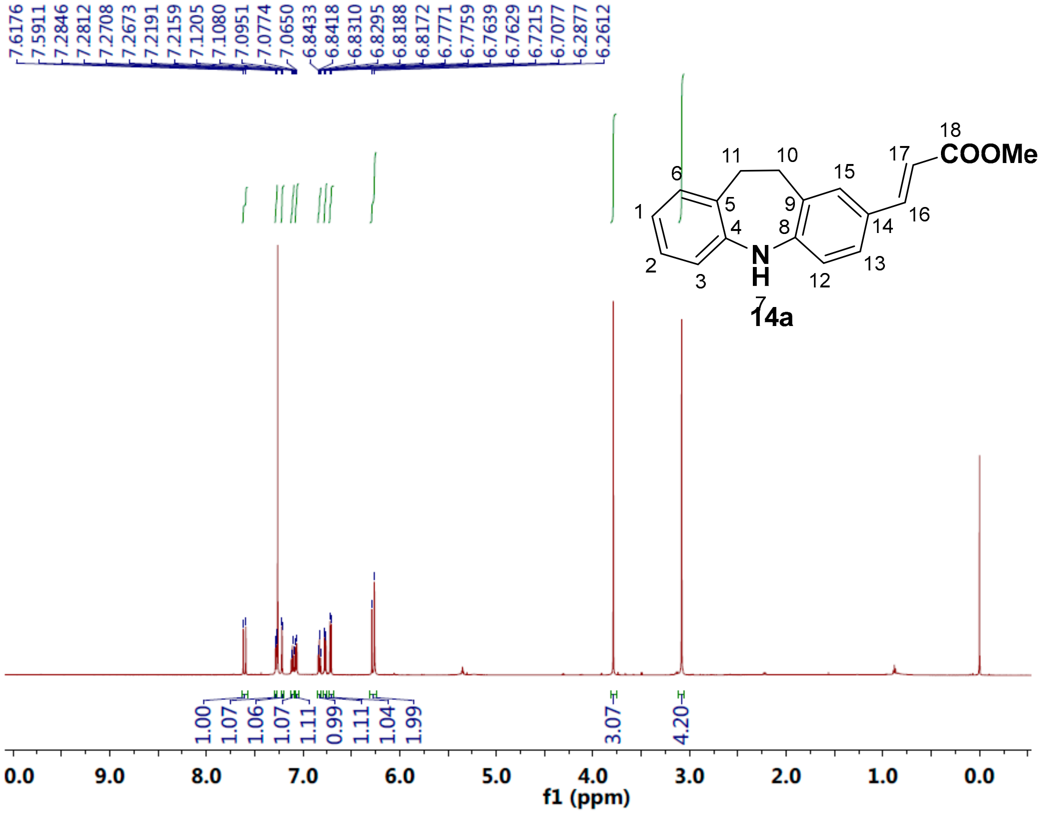

Figure S58. [1] H NMR of compound 14a.

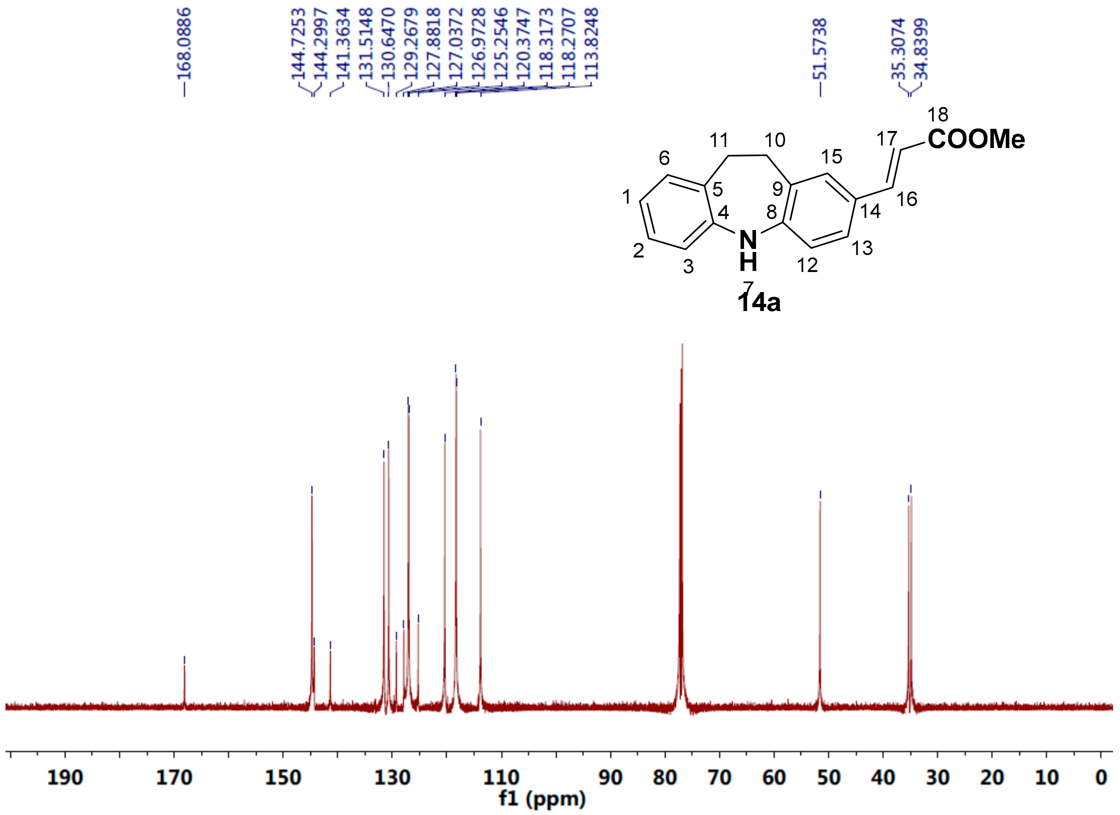

Figure S59. [13] C NMR of compound 14a.

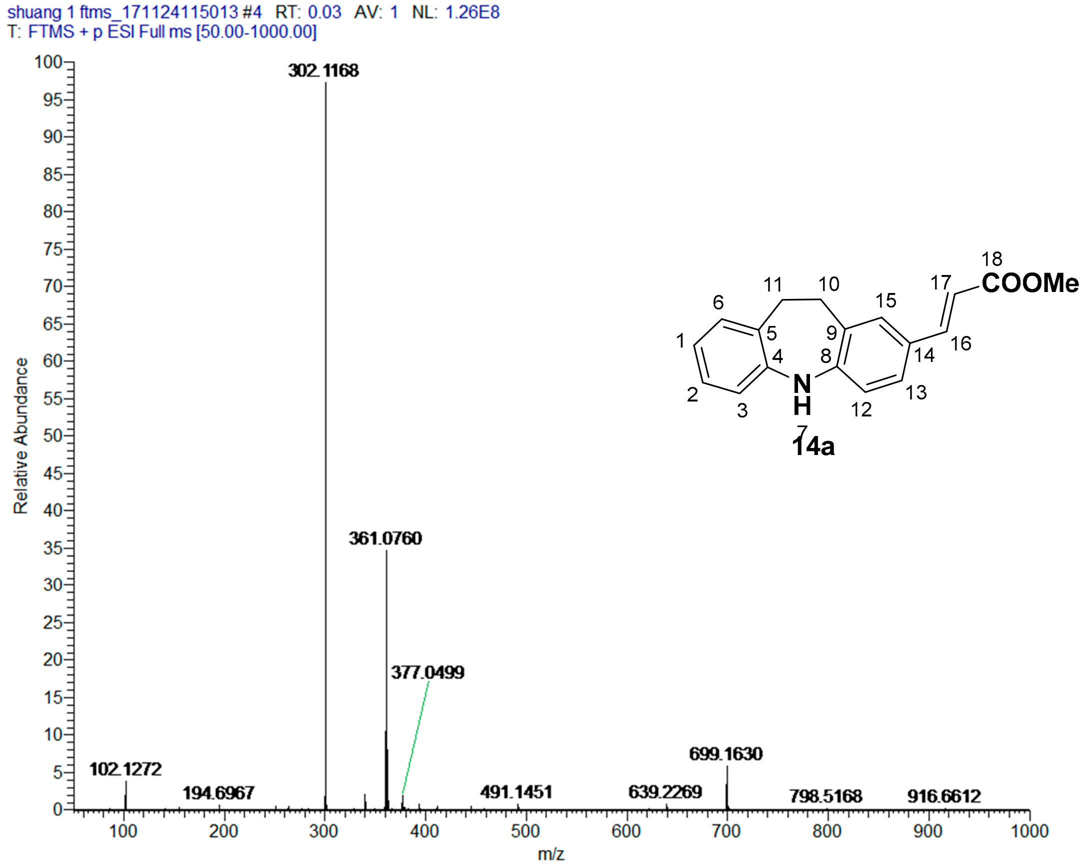

Figure S60. HR-MS of compound 14a.

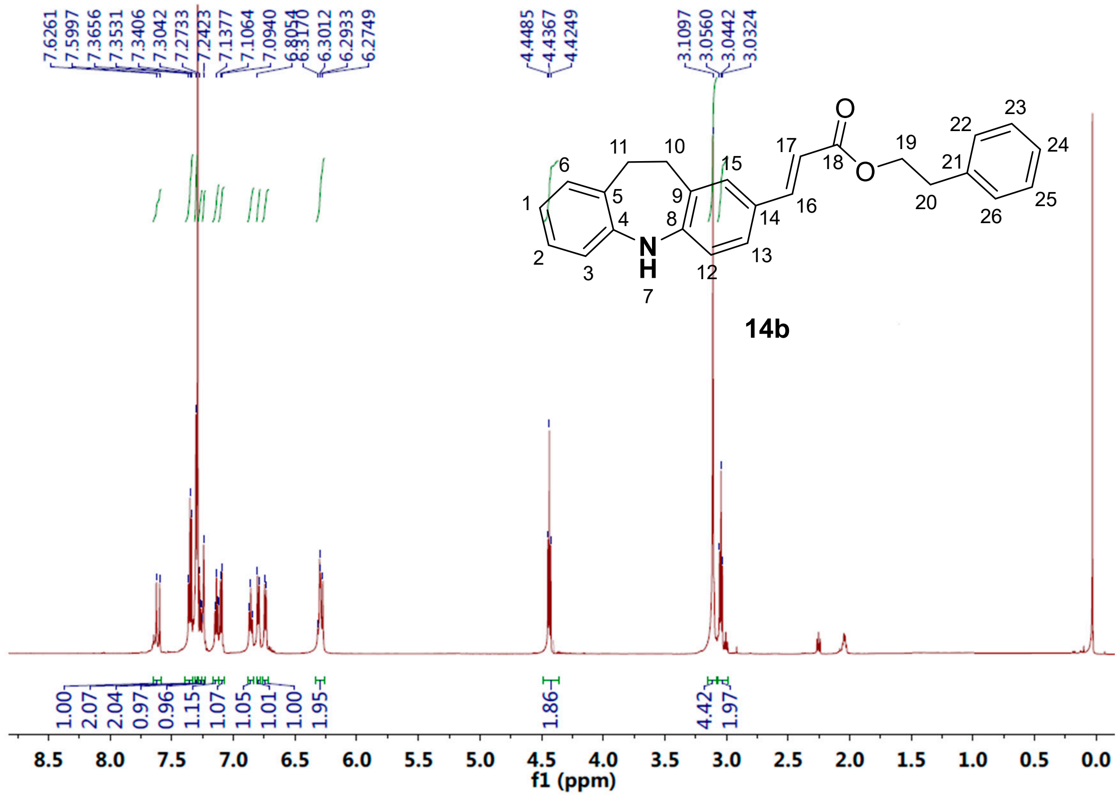

Figure S61. [1] H NMR of compound 14b.

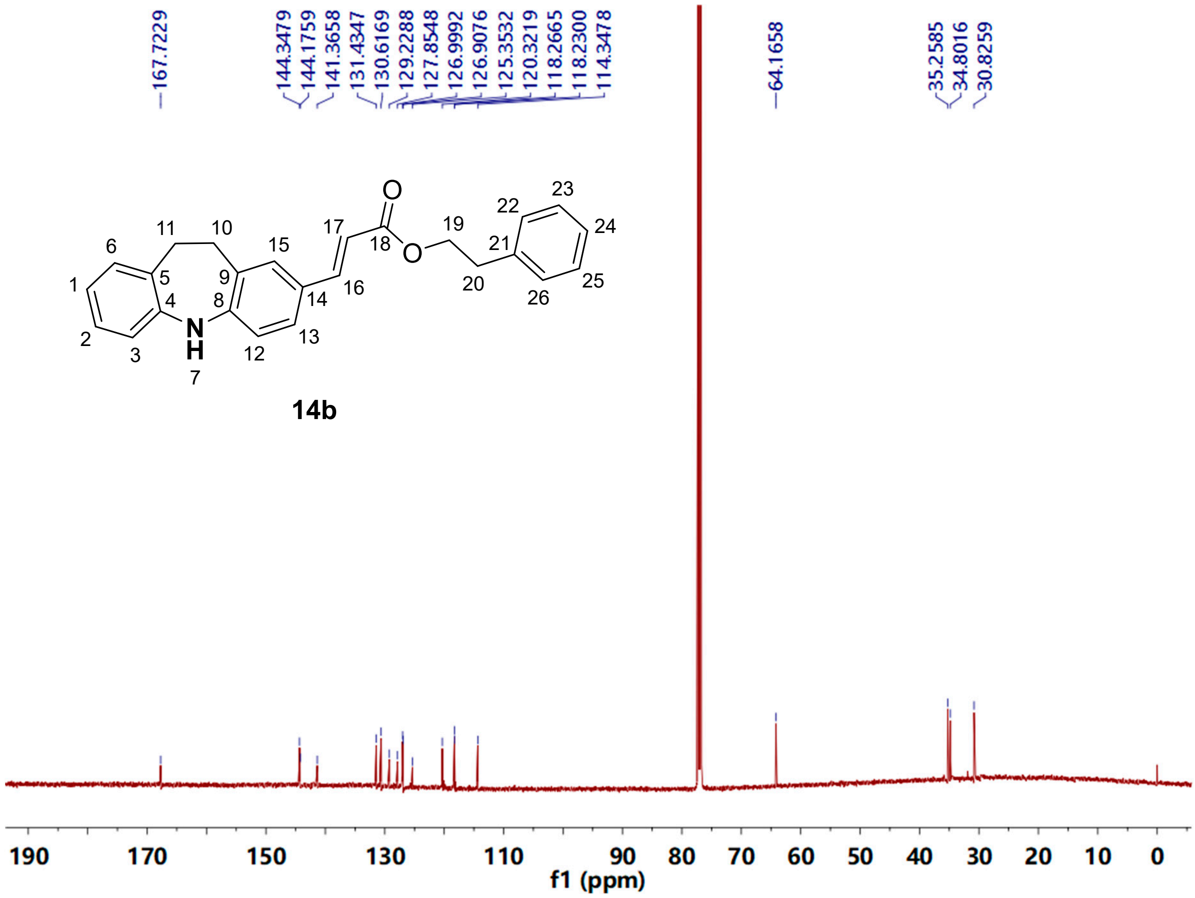

Figure S62. [13] C NMR of compound 14b.

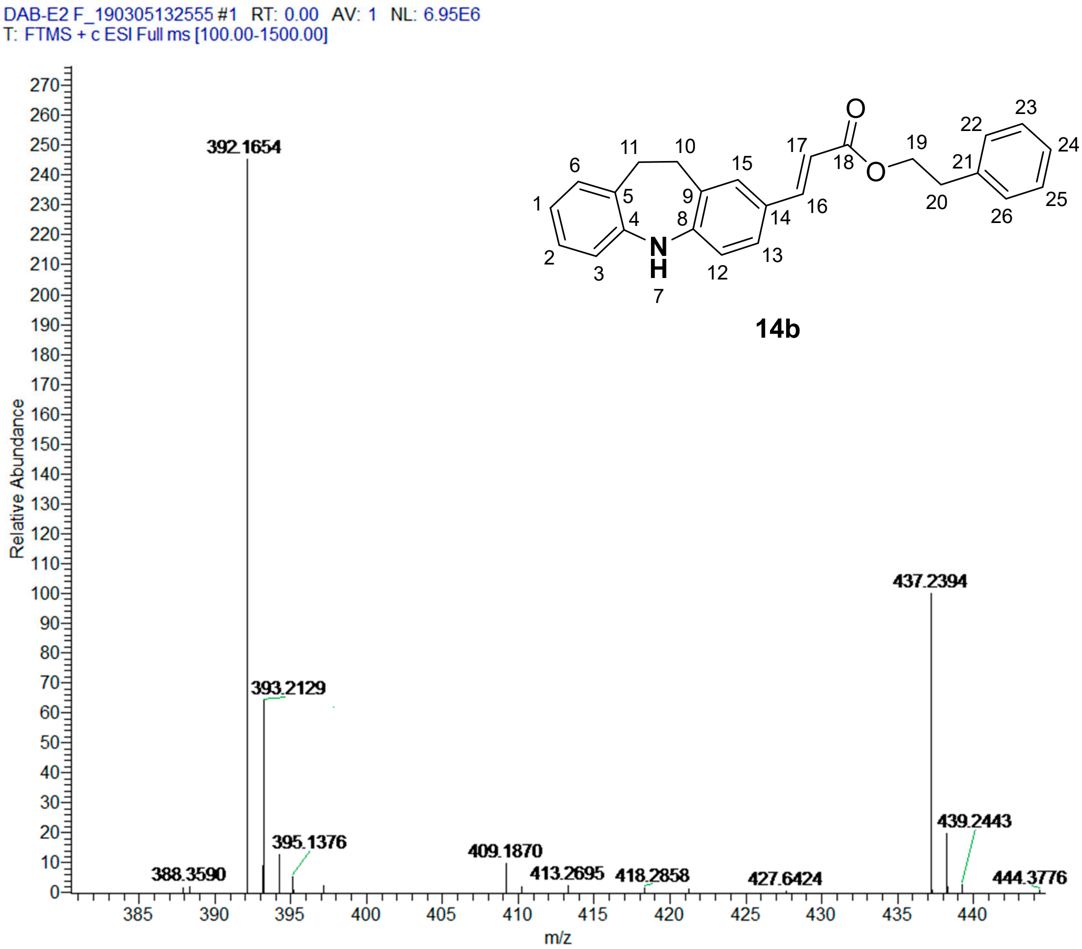

Figure S63. HR-MS of compound 14b.

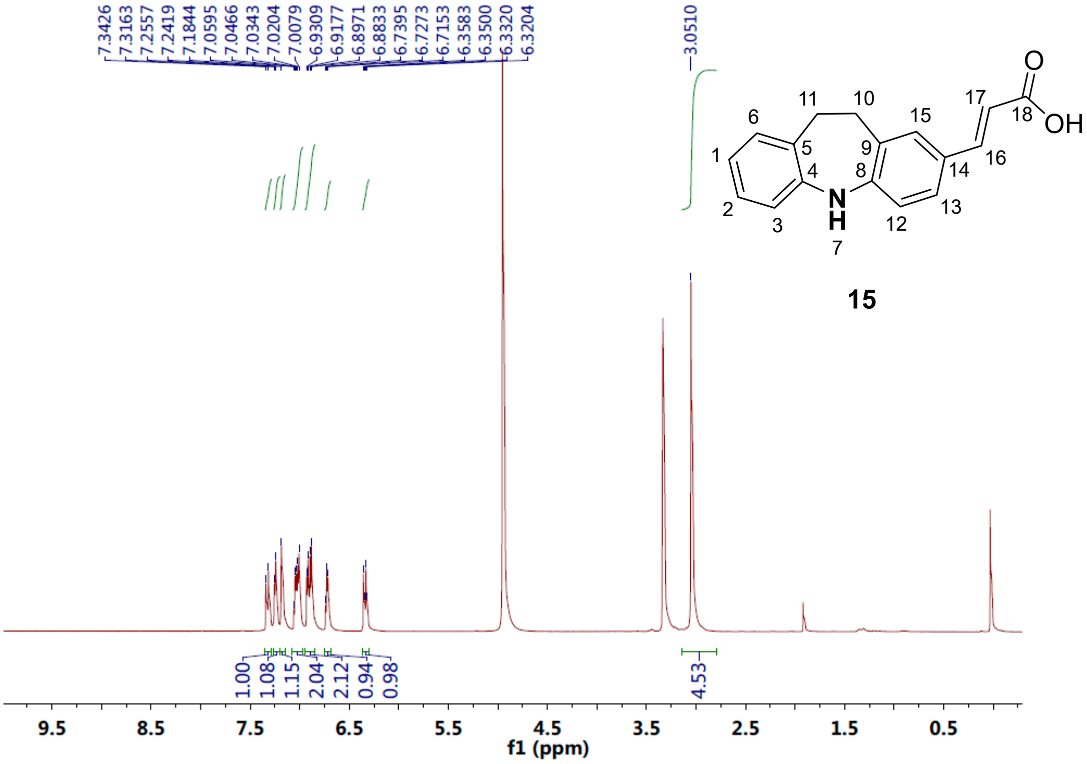

Figure S64. ¹ H NMR of compound 15.

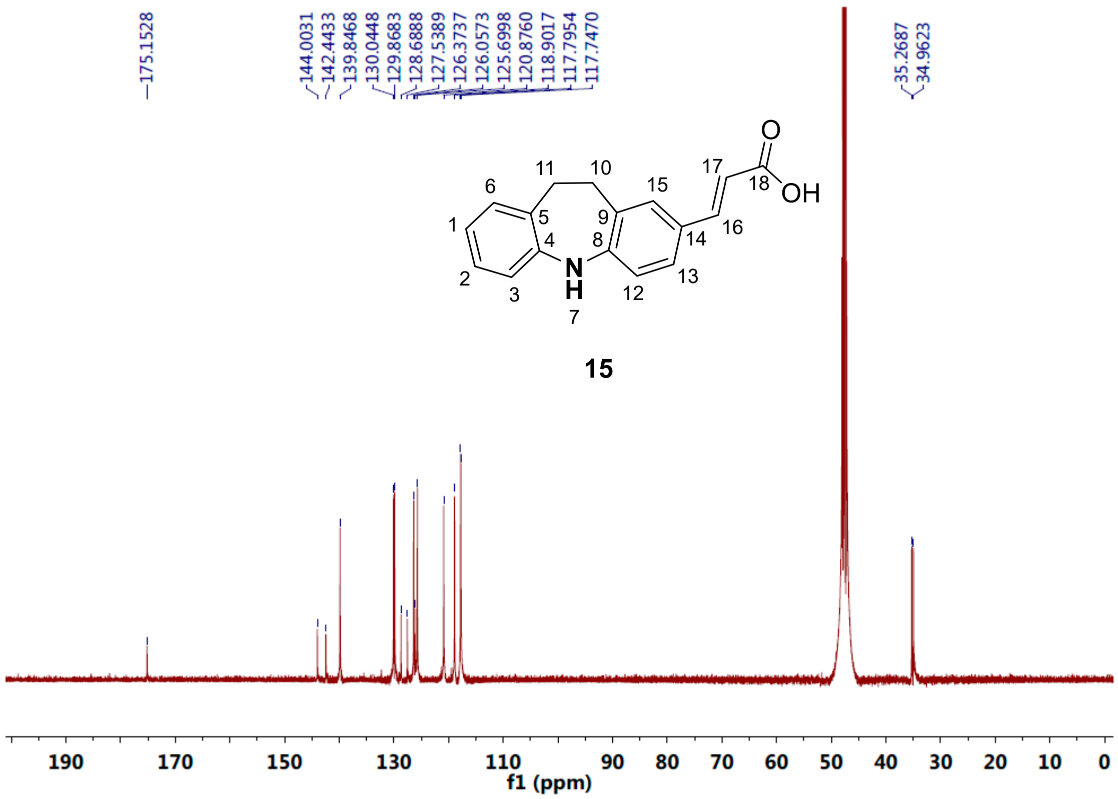

Figure S65. [13] C NMR of compound 15.

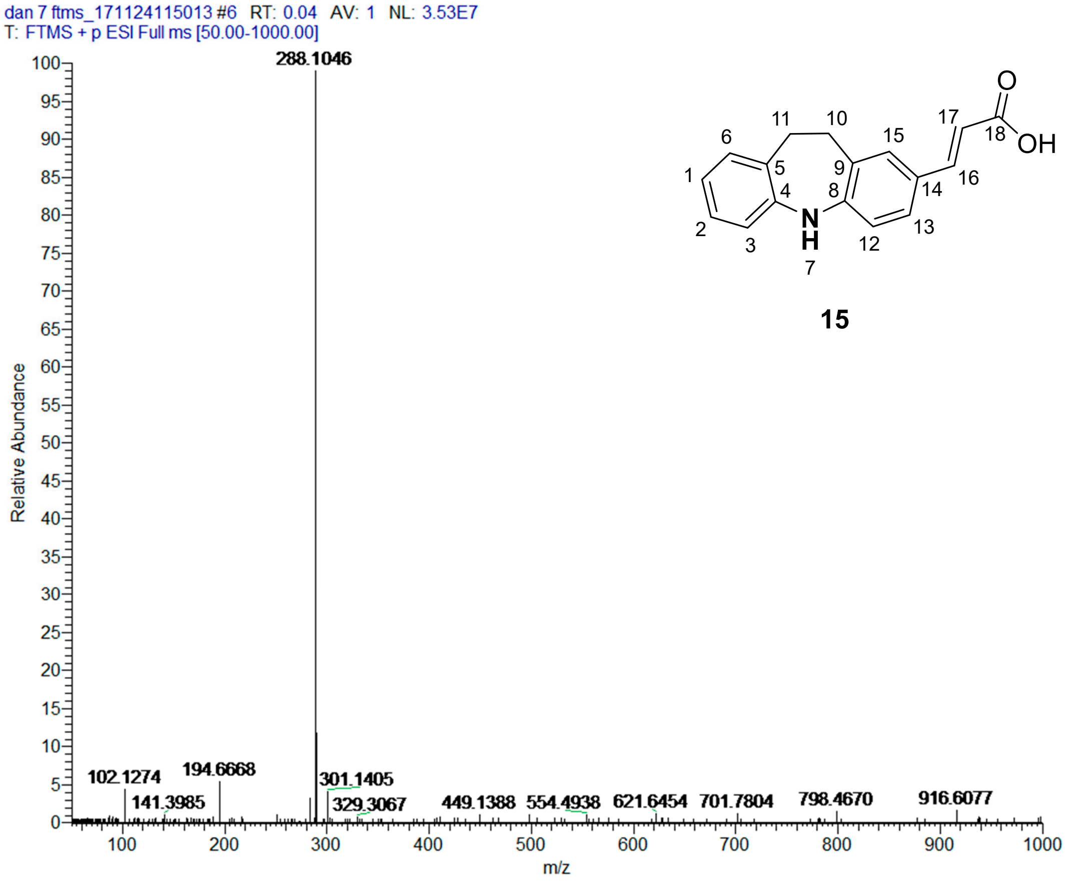

Figure S66. HR-MS of compound 15.

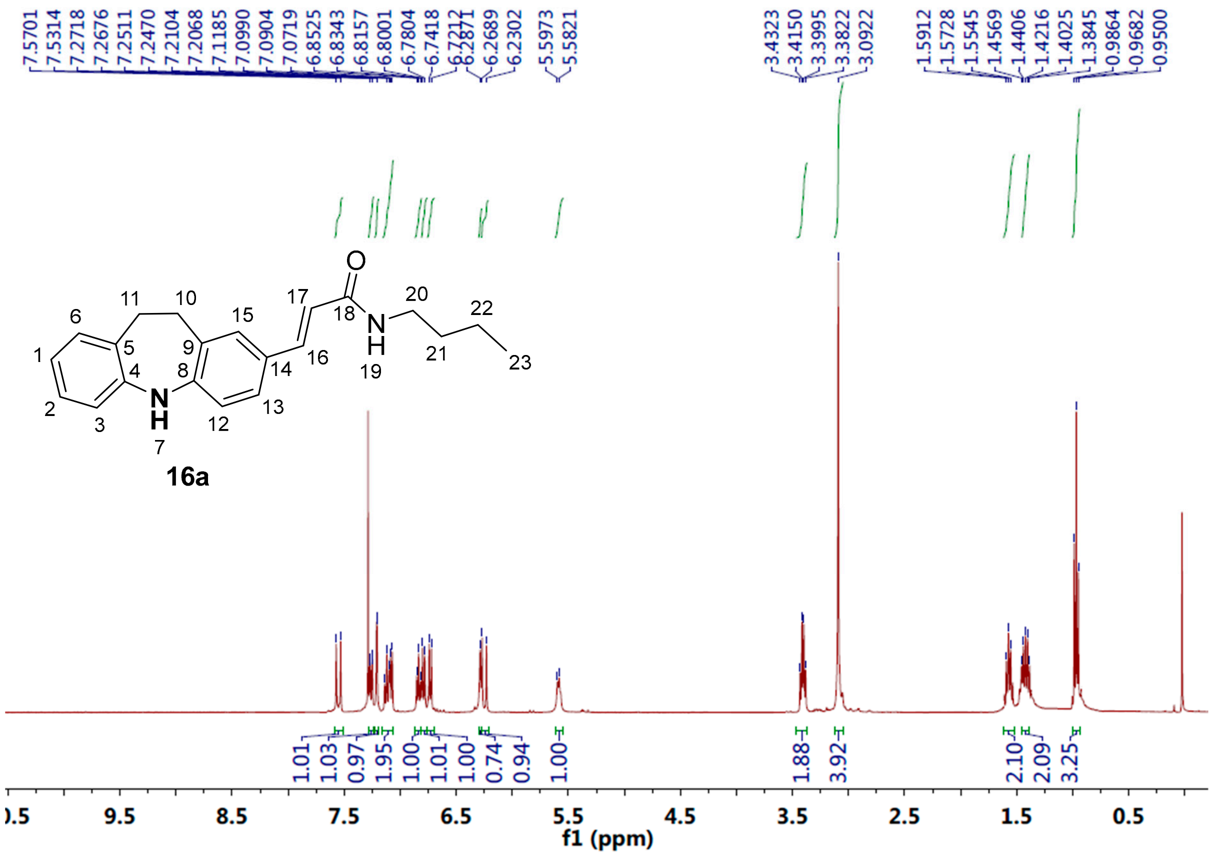

Figure S67. [1] H NMR of compound 16a.

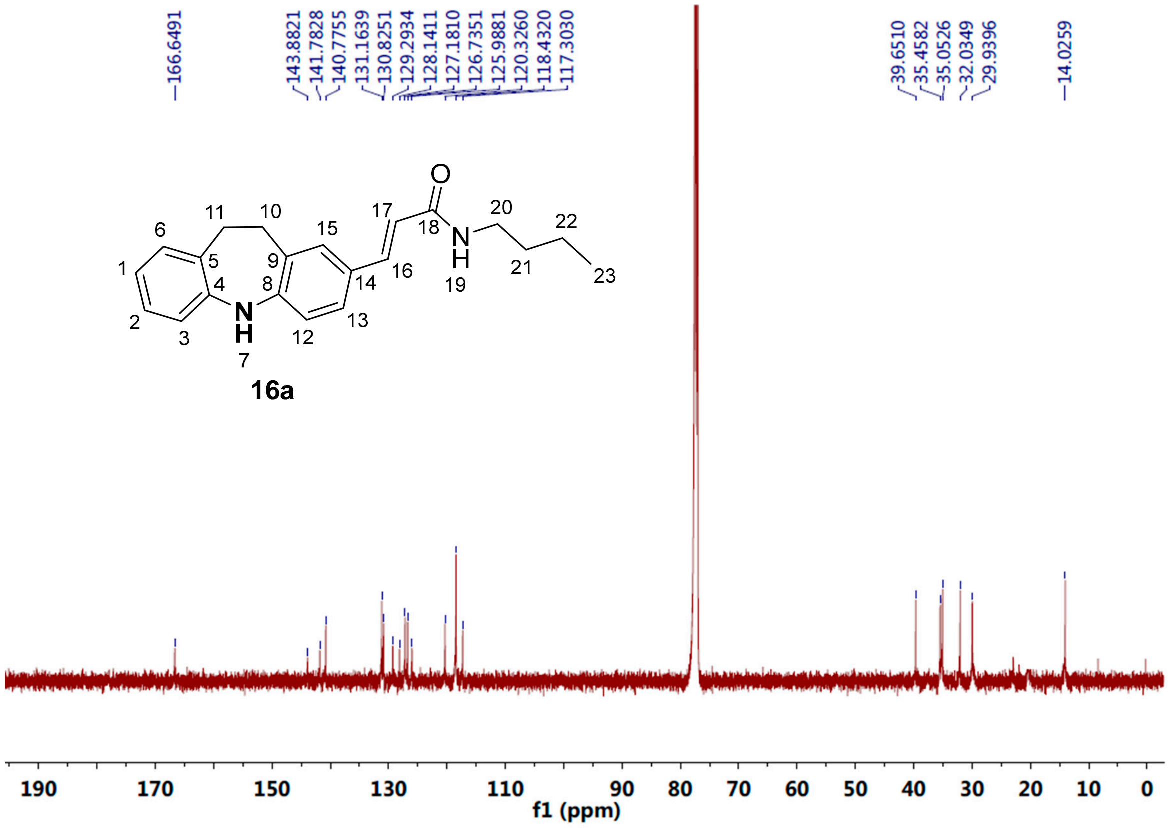

Figure S68. [13] C NMR of compound 16a.

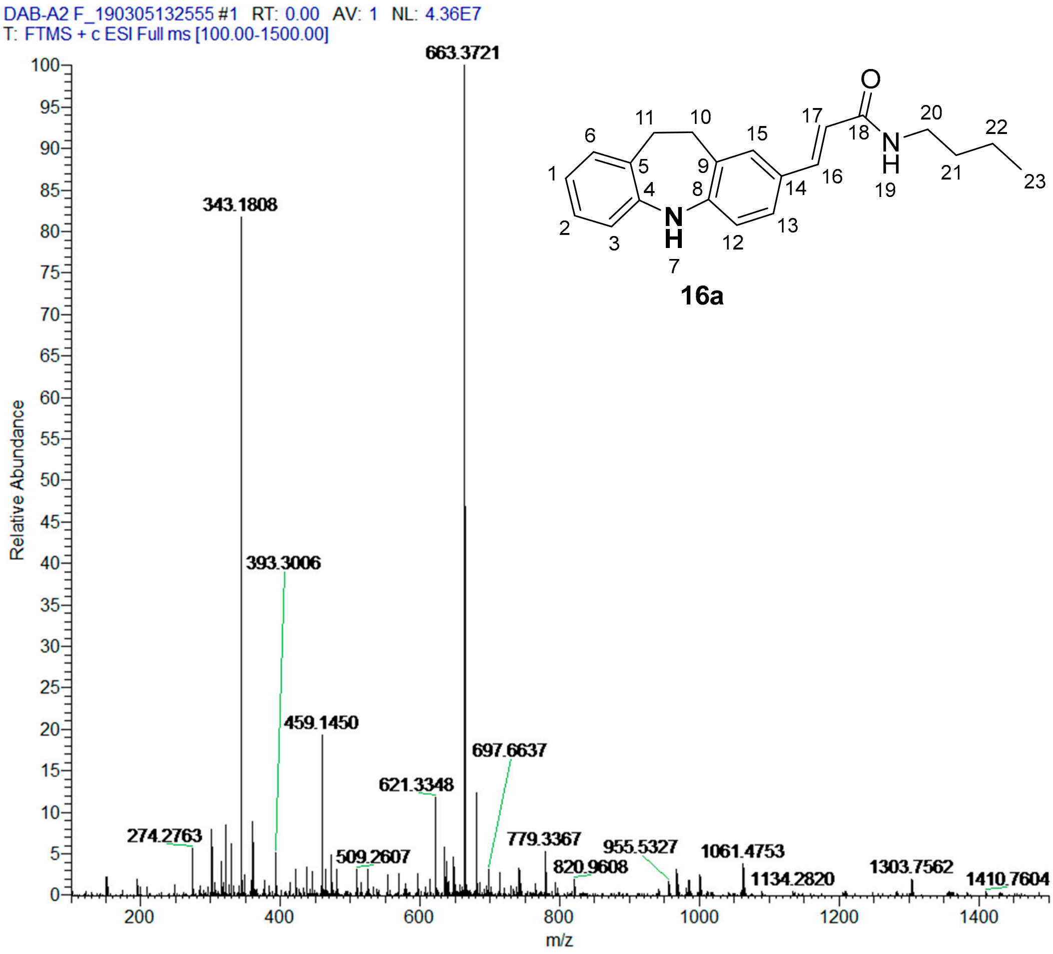

Figure S69. HR-MS of compound 16a.

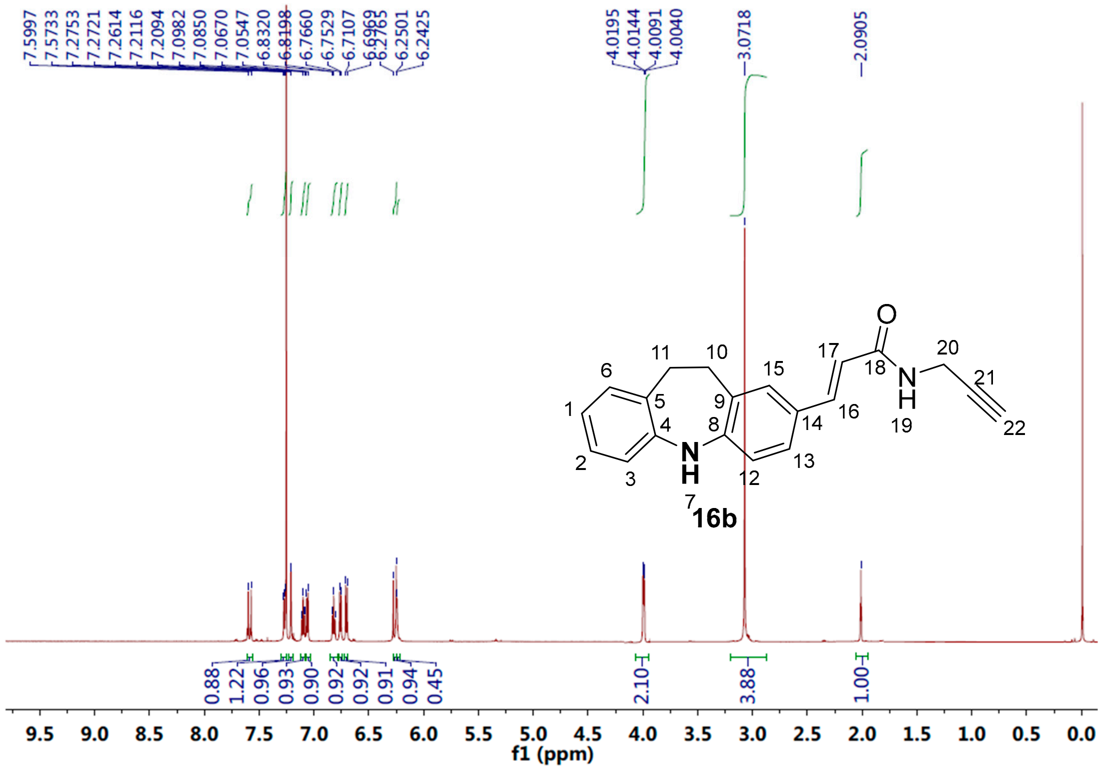

Figure S70. ^{1}H NMR of compound 16b.

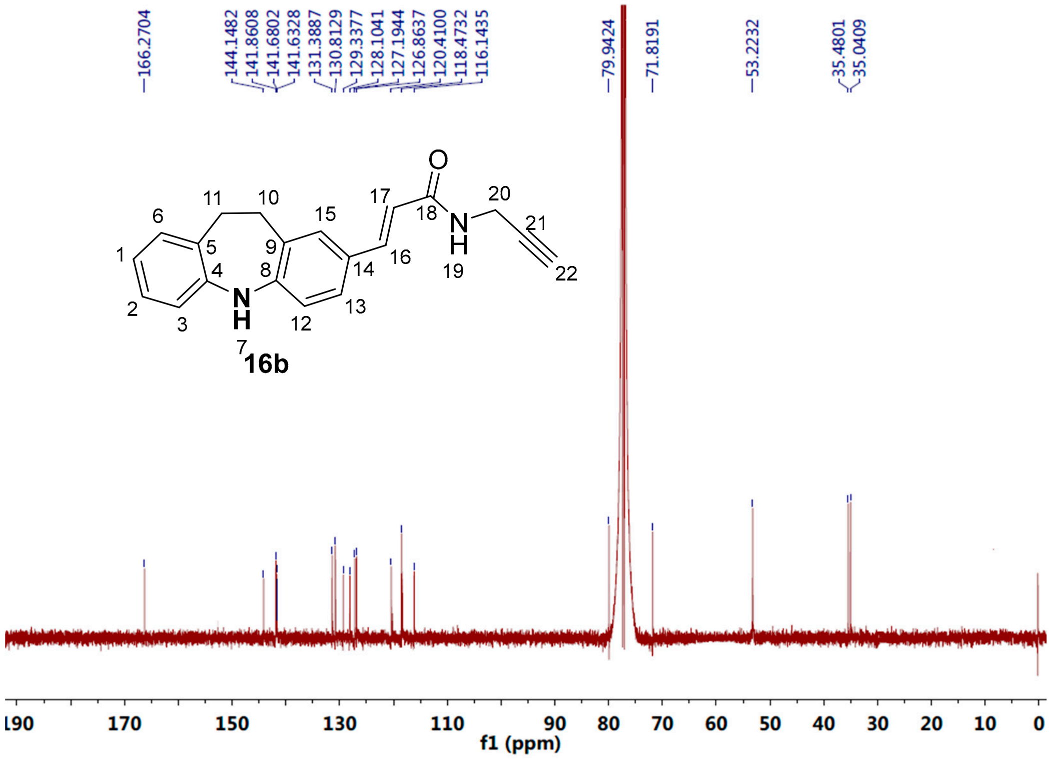

Figure S71. [13] C NMR of compound 16b.

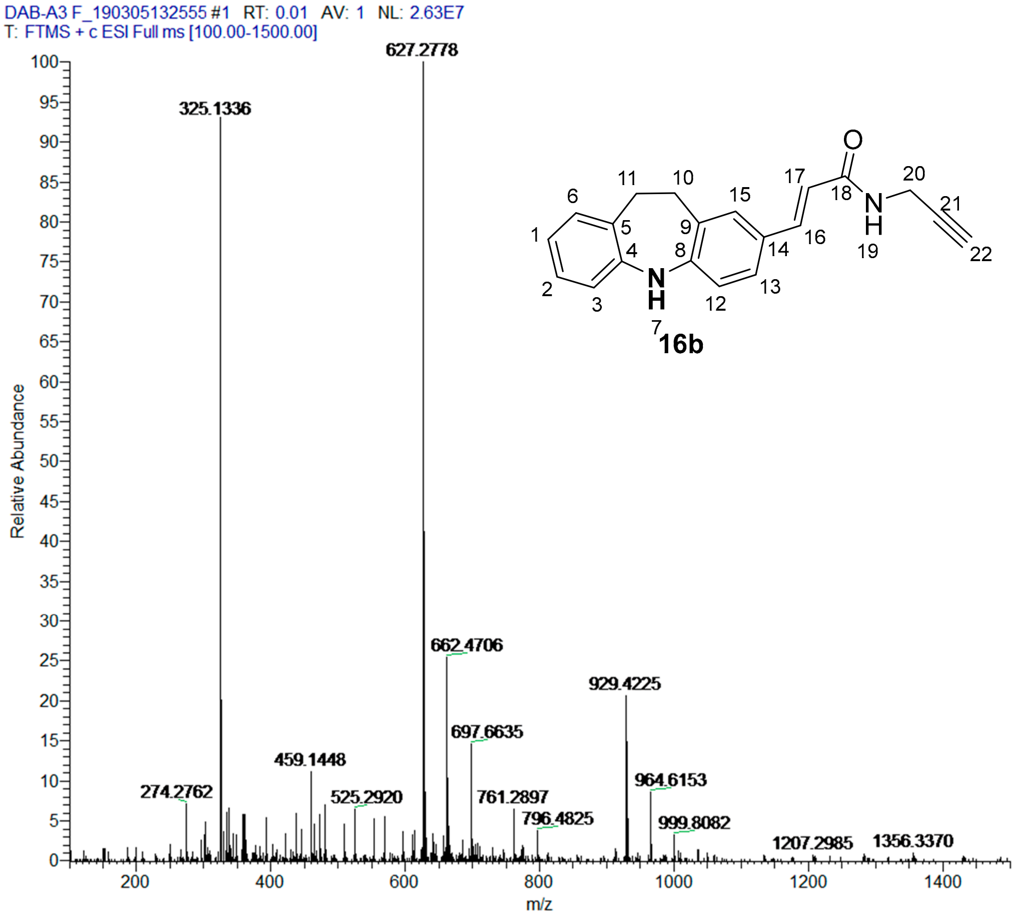

Figure S72. HR-MS of compound 16b.

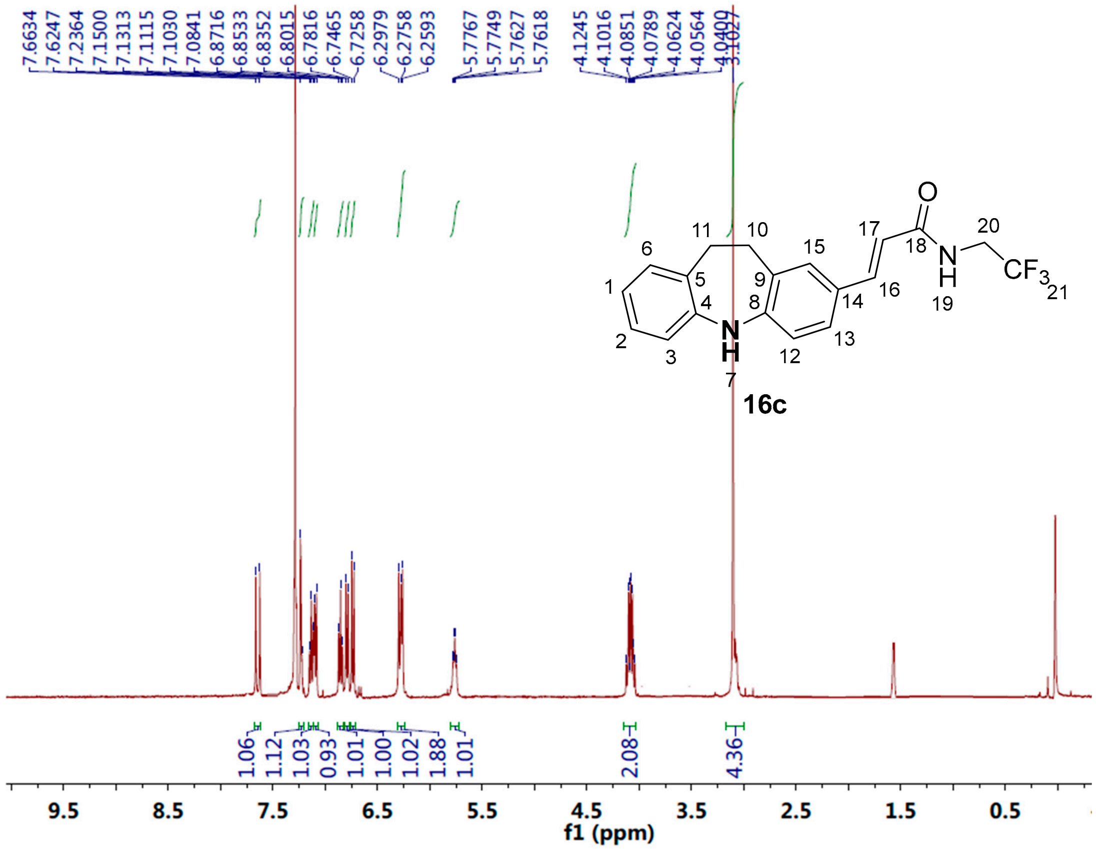

Figure S73. [1] H NMR of compound 16c.

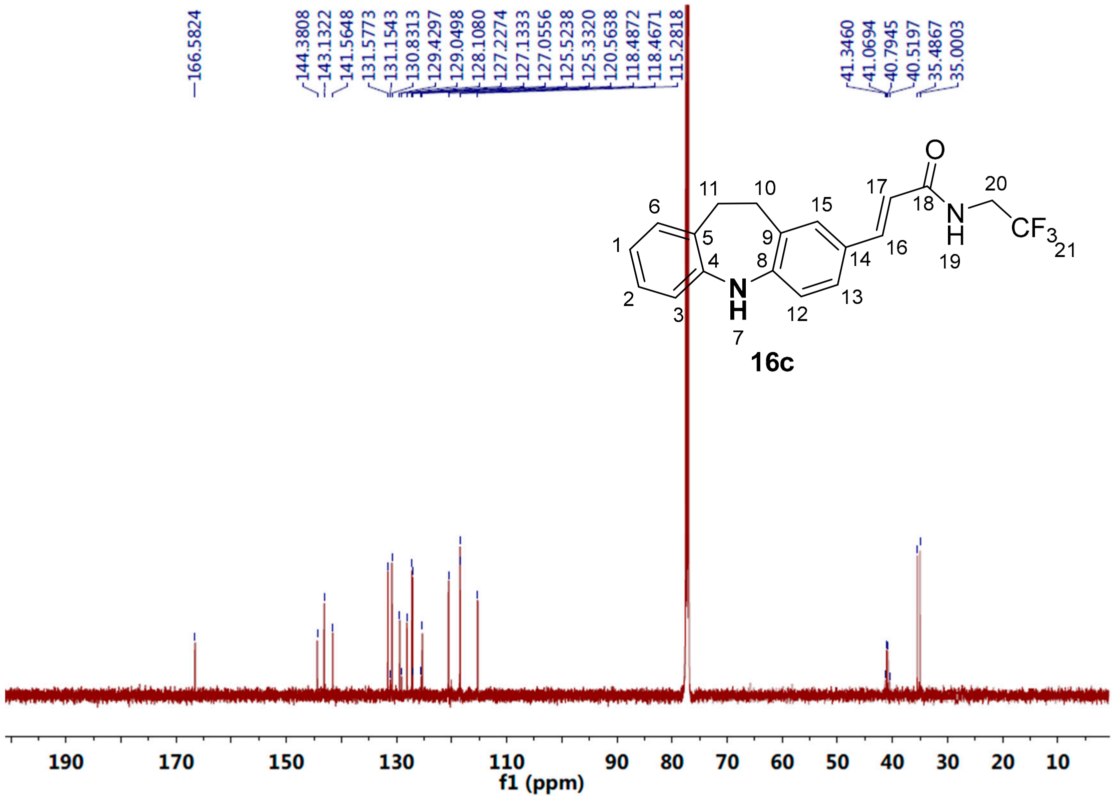

Figure S74. [13] C NMR of compound 16c.

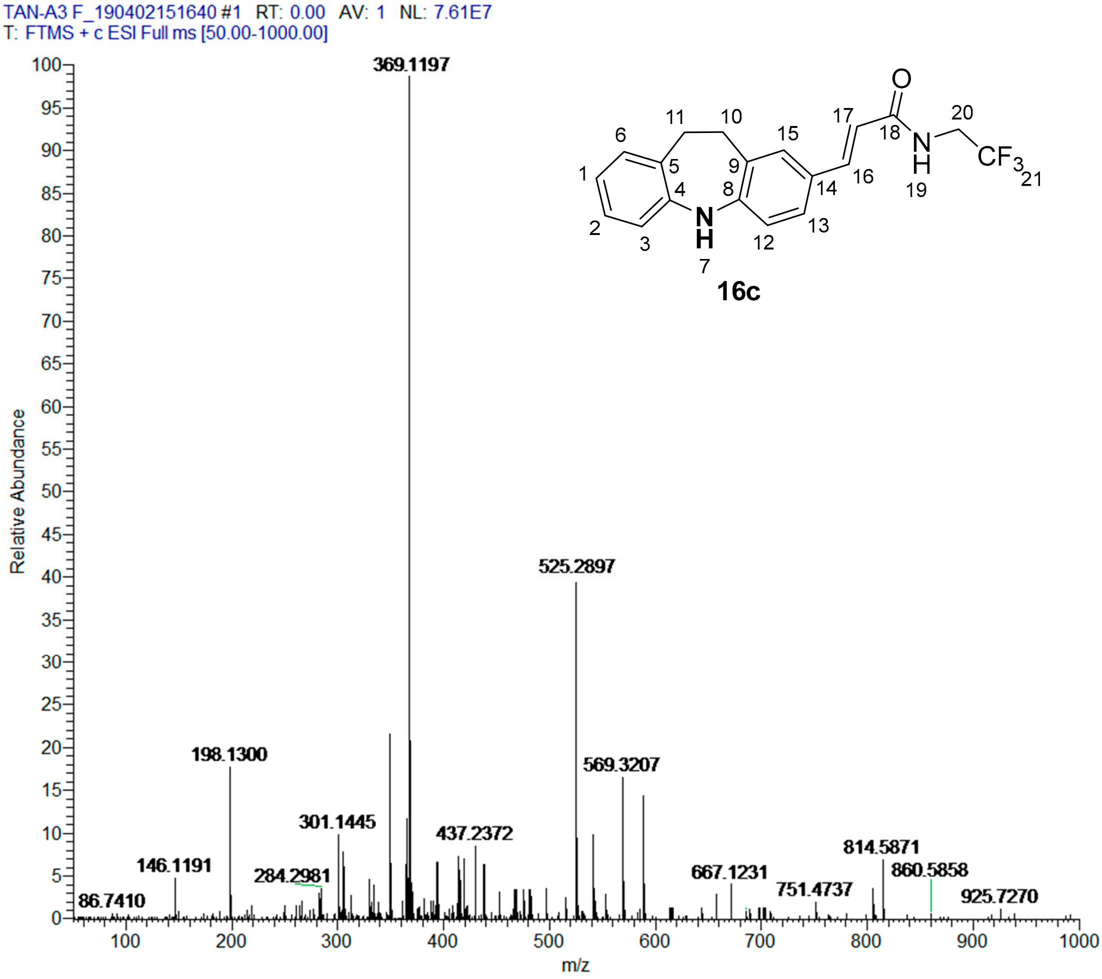

Figure S75. HR-MS of compound 16c.

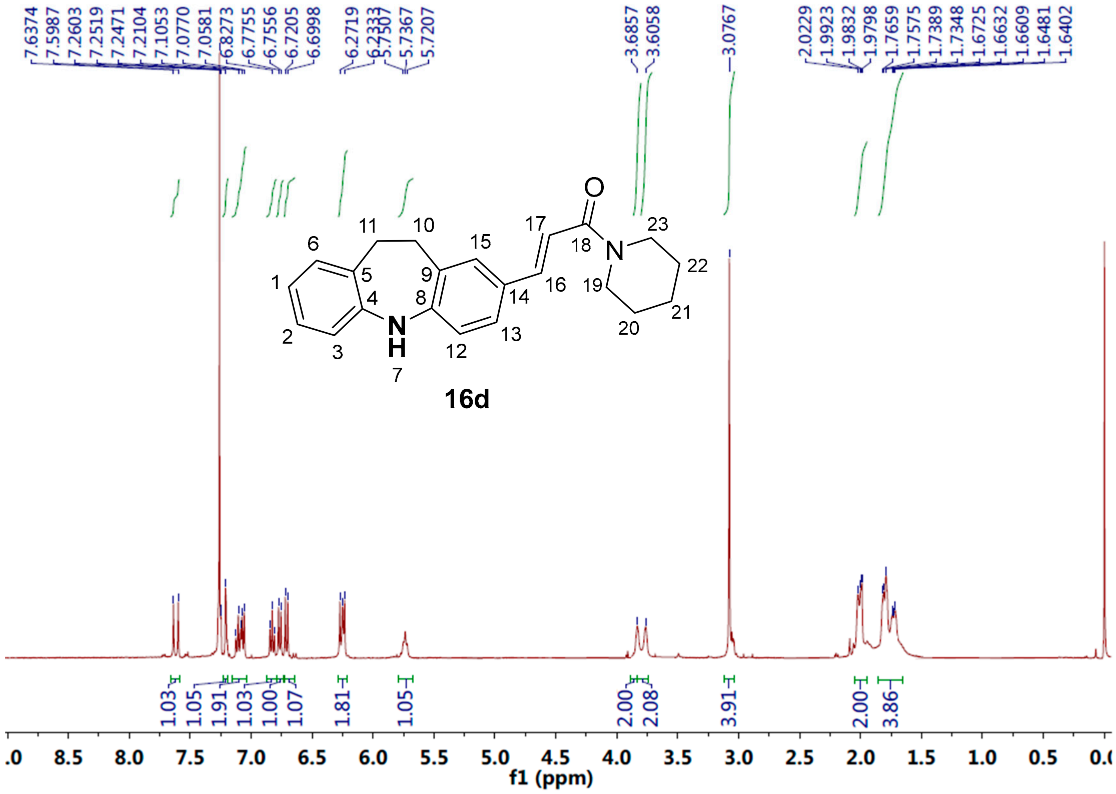

Figure S76. [1] H NMR of compound 16d.

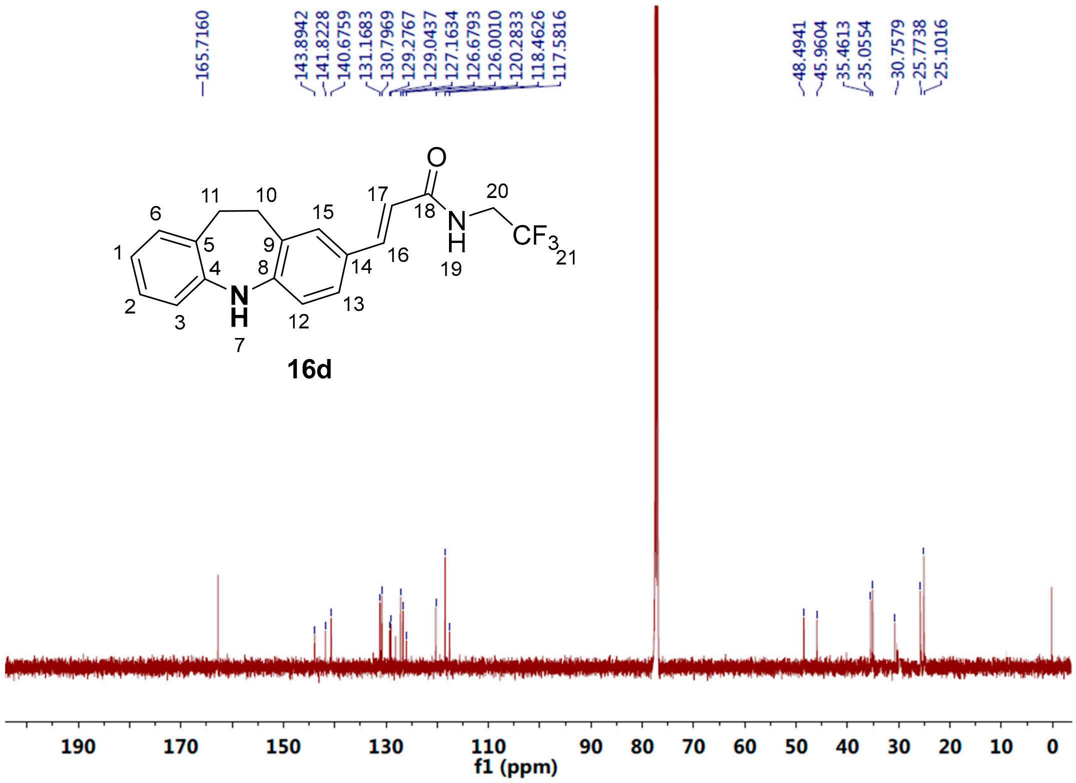

Figure S77. [13] C NMR of compound 16d.

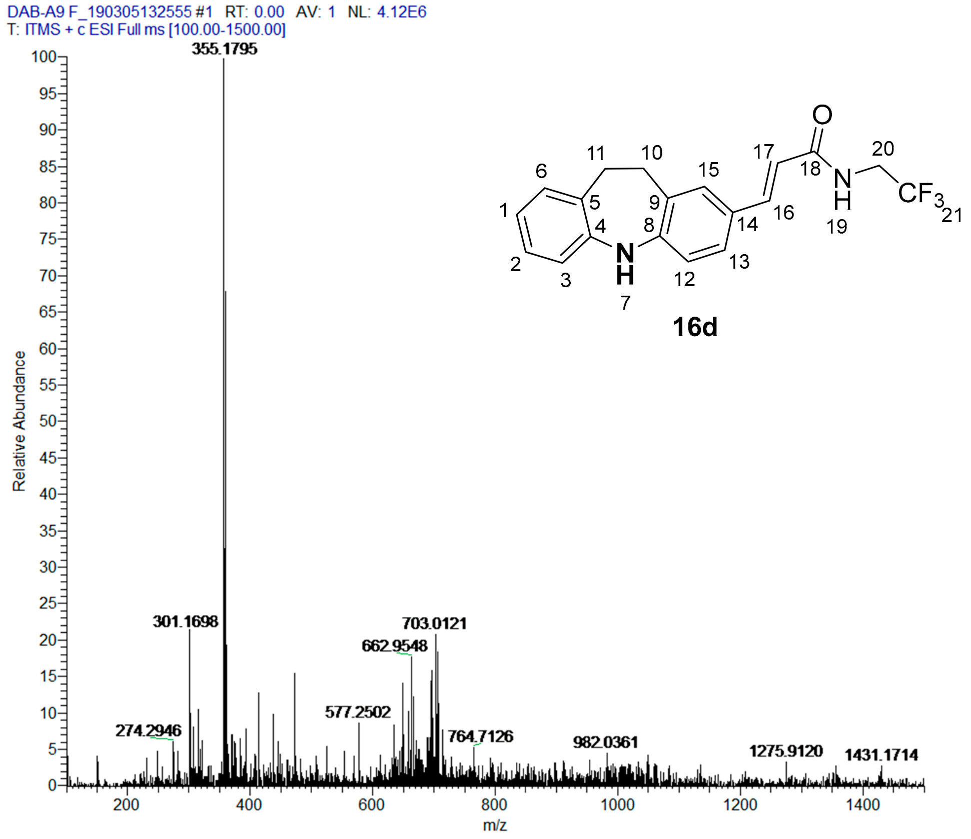

Figure S78. HR-MS of compound 16d.
